# Supplementary material for: The Development of an Age-Appropriate Fixed Dose Combination for Tuberculosis Using Physiologically-Based Pharmacokinetic Modeling (PBBM) and Risk Assessment
Source: Pharmaceutics. 2024 Dec 12;16(12):1587. doi: 10.3390/pharmaceutics16121587 (PMC11680012; doi:10.3390/pharmaceutics16121587)
Supplement: Supplementary file 1 [file pharmaceutics-16-01587-s001.zip › pharmaceutics-3304022-supplementary.pdf]

Supplementary materials for:

The development of age-appropriate fixed dose combination for tuberculosis using physiologically-based pharmacokinetic modeling (PBBM) and risk assessment

Xavier Pepin, Juliana Johansson Soares Medeiros , Livia Deris Prado , and Sandra Suarez Sharp.

LIST OF TABLES 3

LIST OF FIGURES 4

PHYSICOCHEMICAL AND BIOPHARMACEUTICAL PROPERTIES 10

SOLUBILITY VS PH 10

LOG D VS PH 10

DEGRADATION RATE VS PH 12

INH 12

RIF 12

RIF in combination with INH 13

INH in combination with RIF 13

INH in combination with reducing sugars 14

EFFECT OF BILE SALTS 14

PRECIPITATION TIME 14

DISTRIBUTION MODEL 14

CLEARANCE OF INH 15

CLEARANCE OF AC-INH 15

CLEARANCE OF RIF 17
PERMEABILITY 19
SUMMARY TABLE OF CLINICAL PK STUDIES FOR INH 19
SUMMARY TABLE OF CLINICAL PK STUDIES FOR RIF 23
RESULTS 26
ADULT PK PROFILES FOR INH 26
PEDIATRIC PK PROFILES FOR INH 33
ADULT PK PROFILES FOR RIF 39
PEDIATRIC PK PROFILES FOR RIF 44
SENSITIVITY ANALYSES 48
ISONIAZID 48
RIFAMPICIN 50
MODEL APPLICATION 52
INDIVIDUAL PK PROFILES MODEL APPLICATION FOR INH 52
COMPARISON OF POPULATION SIMULATIONS FOR INH AND RIF TO MEASURED VALUES 57
1. PHYSIOLOGICAL PARAMETERS FOR RIF ACAT MODELS 59
Z-FACTOR FITTING FOR CLINICAL PRODUCTS AND TEST PRODUCTS 60
ISONIAZID 60
RIFAMPICIN 64
CALCULATION OF MODEL PERFORMANCE INDICATORS FOR INH 68
ISONIAZID 68
1.1 RIFAMPICIN 72
2. NAT2 VMAX FOR ALL REFERENCES 75
3. FITTING DRUG PRODUCT DISSOLUTION DATA OF RIF FIXED DOSE COMBINATIONS IN AGRAWAL ET AL. [46] 76
REFERENCES 83

LIST OF TABLES

Table S1 Clinical studies used for INH PBBM validation 20

Table S2 Clinical studies used for RIF PBBM set up and validation 23

Table S3 Parameters Chosen for Sensitivity Analysis for INH 49

Table S4 Parameters Chosen for Sensitivity Analysis for RIF 50

Table S5 Z-factors for investigational batches to model INH dissolution 63

Table S6 Z-factors for investigational batches to model RIF dissolution 67

Table S7 Calculation of model prediction performance for INH C_{max} in adults 68

Table S8 Calculation of model prediction performance for INH AUC in adults 69

Table S9 Calculation of model prediction performance for INH C_{max} in pediatric subjects 70

Table S10 Calculation of model prediction performance for INH AUC in pediatric subjects 71

Table S11 Calculation of model prediction performance for RIF AUC in adult subjects 72

Table S12 Calculation of model prediction performance for RIF C_{max} in adult subjects 73

Table S13 Calculation of model prediction performance for RIF plasma concentrations in pediatric subjects 74

Table S14 Details of the bioequivalence trials of FDC formulations vs. separate formulations of anti-TB drugs conducted at NIPER bioavailability center. From [46] 76

Table S15 P-PSD for 150 mg FDC Tablet C 77

Table S16 P-PSD for 150 mg FDC Tablet D 78

Table S17 P-PSD for 150 mg FDC Tablet E 79

Table S18 P-PSD for 225 mg FDC Tablet F 80

Table S19 P-PSD for 150 mg FDC Tablet G 81

Table S20 P-PSD for 150 mg FDC Tablet H 82

LIST OF FIGURES

Figure S1 **Solubility pH Profile for Rifampicin** 10

Figure S2 **Log D vs pH Profile for Rifampicin Compared to Measured Values From Ermondi [3] and Agrawal and Pachangula [4]** 11

Figure S3 **Degradation half-life for INH alone in aqueous solution of different pH** 12

Figure S4 **Degradation half-life for RIF alone in aqueous solution of different pH** 12

Figure S5 **Degradation half-life for RIF in combination with INH in aqueous solution of different pH** 13

Figure S6 **Degradation half-life for INH in combination with RIF in aqueous solution of different pH** 13

Figure S7 **Prediction of INH and Ac-INH exposure in RA, IA and SA for the PK reported by Bing et al. [16].** 16

Figure S8 **V_{max} and Km Values Retained for the PBPK Model for Ac-INH CYP1A2** 17

Figure S9 **Fit of liver CYP3A4 and CES2 V_{max} using the data reported by Wasserman et al. [17]** 17

Figure S10 **V_{max} and Km values used for RIF clearance predictions** 18

Figure S11 **Clearance of RIF in healthy volunteers during chronic IV administration of 600 mg RIF [5].** 18

Figure S12 **Prediction of INH PK profile following administration of 1 g INH to a SA adult. Data from Boxenbaum, & Riegelman [24]** 26

Figure S13 **Prediction of INH PK profile following administration of 300 mg INH to IA and 300 mg, 600mg and 900 mg to RA population. Data from Kubota et al. [25]** 27

Figure S14 **Prediction of INH and Ac-INH PK profiles following administration of 300 mg INH to RA, IA and SA populations. Data from Bing et al. [15].** 28

Figure S15 **Prediction of INH and Ac-INH PK profiles following administration of 300 mg INH to SA populations in the fasted state and following different meal types. Data from Männisto et al. [26].** 29

Figure S16 **Prediction of INH and Ac-INH PK profiles following administration of 300 mg INH to RA populations in the fasted state and following different meal types. Data from Männisto et al. [26].** 30

Figure S17 **Prediction of INH PK profiles following administration of 10 mg/kg INH to SA populations using a slow dissolving formulation (tablet 3) and rapid dissolving formulation (tablet 4). Data from Gelber et al. [30].** 31

Figure S18 **Prediction of INH PK profiles following administration of 300 mg INH to RA and SA populations. Data from Pea et al. [27].** 31

Figure S19 **Prediction of INH PK profiles following administration of 300 mg INH to SA populations. Data from Peloquin et al. [28].** 32

Figure S20 **Prediction of INH PK profiles following administration of 10 mg/kg INH to subject 2 in fasted and following a high carbohydrate meal. Data from Melander et al. [29].** 32

Figure S21 **Prediction of INH PK profiles following administration of 150 mg INH during study STPH71/10. Test formulation = FIOCRUZ/RJ batch 09060664.** 33

Figure S22 **Prediction of INH PK profiles following administration of 150 mg INH during study STPH71/10. Ref formulation= Rifinah® Sanofi Batch A9362** ... 33

Figure S23 **Prediction of average INH PK profile at 5 mg/kg in pediatric patients. Data from Thee et al. [31]** 34

Figure S24 **Prediction of average INH PK profile at 10 mg/kg in pediatric patients. Data from Thee et al. [31]** 34

Figure S25 **Prediction of average INH PK profile at 5 mg/kg in pediatric patients. Data from Roy et al. [33]** 35

Figure S26 **Prediction of average INH PK profile at 10 mg/kg in pediatric patients. Data from Roy et al. [33]** 35

Figure S27 **Prediction of INH plasma profile at 25 mg/kg in pediatric patients. Data from Phaisal et al. [32]** 36

Figure S28 **Prediction of INH and Ac-INH plasma profile at 10 mg/kg in RA and SA pediatric patients. Data from Rey et al. [34]** 36

Figure S29 **Prediction of INH plasma profile at 10 mg/kg in RA, IA, and SA pediatric patients. Data from Schaaf et al. [35]** 37

Figure S30 **Prediction of INH plasma profile at 5 mg/kg in pediatric patients. Data from McIlleron et al. [36]** 38

Figure S31 **Prediction of INH plasma profile at 14 mg/kg in pediatric patients. Data from Bekker et al. [37]** 38

Figure S32 **Prediction of RIF plasma profile at 20 mg/kg following 1 hour infusion in pulmonary TB patients. Data from Wasserman et al. [17].** 39

Figure S33 **Prediction of RIF plasma profile at 600 mg following 1 hour infusion or oral administration. Data from Loos et al. [5].** 39

Figure S34 Prediction of RIF plasma profile after oral administration to healthy volunteers from 150 mg to 600 mg. Data from Acocella et al. [43]. 40

Figure S35 Prediction of RIF plasma profile after oral administration to healthy volunteers of 450 mg formulations C, D, E and F. Data from Agrawal et al. [46] [47] 41

Figure S36 Prediction of RIF plasma profile after oral administration to healthy volunteers of 600 mg formulations G and H. Data from Agrawal et al. [46] [47] 42

Figure S37 Prediction of RIF plasma profile after oral administration to healthy volunteers of 600 mg formulations in the fasted state, following high fat meal and acid reducing agent treatement. Data from Peloquin et al. [40] 43

Figure S38 Prediction of RIF PK profiles following administration of 300 mg RIF during study STPH71/10. Test formulation = FIOCRUZ/RJ batch 09060664. Ref formulation= Rifinah® Sanofi Batch A9362 44

Figure S38 Prediction of RIF PK profiles following IV administration of RIF. Data from Koup et al. [38] and Koup et al. [39]. 44

Figure S38 Prediction of RIF PK profiles oral administration of 300 mg/m2 RIF. Data from Koup et al. [39]. 45

Figure S38 Prediction of RIF PK profiles oral administration of 8 mg/kg RIF. Data from Schaaf et al. [42]. 45

Figure S38 Prediction of RIF PK profiles oral administration of 10 mg/kg RIF. Data from McCracken et al. [41]. 46

Figure S38 Prediction of RIF PK profiles oral administration of 10 mg/kg RIF in 4 years, 8 years and 12 years old populations. Data from Thee et al. [44]. 47

Figure S38 Prediction of RIF PK profiles oral administration of 10 mg/kg RIF in 4 years, 8 years and 12 years old populations. Data from Thee et al. [44]. 48

Figure S39 Effect of main 5 parameters on INH AUC. The horizontal solid line shows the baseline simulation and the horizontal dotted lines illustrate the boundaries for bioequivalence. 49

Figure S40 Effect of main 5 parameters on INH C_{max}. The horizontal solid line shows the baseline simulation and ther horizontal dotted lines illustrate the boundaries for bioequivalence. 50

Figure S41 Effect parameters on RIF C_{max} (left panel) and AUC_{inf} (right panel). The horizontal solid line shows the baseline simulation and the horizontal dotted lines illustrate the boundaries for bioequivalence. 51

Figure S42 Individual PK Profiles for INH in STPH71/10 (Volunteer 1 to 16) 52

Figure S43 Individual PK Profiles for INH in STPH71/10 (Volunteer 17 to 28) 53

Figure S44 Individual PK Profiles for RIF in STPH71/10 (Volunteer 1 to 16) 54

Figure S45 Individual PK Profiles for RIF in STPH71/10 (Volunteer 17 to 28) 55

Figure S46 C_{max} ratio vs AUC ratio for INH and RIF in STPH71/10 (upper panel) and for INH in STPH71/10 and STPH08/19 (lower panel) 56

Figure S47 Predicted AUC as a function of dose in pediatric groups compared to values reported in the literature (black) [31-34, 36, 37, 48, 49]. 57

Figure S48 Predicted C_{max} as a function of dose in pediatric groups compared to values reported in the literature (black) [31-34, 36, 37, 48, 49]. 57

Figure S49 Predicted RIF C_{max} as a function of dose in pediatric compared to values reported in the literature [31, 39, 41, 42, 44]. 58

Figure S50 Predicted RIF AUC in pediatric subjects compared to average exposure in pediatric and adult subjects reported in the literature [41, 49-51]. 58

Figure S51 Dissolution profile for INH from 50+75 mg INH+RIF dispersible tablet batch 2110EX047 using 900 mL pH 6.8, USP2, 50 rpm (Solubility of 153 mg/mL). 60

Figure S52 Dissolution profile for INH from 50+75 mg INH+RIF dispersible tablet batch 2111EX054 using 900 mL pH 6.8, USP2, 50 rpm (Solubility of 153 mg/mL). 60

Figure S53 Dissolution profile for INH from 50+75 mg INH+RIF dispersible tablet batch 2206EX055 using 900 mL pH 6.8, USP2, 50 rpm (Solubility of 153 mg/mL). 60

Figure S54 Dissolution profile for INH from 50+75 mg INH+RIF dispersible tablet batch Macleods NRT2104A using 900 mL pH 6.8, USP2, 50 rpm (Solubility of 153 mg/mL). 61

Figure S55 Dissolution profile for INH from 50+75 mg INH+RIF dispersible tablet batch Macleods NRT9104 using 900 mL pH 6.8, USP2, 50 rpm (Solubility of 153 mg/mL). 61

Figure S56 Dissolution profile for INH from 150+300 mg INH+RIF coated tablet batch 22070874 using 900 mL pH 6.8, USP2, 50 rpm (Solubility of 153 mg/mL). 61

Figure S57 Dissolution profile for INH from 150+300 mg INH+RIF coated tablet batch 22070875 using 900 mL pH 6.8, USP2, 50 rpm (Solubility of 153 mg/mL). 62

Figure S58 Dissolution profile for INH from 150+300 mg INH+RIF coated tablet batch Rifinah® Batch A9362 using 900 mL pH 1.2, USP1, 100 rpm (Solubility of 174 mg/mL). ... 62

Figure S59 Dissolution profile for INH from 150+300 mg INH+RIF coated tablet batch 09060664 using 900 mL pH 1.2, USP1, 100 rpm (Solubility of 174 mg/mL). ... 62

Figure S60 Dissolution profile for RIF from 50+75 mg INH+RIF dispersible tablet batch 2110EX047 using 900 mL pH 6.8, USP2, 50 rpm (Solubility of 1.2 mg/mL). ... 64

Figure S61 Dissolution profile for RIF from 50+75 mg INH+RIF dispersible tablet batch 2111EX054 using 900 mL pH 6.8, USP2, 50 rpm (Solubility of 1.2 mg/mL). ... 64

Figure S62 Dissolution profile for RIF from 50+75 mg INH+RIF dispersible tablet batch 2206EX055 using 900 mL pH 6.8, USP2, 50 rpm (Solubility of 1.2 mg/mL). ... 64

Figure S63 Dissolution profile for RIF from 50+75 mg INH+RIF dispersible tablet batch Macleods NRT2104A using 900 mL pH 6.8, USP2, 50 rpm (Solubility of 1.2 mg/mL). ... 65

Figure S64 Dissolution profile for RIF from 50+75 mg INH+RIF dispersible tablet batch Macleods NRT9104 using 900 mL pH 6.8, USP2, 50 rpm (Solubility of 1.2 mg/mL). ... 65

Figure S65 Dissolution profile for RIF from 150+300 mg INH+RIF coated tablet batch 22070874 using 900 mL pH 6.8, USP2, 50 rpm (Solubility of 1.2 mg/mL). ... 65

Figure S66 Dissolution profile for RIF from 150+300 mg INH+RIF coated tablet batch 22070875 using 900 mL pH 6.8, USP2, 50 rpm (Solubility of 1.2 mg/mL). ... 66

Figure S67 Dissolution profile for RIF from 150+300 mg INH+RIF coated tablet batch Rifinah® Batch A9362 using 900 mL pH 7.4, USP1, 100 rpm (Solubility of 1.79 mg/mL) after data normalization to 100%. ... 66

Figure S68 Dissolution profile for RIF from 150+300 mg INH+RIF coated tablet batch 09060664 using 900 mL pH 7.4, USP1, 100 rpm (Solubility of 1.79 mg/mL) after data normalization to 100%. ... 66

Figure S69 Predicted versus measured C_{max} in pediatric subjects. Error bars show one standard deviation ... 70

Figure S70 Predicted versus measured AUC in pediatric subjects. Error bars show one standard deviation ... 71

Figure S71 Predicted versus measured plasma concentrations in pediatric subjects for RIF. Error bars show one standard deviation. ... 74

Figure S72 P-PSD Fitting for 150 mg FDC Tablet C Using pH2 Dissolution Method at 50 rpm 77

Figure S73 P-PSD Fitting for 150 mg FDC Tablet D Using pH2 Dissolution Method at 75 rpm (upper panel) and 30 rpm (lower panel) 78

Figure S74 P-PSD Fitting for 150 mg FDC Tablet E Using pH2 Dissolution Method at 50 rpm 79

Figure S75 P-PSD Fitting for 225 mg FDC Tablet F Using pH2 Dissolution Method at 75 rpm 80

Figure S76 P-PSD Fitting for 150 mg FDC Tablet G Using pH2 Dissolution Method at 30 rpm 81

Figure S77 P-PSD Fitting for 150 mg FDC Tablet H Using pH2 Dissolution Method at 50 rpm 82

PHYSICOCHEMICAL AND BIOPHARMACEUTICAL PROPERTIES

SOLUBILITY VS PH

In the commercial formulations, isoniazid and rifampicin are crystalline drugs. The solubility vs pH measured for isoniazid is high (above 150 mg/mL across the physiological pH range) [1]. The measured solubility vs pH for rifampicin is taken from the literature [2] and the solubility vs pH profile is shown in Figure S1.

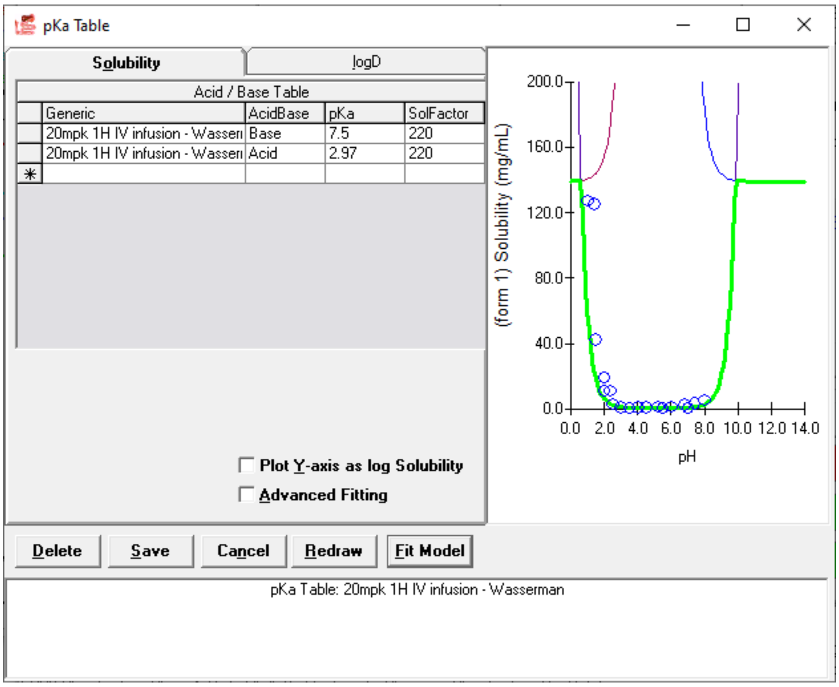

Figure S1 Solubility pH Profile for Rifampicin

The solubility vs pH values reported for RIF match very well the theoretical solubility- pH profile predicted from Henderson Hasselbalch equations and the pKa values of the drug. This drug is zwitterionic and, in the stomach, the solubility- pH profile shows a steep curve which necessitated recalculating the impact of fluid volume administered with each test treatment or co-administration of food, or acid reducing agents on the final stomach pH. The age group and the resting fluid volume in the stomach were also considered.

LOG D VS PH

The INH Log D vs pH relationship was calculated using the default parameters in GastroPlus and the measured Log P values. The log P and log D reported for Rifampicin in the literature

are highly variable and sometimes show behavior contrary to the ionization of the drug [3] (**Figure S2**).

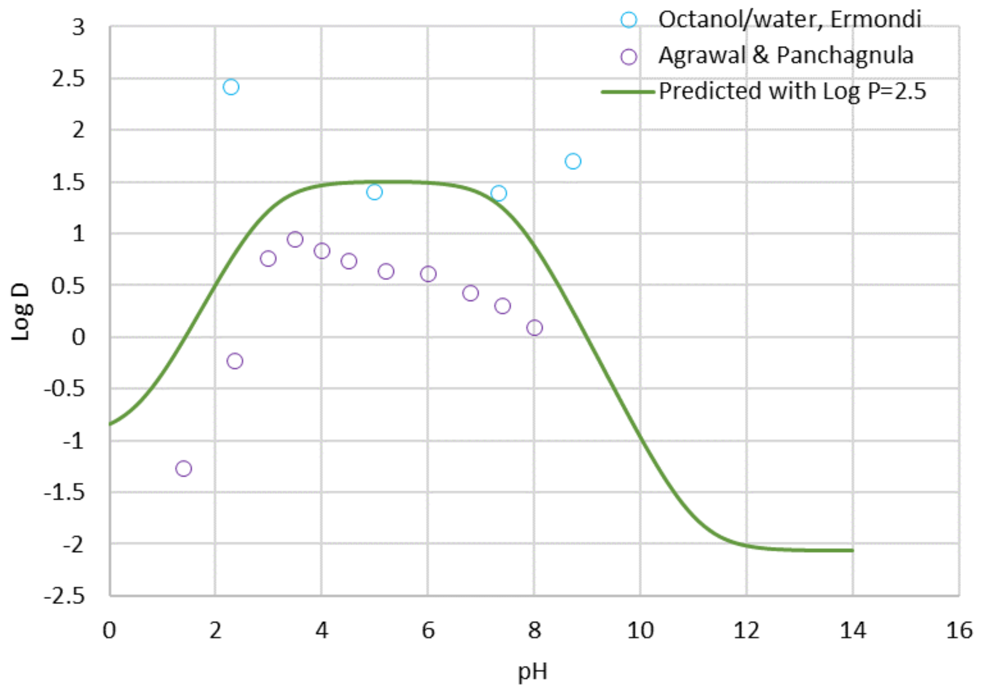

Figure S2 Log D vs pH Profile for Rifampicin Compared to Measured Values From Ermondi [3] and Agrawal and Pachangula [4]

ADMET predictor calculated a value of 2.5. A log P of 1 was used to calculate the volume of distribution for the PK study published by Loos et al. [5] for one volunteer. This value is close to that reported by Agrawal and Panchagnula [4]. Based on all other studies, a log P value was fixed to 1.5. This value is compatible with the data reported by Ermondi et al. [3] for the pH range where the drug should be zwitterionic.

DEGRADATION RATE VS PH

Rifampicin is known to degrade as a function of pH whilst isoniazid is relatively stable to pH. However, when both actives are associated, RIF reacts with INH to form a degradation product and therefore the stability of INH in the presence of RIF is impacted. Also, INH shows instability with reducing sugars.

INH

The aqueous degradation half-life versus pH for INH was measured by Chen [6] and is shown in Figure S3.

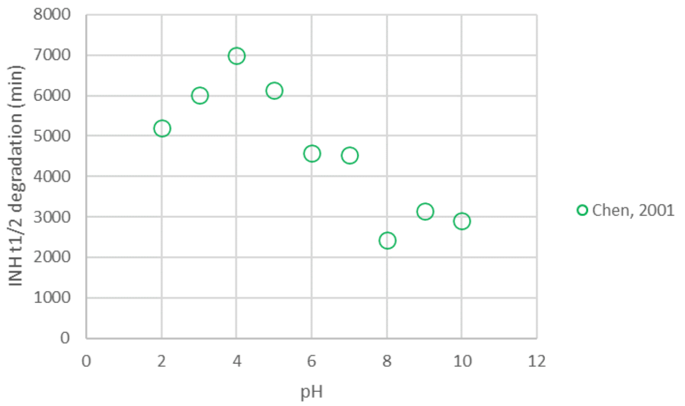

Figure S3 Degradation half-life for INH alone in aqueous solution of different pH

RIF

The aqueous degradation half-life versus pH for RIF was measured by Chen et al. [6], Singh et al. [7], Seydel et al. [8] and Prankerd et al. [9]. It is shown in Figure S4.

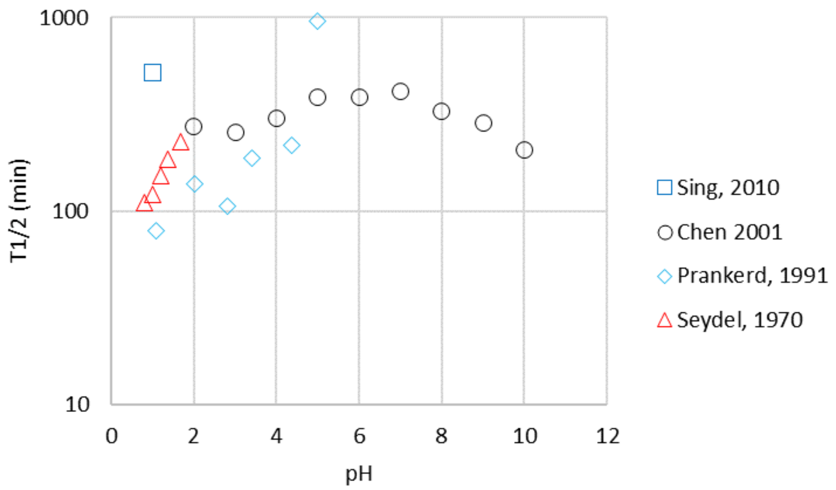

Figure S4 Degradation half-life for RIF alone in aqueous solution of different pH

RIF in combination with INH

The aqueous degradation half-life versus pH for RIF in combination with INH was measured by Mariappan [10], and Farmanguinhos. It is shown in Figure S5. The Mariappan data was retained as a worst-case scenario for simulation of fixed dose combinations.

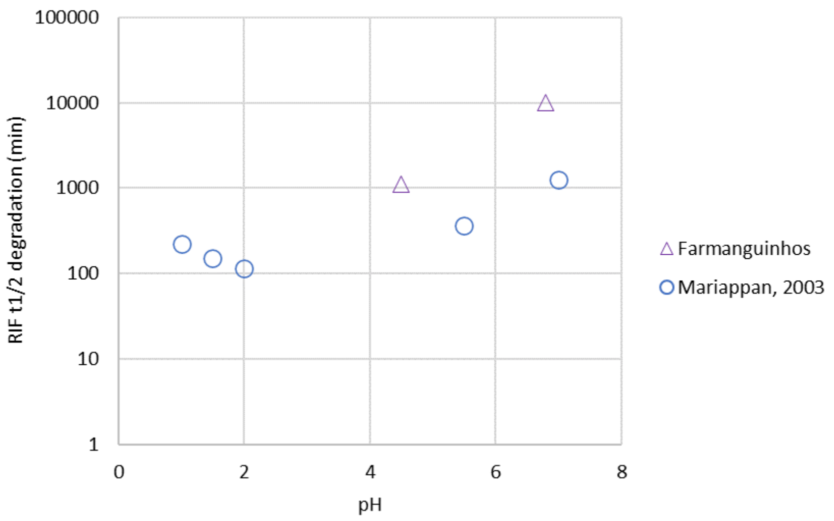

Figure S5 Degradation half-life for RIF in combination with INH in aqueous solution of different pH

INH in combination with RIF

The aqueous degradation half-life versus pH for INH in combination with RIF was measured by Mariappan [10], Singh [7], and Farmanguinhos. It is shown in Figure S6.

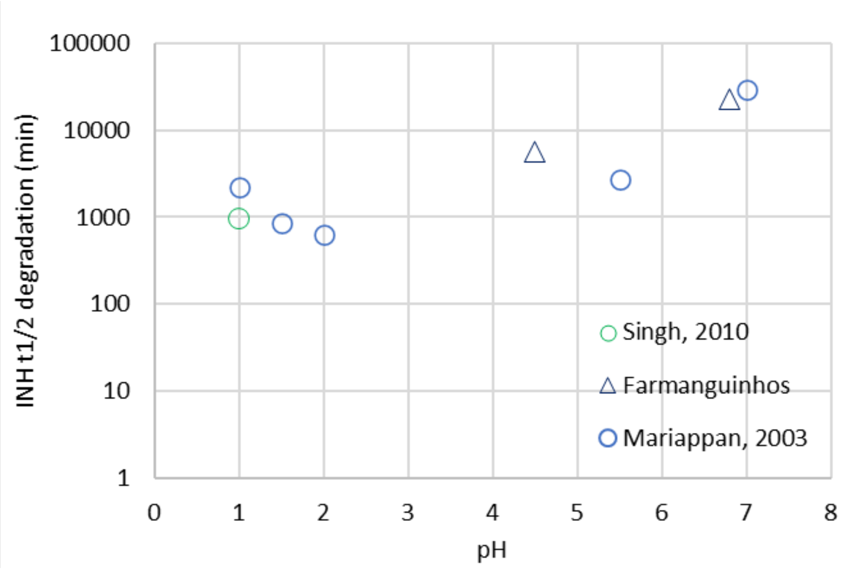

Figure S6 Degradation half-life for INH in combination with RIF in aqueous solution of different pH

The Mariappan data was retained as a worst-case scenario for simulation of fixed dose combinations.

INH in combination with reducing sugars

INH in combination with reducing sugars will form hydrazones which may reduce the drug's shelf life as a solid dosage form but also may react in solution over a short period of time in vitro or in vivo. The formation rate of hydrazones is dependent on the type of reducing sugar and the pH of the solution. The degradation of INH at 37°C in the presence of $1 \cdot 10^{-2}$ M INH and $5 \cdot 10^{-2}$ M sugar was measured by Devani et al. [11]. The degradation rate of INH in presence of reducing sugars is used to compute the effect of carbohydrate-rich diets on the pharmacokinetics of INH in human [12].

EFFECT OF BILE SALTS

Human fasted physiology was used throughout the modeling and no changes were made from the default values. The z-factor dissolution model used for RIF and INH. The effect of bile salt was predicted for INH using the theoretical equation based on Log P and is fitted to the measured FaSSIF solubility value of 1.39 mg/mL for RIF [2]. A value of 1.54E+4 for the bile salt solubilization ratio is applied to predict RIF in vivo dissolution.

PRECIPITATION TIME

There is no risk of precipitation for isoniazid since its solubility is high and is not significantly affected by pH. However, RIF shows a strong pH-dependent solubility and potentially can precipitate following pH change from the stomach to the intestinal environment. Simulations conducted for RIF oral PK have shown that the precipitation is likely for higher doses in pediatric patients and was included in the model assuming the formation of precipitated particles with 25 μm radius and a first order precipitation rate constant of 500 seconds. This parameter is verified during model validation for pediatric profiles in combination with lower volumes in the GI tract. In addition, in the stomach for RIF, the effect of beverage, volume administration and pre-treatment with ARA must be considered to calculate the stomach pH.

DISTRIBUTION MODEL

The presence of human intravenous PK data for INH and RIF allowed to verify the full PBPK options selected for both drugs. The default Lukacova method for K_{ps} calculation and V_d determination based on physico-chemical properties of INH, Ac-INH and RIF was used with no adjustments.

CLEARANCE OF INH

Isoniazid clearance is the sum of renal clearance and metabolic elimination. The renal clearance was estimated by the product of glomerular filtration rate (GFR) and fraction of unbound drug in the plasma ($f_{u,p}$) for all the populations (adult or pediatric) using full PBPK models. Isoniazid acetylation is mediated in human by Arylamine N-acetyltransferase 2 (NAT2), MW = 33.6kDa. INH acetylation was introduced in the model as the sole metabolic elimination pathway using the following approach. The details of the NAT2 ontogeny model are shown by Pepin et al. [12]. In order to model INH metabolism in populations where the NAT2 genotypes are varied, an analysis of the worldwide distribution of RA, IA and SA individuals was undertaken based on literature reports [13-15]. This information is used to predict population PK and for risk analysis. Details of the NAT2 distribution is provided in the supplementary materials and in [12]. The K_m for NAT2 were fitted to ascending dose studies of INH and the V_{max} was adapted to each genotypes as explained in [12].

CLEARANCE OF AC-INH

Acetyl-Isoniazid is anticipated to be cleared by CYP1A2 as per AP v10.3. All the default parameters predicted by AP v10.3 were kept as input parameters in the PBPK model for this drug. The only parameter that was adjusted was the PBPK V_{max} for the metabolism of Ac-INH. The initial prediction for CYP 1A2 Ac-INH V_{max} was initially calculated by AP v10.3 at 0.00184 mg/s/mg-enz. It was adjusted to 0.001288 mg/s/mg-enz to match the RA population reported by Bing et al. for the Chinese population [16]. In addition, this enzyme V_{max} was also applied to IA and SA to check whether the PK profile of Ac-INH could be well predicted following 300 mg INH administration in the fasted state. In addition to this metabolic clearance, a renal clearance given by the product of fraction of drug unbound in the plasma and glomerular filtration rate was applied to all populations. The adjustment made for CYP1A2 Ac-INH V_{max} is shown in **Figure S7**, where the PK profiles of INH and Ac-INH in RA, IA, and SA are predicted and compared to data reported by Bing et al. [16]. The values retained for Ac-INH CYP1A2 V_{max} and Km are shown in Figure S8.

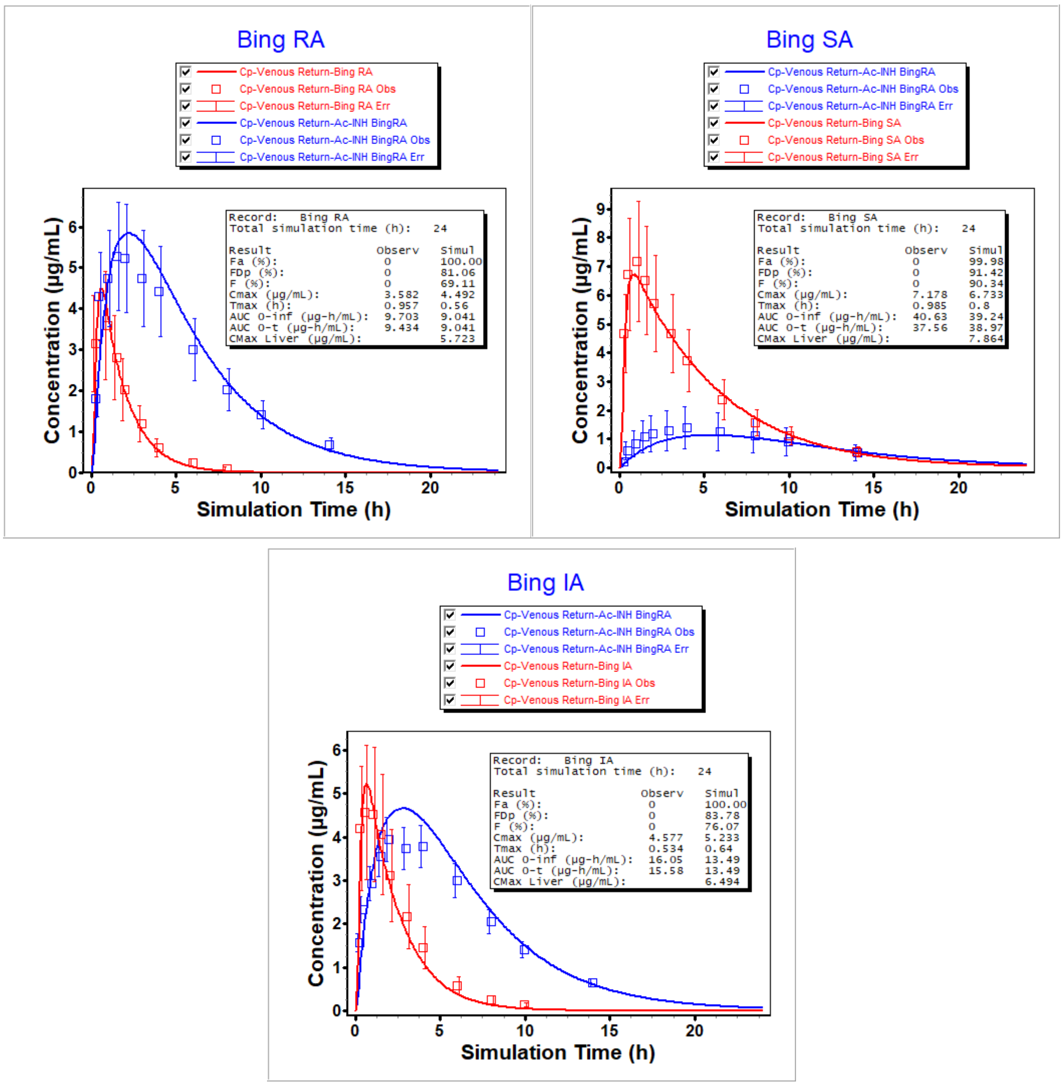

Figure S7 Prediction of INH and Ac-INH exposure in RA, IA and SA for the PK reported by Bing et al. [16].

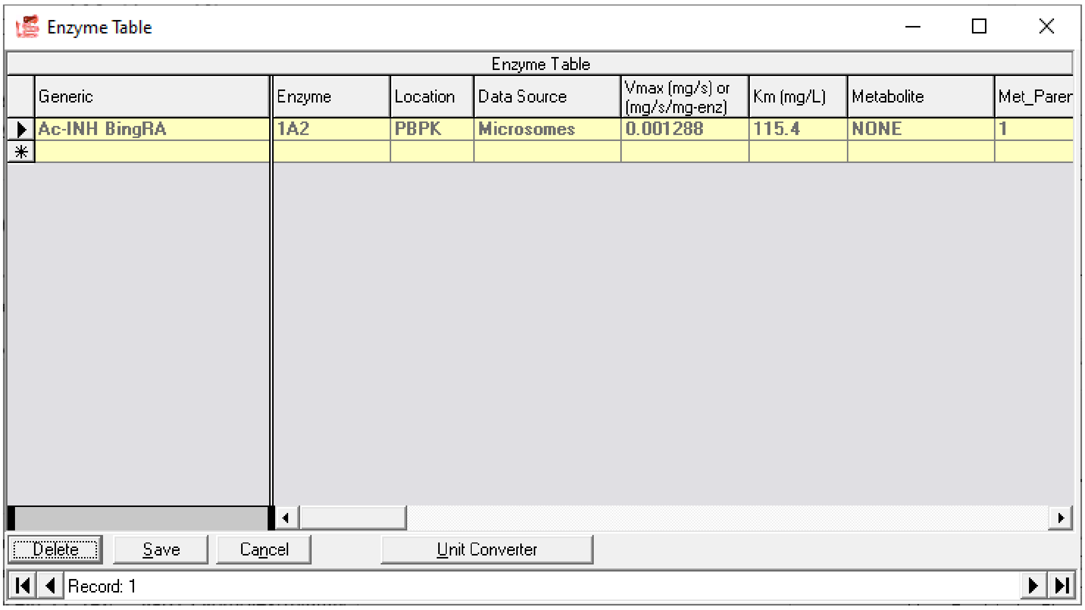

Figure S8 **V_{max} and Km Values Retained for the PBPK Model for Ac-INH CYP1A2**

CLEARANCE OF RIF

In order to derive V_{max} values for liver CYP3A4 and CES2, the two enzymes responsible for RIF liver metabolism, the Km values predicted for CYP3A4 by AP v10.3 were applied to both enzyme isoforms and the V_{max} was fitted to the PK profiles obtained in adult TB patients by Wasserman et al. [17] (**Figure S9**).

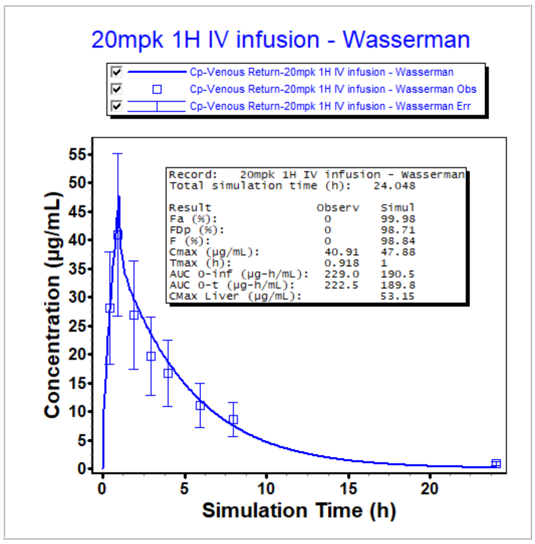

Figure S9 **Fit of liver CYP3A4 and CES2 V_{max} using the data reported by Wasserman et al. [17]**

The gut CYP3A4 V_{max} was fitted to the data reported by Loos et al. for oral administration. The resulting V_{max} and Km values used for RIF clearance prediction are shown in **Figure S10**.

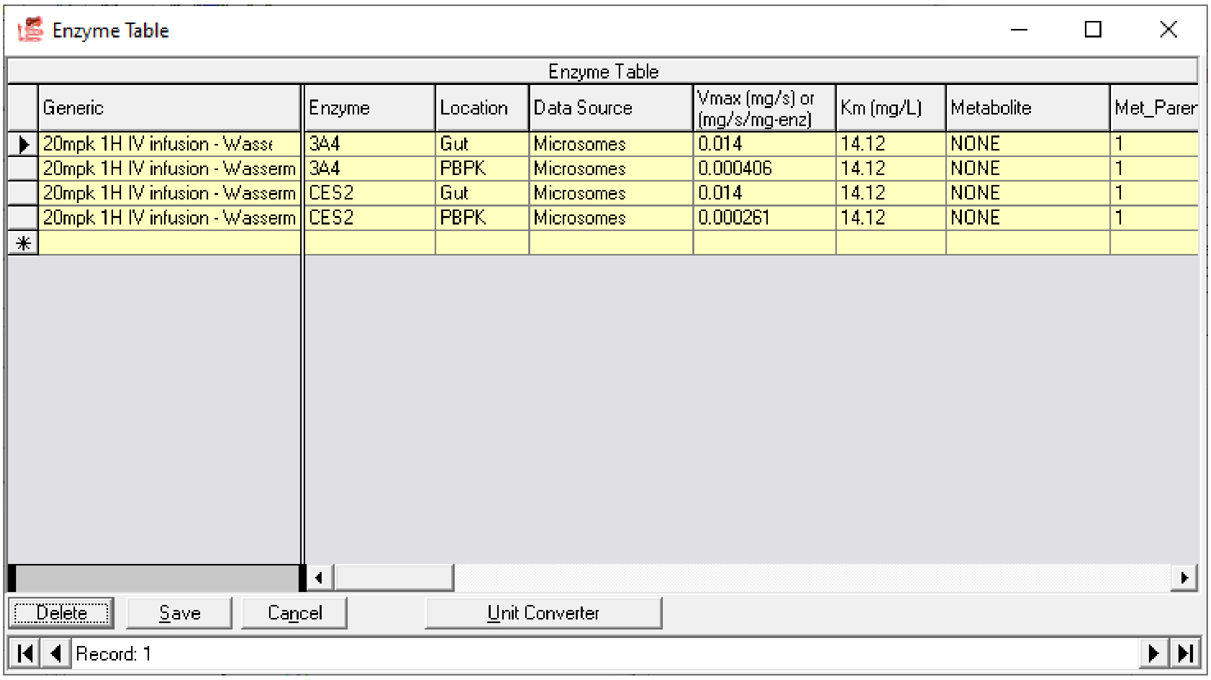

Generic	Enzyme	Location	Data Source	Vmax (mg/s) or (mg/s/mg-enz)	Km (mg/L)	Metabolite	Met_Parer
20mpk 1H IV infusion - Wass	3A4	Gut	Microsomes	0.014	14.12	NONE	1
20mpk 1H IV infusion - Wasserm	3A4	PBPK	Microsomes	0.000406	14.12	NONE	1
20mpk 1H IV infusion - Wasserm	CES2	Gut	Microsomes	0.014	14.12	NONE	1
20mpk 1H IV infusion - Wasserm	CES2	PBPK	Microsomes	0.000261	14.12	NONE	1

Figure S10 **V_{max} and Km values used for RIF clearance predictions**

RIF in a substrate and an inducer of CYP3A4 and the clearance of RIF increases over time. Loos et al. have shown in TB patients that upon chronic daily treatment of 600 mg RIF, the clearance increased by 73% on day 22 compared to day 1 [5]. The steady-state for RIF induction of its own metabolizing enzymes is anticipated to be between 7 and 10 days [18].

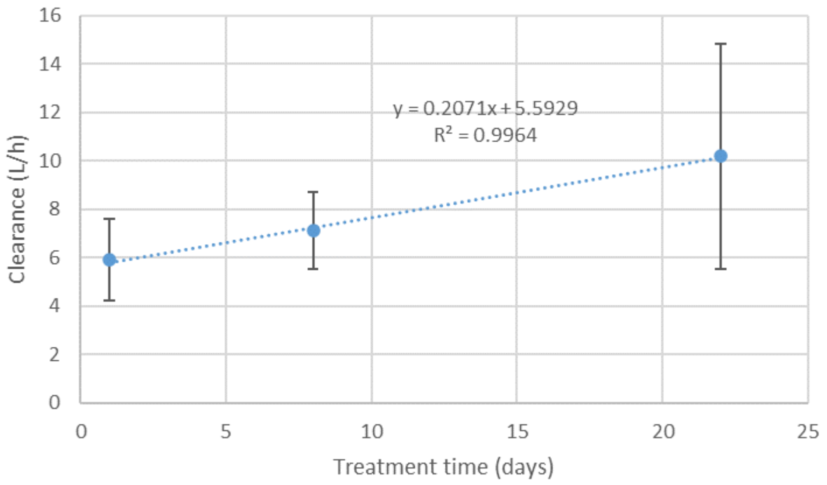

Figure S11 **Clearance of RIF in healthy volunteers during chronic IV administration of 600 mg RIF [5]**

In order to mimic steady state exposure in the target population, the V_{max} of gut and liver CYP3A4 and CES2 were multiplied by 1.7 in the software to reproduce the apparent increase

in clearance at steady state. The steady state for RIF induction of its own metabolizing enzymes is anticipated to be between 7 and 10 days [18].

PERMEABILITY

Isoniazid has been incorrectly classified as poorly permeable based on its low log P values [19, 20]. Some literature reports medium permeability for INH [1], However, the Caco2 data or MDCK data reported from studies where both isoniazid and rifampicin are tested or where isoniazid is tested against references, demonstrate that isoniazid apparent permeability is 6-fold higher than that measured for rifampicin [21]. Final P_{app} value of 57.9 10^{-7} cm/s reported for rifampicin by Biganzoli et al. [22] and a 6 fold higher value for isoniazid, 347 10^{-7} cm/s were utilized as input. A power correlation of P_{app} to P_{eff} previously reported by Andreas et al. [23] was utilized to predict a rifampicin effective permeability of 2.11 10^{-4} cm/s, and effective permeability of INH equal to 6.7 10^{-4} cm/s. In addition, there is no polarity to the permeability of RIF in the 5-200 μg/mL concentration range or INH in the 70-7000 μg/mL concentration range, which rules out active influx or efflux transporters [21].

RIF is a substrate of P-gp and an inducer of P-gp. As shown previously, this effect is responsible for variable volume of distribution amongst different populations and over repeated dosing with an increase in the volume of distribution. In addition, in neonates, the volume of distribution is comparatively larger than in children above 1 year of age, due to the ontogeny of this transporter. However, with regard to intestinal absorption, none of the oral PK profiles were indicative of a major P-gp effect and therefore, it was not included in the model. The drug permeability is relatively high even when considering only the A to B permeability and therefore this effect is neglected.

SUMMARY TABLE OF CLINICAL PK STUDIES FOR INH

The summary of clinical PK studies used for INH PBBM setup and validation is shown in **Table S1**.

Table S1 Clinical studies used for INH PBBM validation

Study #	Country	Population	Drug(s) and posology	Outcome	Use in the PBBM	Literature reference
1	USA	2 adult healthy volunteers Subject A (white male, 30 years 69 kg, 165 cm height) and subject B (white male, 50 years, 80kg and 181 cm height). Subject A is a SA and Subject B a RA.	675 mg INH injected intravenously over 5.3 min and additionally for subject A, 1 g solution in 100mL orally administered fasted	Individual PK profiles for INH provided for subject A and B.	Validation of PBPK options to calculate volume of distribution. Fitting of liver V_{max} to match SA and RA types. With oral solution, validation of oral PK	Boxenbaum, & Riegelman [24]
2	China	Healthy male subjects 24 years, 64 ± 5.82 kg. The genotype for IA, RA and SA was determined a priori	300 mg oral INH given fasted with 200 mL water.	INH and Ac-INH PK profiles and PK parameters provided	Model validation	Bing et al. [15]
3	Japan	8 healthy male volunteers (RA from genotype) 23 years, 63.5 kg, 172 cm and 8 healthy male volunteers (IA from genotype) 24 years, 61.6 kg and 168 cm.	Administration in fasted conditions of 300, 600 and 900 mg to RA subjects and 300 mg to IA subjects	The PK profiles for INH and PK parameters are provided	Model validation	Kubota et al. [25]
4	Finland	10 healthy volunteer males and females, 21-30 years, and 52-87 kg. 6 were SA and 4 were RA based on phenotype ($t_{1/2}$ elimination)	Oral administration in cross over of 300 mg INH in A: fasted state with 200 mL water, B, with 200 mL low-fat milk, C: with 300 kCal high-fat/protein meal (egg, ham, and cheese) and D: with 300 kCal high-carbohydrate meal (white bread, apple marmalade, dry pears)	The PK profiles and PK parameters for INH and Ac-INH are provided	Model validation	Männisto et al. [26]
5	Italy	26 patients with TB, 16 males, 10 females, 47 years, 62.77kg	Oral administration of 300 mg dose with rifampicin	PK profiles for INH in RA and SA	Model validation	Pea et al. [27]
6	USA	16 healthy females and males. Mean age was 39.1 ± 7.4 years, mean weight was 79.3 ± 13.2 kg. n=12 SA and n=4 RA were differentiated based on phenotype (C_{6H} INH)	Subjects received single oral doses of INH 300 mg. They also received600 mg RIF, 30 mg/kg PZA (median 2386 mg), and 25 mg/kg EMB (median 1950 mg).	Average PK profiles and parameters for INH provided	Model validation	Peloquin et al. [28]
7	Sweden	9 male volunteers (28-35 years), 67-77 kg weight range. acetylators	Fasted administration of 10 mg/kg using 100 mg tablet with 100 mL water. Fed state administration immediately after a 440 kCal meal	Individual PK profiles for INH in fasted and fed states	Model validation	Melander et al. [29]

			comprising 45% carbohydrates, 20% protein and 35% fat.			
8	USA	6 human volunteers	Fasted administration of 10 mpk of each of 6 formulations of INH to human volunteers + IV 5 mpk bolus injection. Mixture of acetylators	IV and oral individual PK profiles	Integration of dissolution in the PBBM, validation of the IV model	Gelber et al. [30]
9	Brazil	28 healthy volunteers, randomized 2 period crossover bioequivalence study Washout of 1 week, 46% males, 37 years, 68.44 kg, 1.67 m height. SA fraction assumed at 0.5 based on terminal elimination of INH	-10H to +4H Fasted crossover administration of 150 mg INH+300 mg RIF. Test = FIOCRUZ/RJ batch 09060664. Ref = Rifinah® Sanofi Batch A9362 with 200 mL water. No water until +2H	Oral individual PK profiles for INH and RIF + subjects covariates and in vitro dissolution for dosage forms	Examination of individual PK profiles and understanding risks related to physiological variables	STPH71/10
10	Brazil	42 healthy volunteers	-10H to +4H Fasted crossover administration of 100mg INH. Test = FIOCRUZ/RJ batch 18060194. Ref = Isozid® Riemser Pharma GmbH batch 002017 with 200 mL water. No water until +2H	Individual PK profiles and parameters for INH	Examination of individual profiles	STPH08/19
11	South Africa	20 TB patients, 1.09 (0.49) year old, 45% of females. Genotyping revealed that 8 children were RA (5 of 8 children <1 year old), 4 were IA (3 of 4 children <1 year old), and 8 were SA (4 of 8 children <1year old).	Administration of Rimcure® FDC (30 mg INH + 60mg RIF + 150 mg PZA) to target 5mg/kg and 10 mg/kg INH	4 datapoint Plasma profiles up to 5 hours of INH for 5mg/kg and 10 mg/kg, C_{max} and AUC_{0-5} by acetylator status	Model validation in pediatric patients	Thee et al. [31]
12	Thailand	12 children 2.1-4.9 years, 7 males 5 females, 90-121 cm and 10.2-29 kg. Five participants were NAT2 SAs	isoniazid 25 mg/kg and rifapentine dose 25.7 mg/kg	Individual INH profiles in children	Model validation in pediatric patients	Phaisal et al. [32]
13	India	Twenty children suffering from pulmonary TB in the age group 6-12 years. The mean bodyweight was 17.6 kg (range 15-21 kg). No information provided on the acetylator types	A three-drug oral antitubercular regimen comprising of rifampicin (10 mg/kg), pyrazinamide (30mg/kg) and isoniazid in a dose of either 10 mg/kg (Group I) or 5 mg/kg (Group II) was administered for fourteen days. INH was given fasted at 6 a.m. followed one hour later by rifampicin and 6 hour later by pyrazinamide. A standard breakfast meal was given at 8 a.m.	Group I: INH C_{6h}= 2.69 ± 0.44 ug/ml (range 0.46-5.01 ug/ml), Group II: INH C_{6h}= 1.31 ±0.08 ug/ml (range 0.72-1.72 ug/ml). Average PK profiles for INH provided	Model validation in pediatric population	Roy et al. [33]

14	France	Thirty-four children participated in the study. Two of them were 0±1 month old, 17 were 2.4±19 months old-and 15 were 26±196 months old. 17 patients were considered as slow acetylators and 17 as fast acetylators from phenotype (metabolic ratio)	A single dose of 10 mg/kg was administered as powder in water to fasting patients	Individual C_{max} and T_{max} values for INH and Ac-INH provided. Average PK profiles in SA and RA groups for INH and Ac-INH provided	Model validation in pediatric population	Rey et al. [34]
15	South Africa	64 children <13 years of age (median 3.8) with respiratory tuberculosis. The NAT2 genotype was determined. 25 children were classified as homozygous slow (SS) 2.8 years, 24 as heterozygous fast (FS) 3.9 years, and 15 as homozygous fast (FF) acetylators 4.1 years	The INH powder was dissolved in 5–10 ml of water and administered by one of the study personnel orally with a syringe or, in the case of very young children, through a nasogastric tube and washed down with water. A light breakfast was permitted 60–90 minutes later.	INH concentrations were determined 2–5 hours after a 10 mg/kg INH dose	Model validation in pediatric patients	Schaaf et al. [35]
16	South Africa	56 hospitalized children with severe forms of TB (median age, 3.22 years; interquartile range [IQR], 1.58–5.38 years, 3 months to 13 years) 36% were SA, 43% were IA and 14% were RA. 52% were males and 39% were infected by HIV	Isoniazid was administered as part of dispersible, pediatric, fixed-dose combinations (Rimcure Paed 3-FDC: each tablet contains 60 mg of rifampin, 30 mg of isoniazid, and 150 mg of pyrazinamide; Rimactazid Paed 60/30: each tablet contains 60 mg of rifampin and 30 mg of isoniazid;or Rimactazid Paed 60/60: each tablet contains 60 mg of rifampin and 60 mg of isoniazid	C_{max}, $AUC_{0\text{-}6H}$ are reported by acetylator group and by dose range	Model validation in pediatric patients	McIlleron [36]
17	South Africa	39 infant patients, mean age 6.6 months, 6.45 kg. No genotyping	RIF [10-20 mg/kg], INH [10-15 mg/kg], PYR [30-40 m/kg] and ETH [15-25 mg/kg] were co-administered after a 2hour fast via nasogastric tube as a suspension.	Average INH profile in infants after 1, 2, 4, 6, and 8 h after 13.8mg/kg	Model validation in pediatric patients	Bekker et al. [37]

SUMMARY TABLE OF CLINICAL PK STUDIES FOR RIF

The summary of clinical PK studies used for RIF PBBM set up and validation is shown in **Table S2**.

Table S2 Clinical studies used for RIF PBBM set up and validation

Study #	Country	Population	Drug(s) and posology	Outcome	Use in the PBBM	Literature reference
1	South Africa	46 pulmonary TB patients 39 years (30-47) 61 kg (53-80) BMI 23 kg/m2 (19-32) with HIV co-infection	Oral 10 mpk (with FDC Rifafour [Sanofi]) Oral 35 mpk RIF (10 mpk made of rifafour [Sanofi] + Rimactane at 150mg [Sandoz] and Eremfat at 600mg [Riemser] 20mpk IV RIF	Average PK profiles for n=14 to n=17 reported for RIF for each RIF treatment + PK parameters	Top-down analysis of IV PK to determine CYP3A4 and CES2 V_{max}<br>Oral dose to check PBPK model	Wasserman et al. [17]
2	Germany	Two groups of six patients with active pulmonary tuberculosis, 83% males,	Patients were randomly assigned to the intravenous and oral rifampicin treatment groups. In addition to rifampicin, all patients received isoniazid (10 mg/kg per day) and ethambutol (25 mg/kg per day) as a daily infusion	Chronic IV or oral treatment. For the oral trial, patients fasted overnight prior to and 2 h after the ingestion of 600 mg rifampicin. For the IV administration 600 mg RIF were injected over 1 hour	Top-down analysis of IV PK to determine CYP3A4 and CES2 V_{max}<br>Oral dose to check PBPK model for self-induction of CYP3A4	Loos et al. [5]
3	USA	12 Caucasian pediatric patients aged 3 months to 12.8 years, 7 boys and 5 girls.	Patients received 30min infusion of rifampicin every 12h or 8h in some subjects. Doses ranged from 74-450 mg/m2	Chronic PK profiles provided in children (average and dose corrected to 300 mg/m2) and individual 1-compartment PK parameters	Model validation in children	Koup et al. [38]
4	USA	20 Caucasian pediatric patients 3 months to 2.9 years for 19 of the patients and a 14 year old girl. 11 boys and 9 girls	IV doses of average 287 mg/m2 were compared with average oral doses of 324 mg/m2. IV administration over 30 min infusion, Rifadin capsules were dispersed in a USP syrup. Feeding was delayed 1h after administration	Determination of average IV and oral PK profiles normalized to 300 mg/m2 dose	Model validation in children	Koup et al. [39]
5	USA	16 adult healthy volunteers, mean age was 39.1 years, and the mean weight was 79.3 kg,	Overnight fast and 4 hours fast into the study. The high fat meal period provided 792 cal and 57% fat. For the	Average PK profiles for 600 mg RIF in the	Model validation	Peloquin et al. [40]

			antacid part of the meal, HV received 30 mL of aluminum/magnesium hydroxide (Mylanta) 9 h before dosing, at the time of dose, after meals, and at bedtime postdose. Subjects received single oral doses of RIF, 600 mg. They also received 300 mg INH, 30 mg/kg PZA (median, 2,386 mg), and 25 mg/kg EMB (median, 1,950 mg).	fasted, antacid and fed state		
6	USA	38 infants and children (19 of each sex) from 6 to 58 months old (mean 26 months), weight range 6.6 to 17.1 kg (mean 12.4 kg), and height from 60 to 102 cm (mean 85 cm). The average body surface area was 0.53 sq m.	A single dose of rifampin was given orally as a suspension at 10 mg/kg of body weight after a ten- to 12-hour fast. 30mL of 85% sucrose suspension was used as the vehicle	Concentration time profile for RIF	Model validation in children	McCracken et al. [41]
7	South Africa	33 children, mean age 4.05 years, 48% males, with active TB, BW 13.97 kg, height 91.53 cm, 15.71 kg/m2 BMI	each tablet used during the intensive treatment phase contained 60 mg RIF, 30 mg INH and 150 mg PYR (Rimcure®). Children were dosed at 8 mg RIF/kg	Blood sampling at 45 minutes, 1.5, 3.0, 4.0 and 6.0 hours after dosing	Model validation in children	Schaaf et al. [42]
8	NR	NR, assumed a 30 year old, 70 kg BW representative subject	Single ascending doses of RIF in adult healthy volunteers	Blood sampling at 0.5 minutes, 1, 2, 3, 4, 6, 8 and 12h after dosing	Model validation	Acocella et al. [43]
9	Germany	27 children with pulmonary TB between 2 and 14 years. Co-variates other than age not reported.	Single administration of 10 mg/kg RIF as a tablet in the fasted state.	Blood sampling at 1, 2, 3, 4, 5, 7 and 24 h after dosing. Average profiles by 3 age groups 2–<6 years (n = 7), 6–<10 years (n = 11), and 10–14 years (n = 9)	Model validation in children	Thee et al. [44]
10	South Africa	20 TB patients, 1.09 (0.49) year old, 45% of females.	Administration of Rimcure® FDC (30 mg INH + 60mg RIF + 150 mg PZA) to target 10 mg/kg and 15mg/kg RIF. Tablets were crushed and administered in 2-5mL water suspension	4 datapoint Plasma profiles up to 5 hours of RIF for 15mg/kg	Model validation in children	Thee et al. [31]

11	USA	8 pediatric patients receiving shunt placement (1day to 18 years)	A single intravenous dose of 20 mg/kg administered over 60 min. Plasma was collected at times 0, 0.5, 1, 2, 4, 5 and 8 h post infusion start.	Individual concentration in plasma at a given time post infusion. The clearance and volume of distribution are reported	Model validation in children	Nahata et al. [45]
12	India	Healthy volunteer populations (n=13-22)	6 PK studies with corresponding dissolution of the FDC at pH 1, 2 and 6.8 at 30 rpm to 100 rpm were reported for RIF. 450 mg or 600 mg RIF doses were used for the study. All volunteers were fasted and the drug was dosed with 200 mL water.	C_{max}, t_{max} and AUC and PK profiles for various FDC and their loose RLD comparators. Sampling times 36-24h but only concentrations up to 8h reported	PBBM validation	Agrawal et al. [46] [47]
13	Brazil	28 healthy volunteers, randomized 2-period crossover bioequivalence study Washout of 1 week, 46% males, 37 years, 68.44 kg, 1.67 m height.	-10H to +4H Fasted crossover administration of 150mg INH+300 mg RIF. Test = FIOCRUZ/RJ batch 09060664. Ref = Rifinah® Sanofi Batch A9362 with 200 mL water. No water until +2H	Oral individual PK profiles for INH and RIF + subjects' covariates and in vitro dissolution for dosage forms	PBBM validation Examination of individual PK profiles and understanding risks related to physiological variables	STPH71/10

RESULTS

ADULT PK PROFILES FOR INH

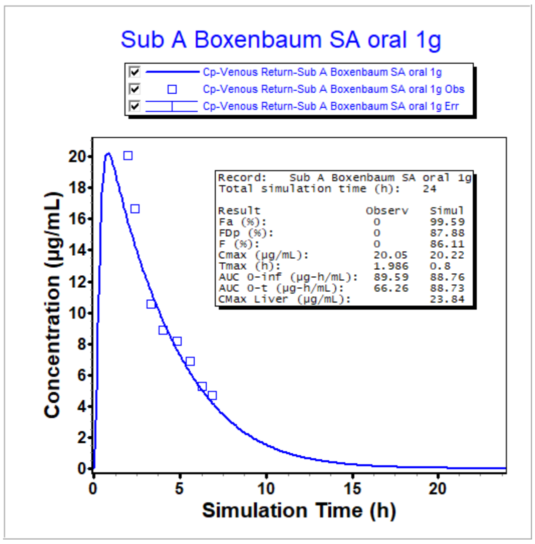

Figure S12 **Prediction of INH PK profile following administration of 1 g INH to a SA adult. Data from Boxenbaum, & Riegelman [24]**

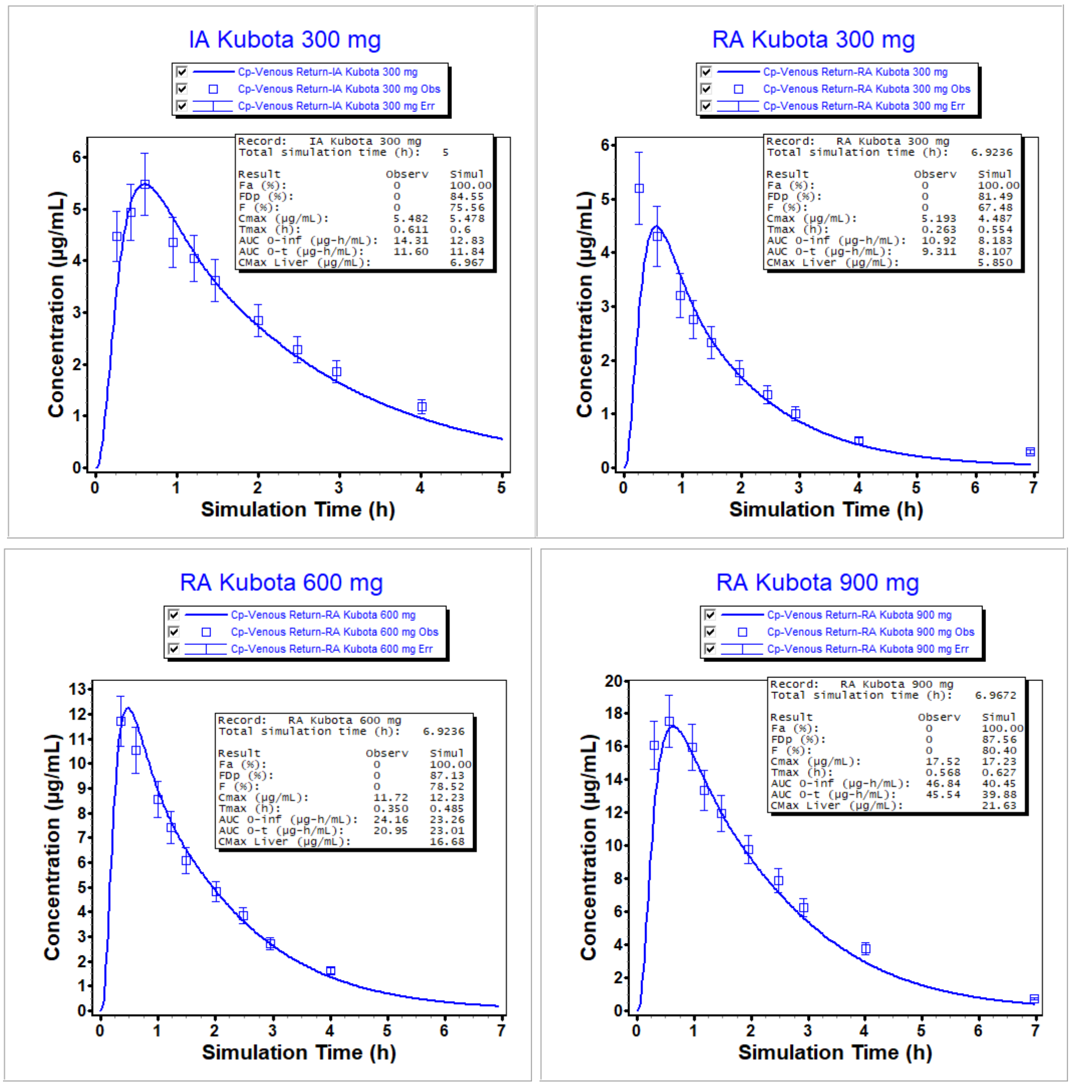

Figure S13 **Prediction of INH PK profile following administration of 300 mg INH to IA and 300 mg, 600mg and 900 mg to RA population. Data from Kubota et al. [25]**

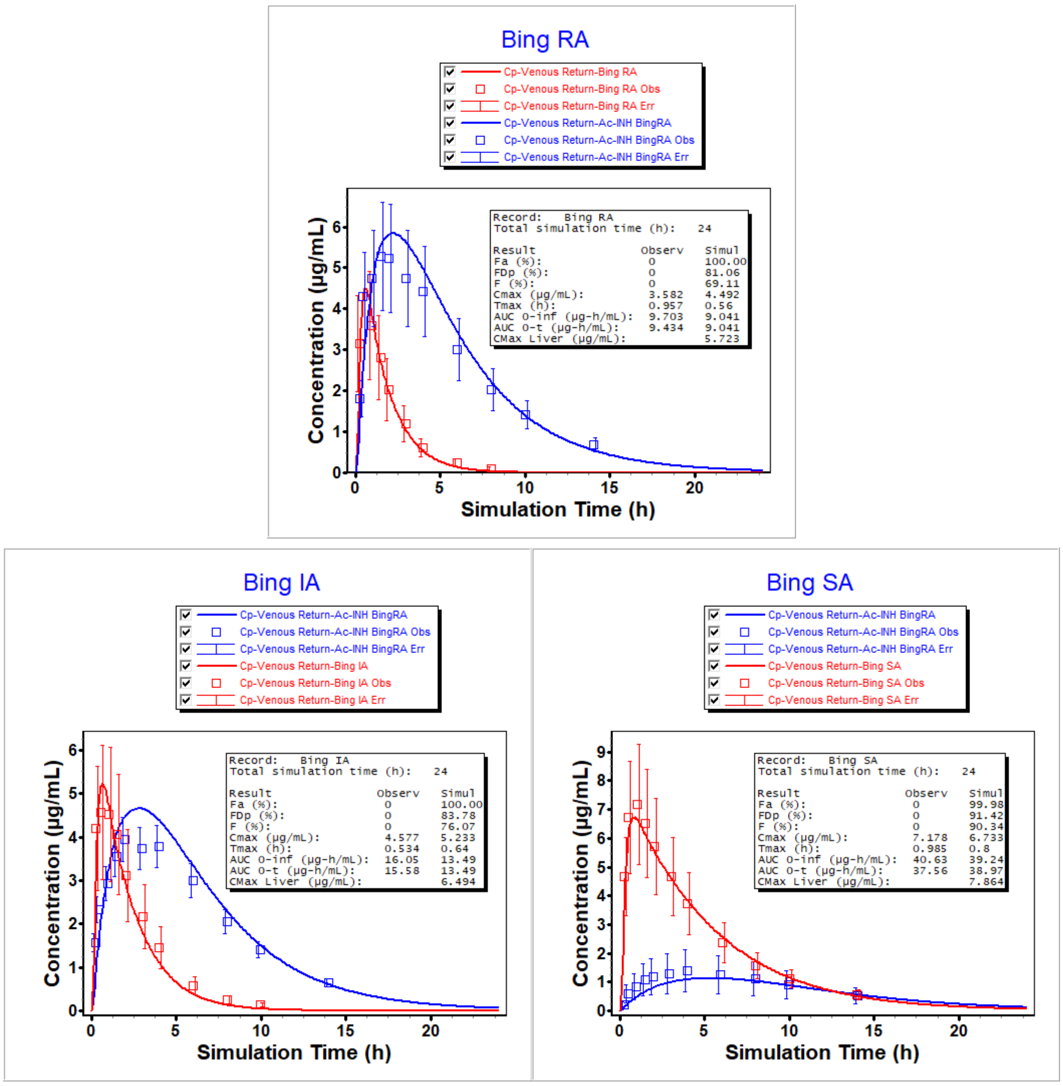

Figure S14 **Prediction of INH and Ac-INH PK profiles following administration of 300 mg INH to RA, IA and SA populations. Data from Bing et al. [15].**

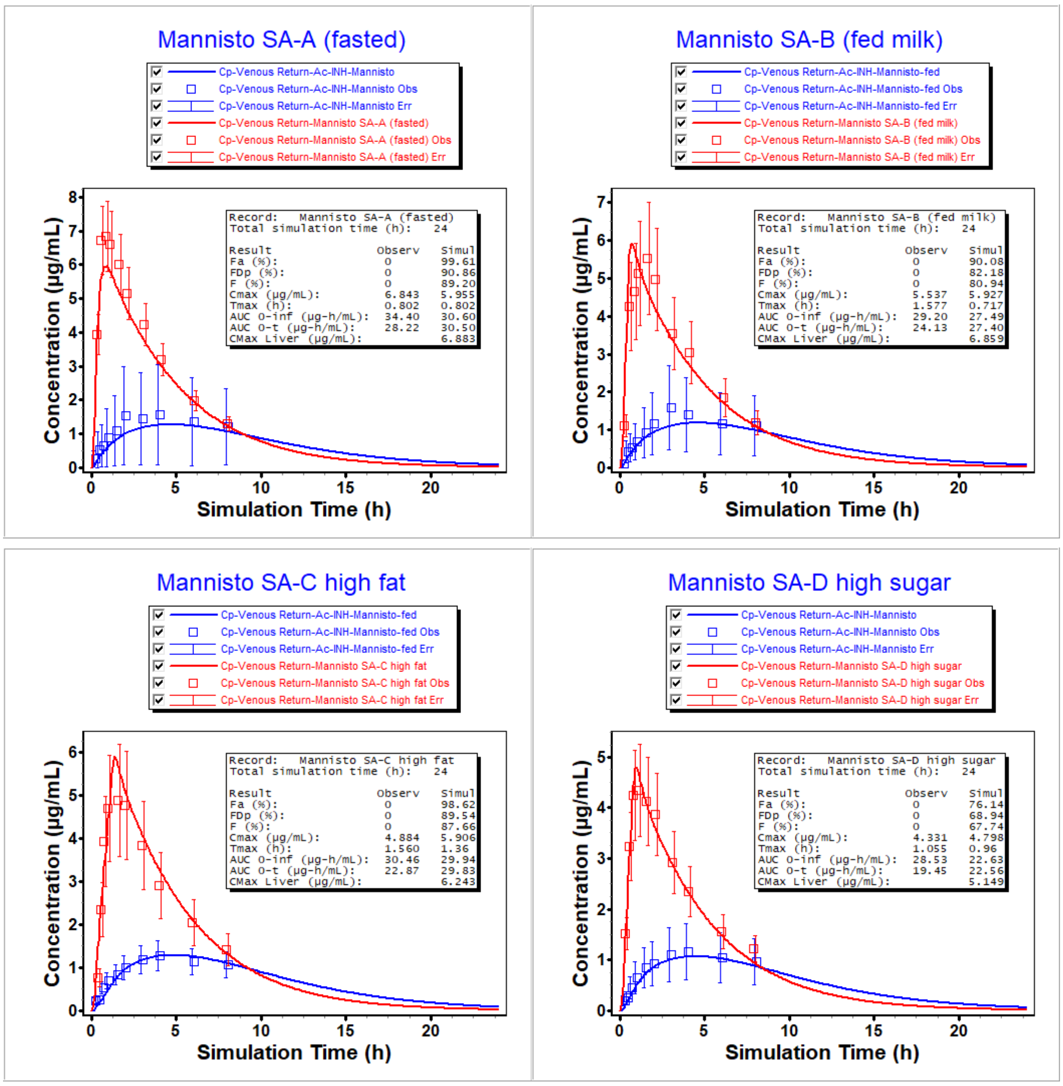

Figure S15 **Prediction of INH and Ac-INH PK profiles following administration of 300 mg INH to SA populations in the fasted state and following different meal types. Data from Männisto et al. [26].**

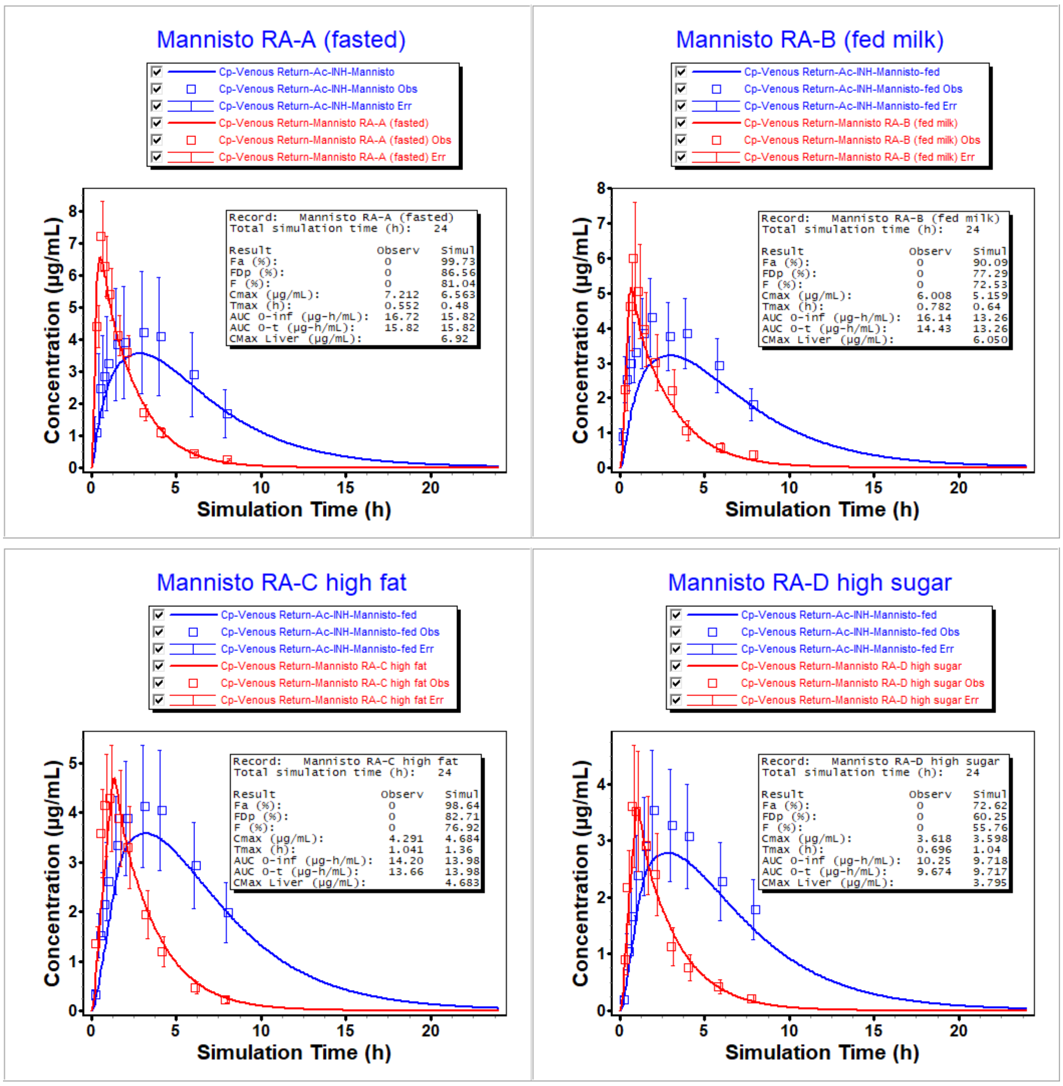

Figure S16 **Prediction of INH and Ac-INH PK profiles following administration of 300 mg INH to RA populations in the fasted state and following different meal types. Data from Männisto et al. [26].**

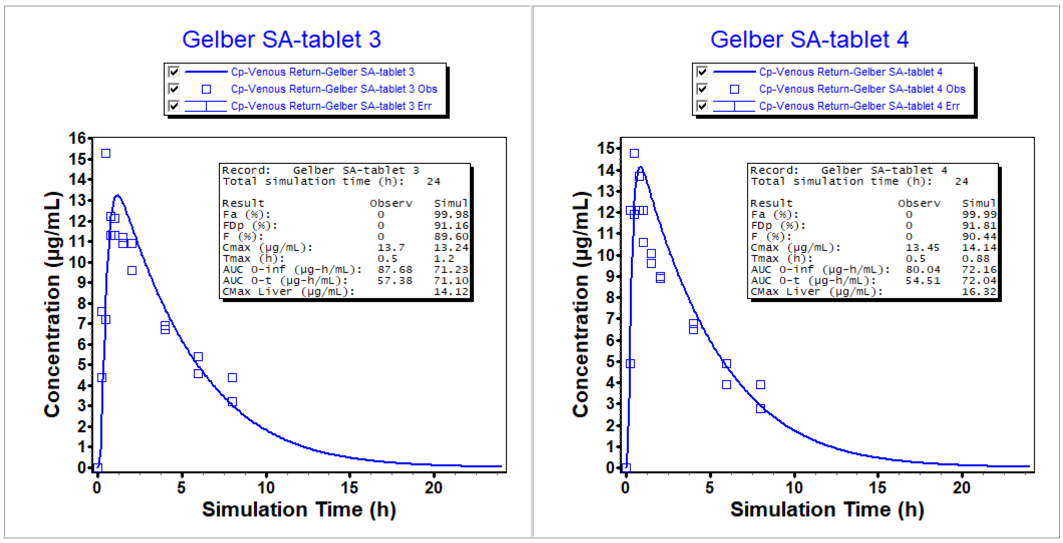

Figure S17 Prediction of INH PK profiles following administration of 10 mg/kg INH to SA populations using a slow dissolving formulation (tablet 3) and rapid dissolving formulation (tablet 4). Data from Gelber et al. [30].

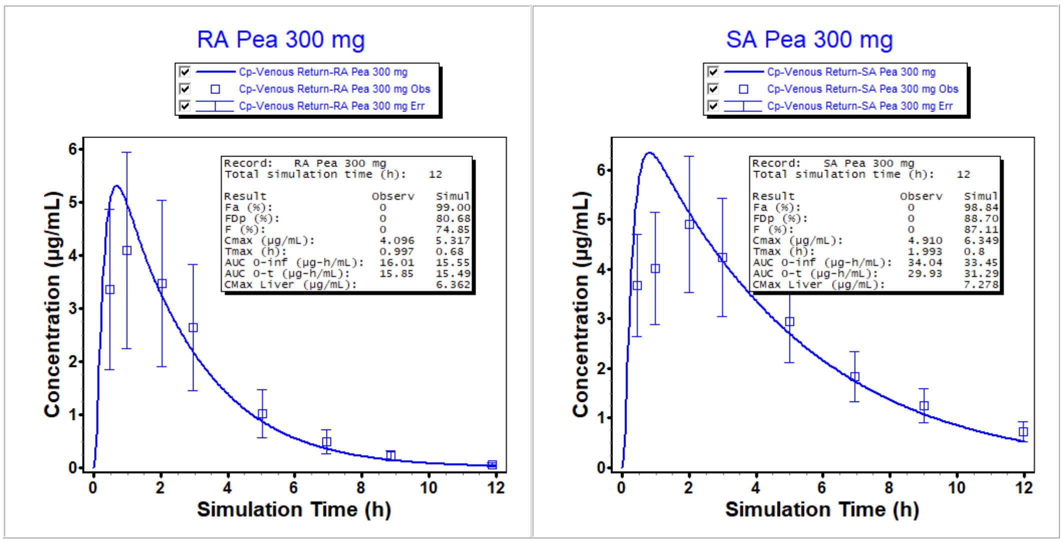

Figure S18 Prediction of INH PK profiles following administration of 300 mg INH to RA and SA populations. Data from Pea et al. [27].

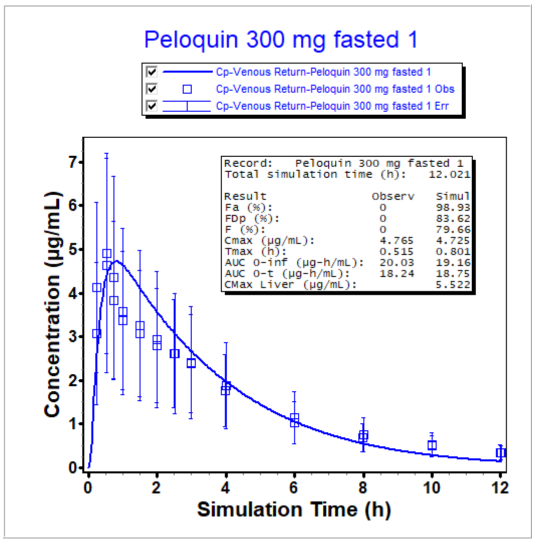

Figure S19 Prediction of INH PK profiles following administration of 300 mg INH to SA populations. Data from Peloquin et al. [28].

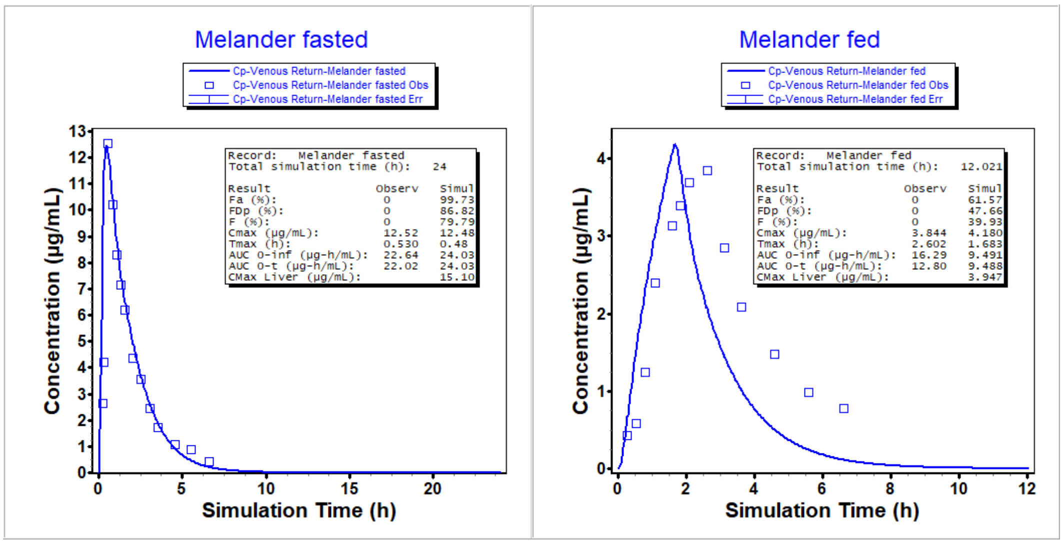

Figure S20 Prediction of INH PK profiles following administration of 10 mg/kg INH to subject 2 in fasted and following a high carbohydrate meal. Data from Melander et al. [29].

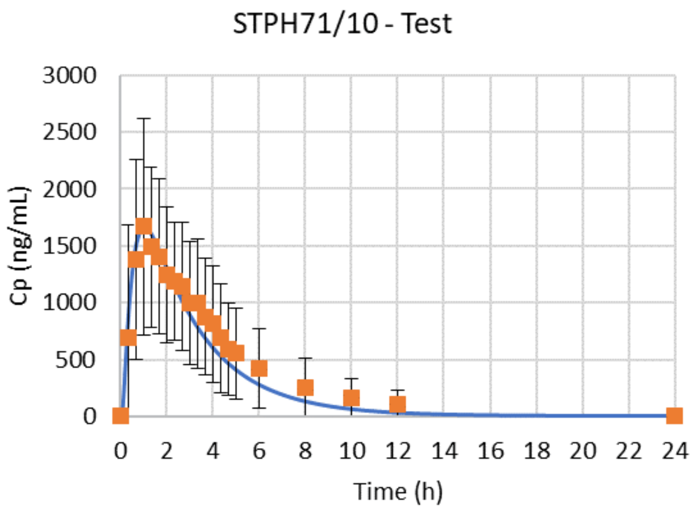

Figure S21 **Prediction of INH PK profiles following administration of 150 mg INH during study STPH71/10. Test formulation = FIOCRUZ/RJ batch 09060664.**

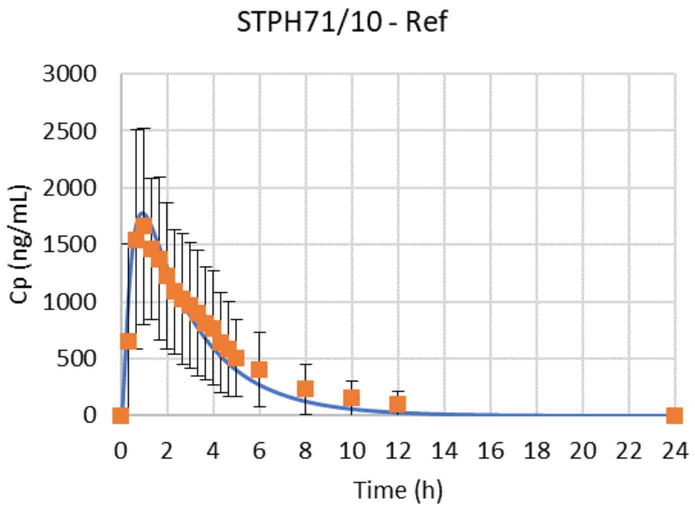

Figure S22 **Prediction of INH PK profiles following administration of 150 mg INH during study STPH71/10. Ref formulation= Rifinah® Sanofi Batch A9362**

PEDIATRIC PK PROFILES FOR INH

The combined average PK profile following administration of 5 mg/kg and 10 mg/kg INH to pediatric patients around 1 year of age is shown respectively in **Figure S23** and **Figure S24**.

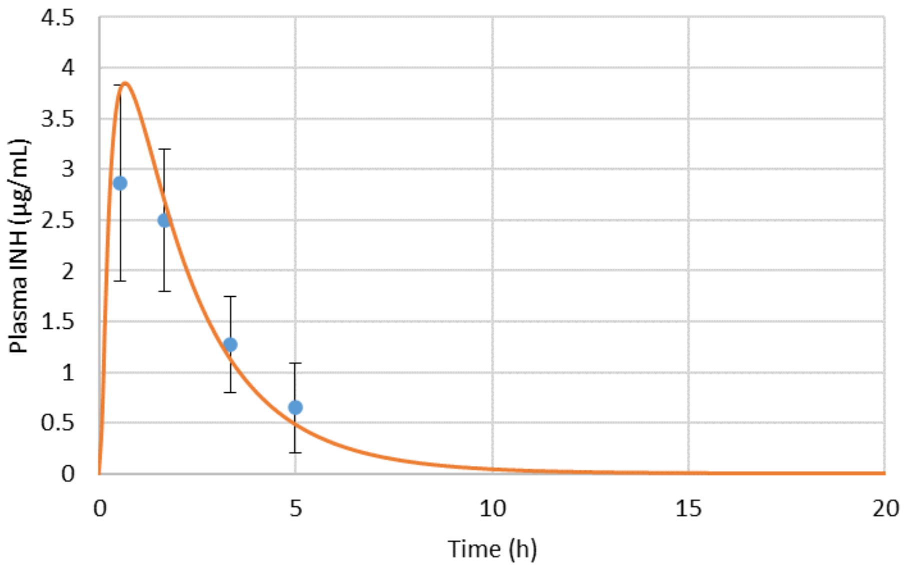

Figure S23 Prediction of average INH PK profile at 5 mg/kg in pediatric patients. Data from Thee et al. [31]

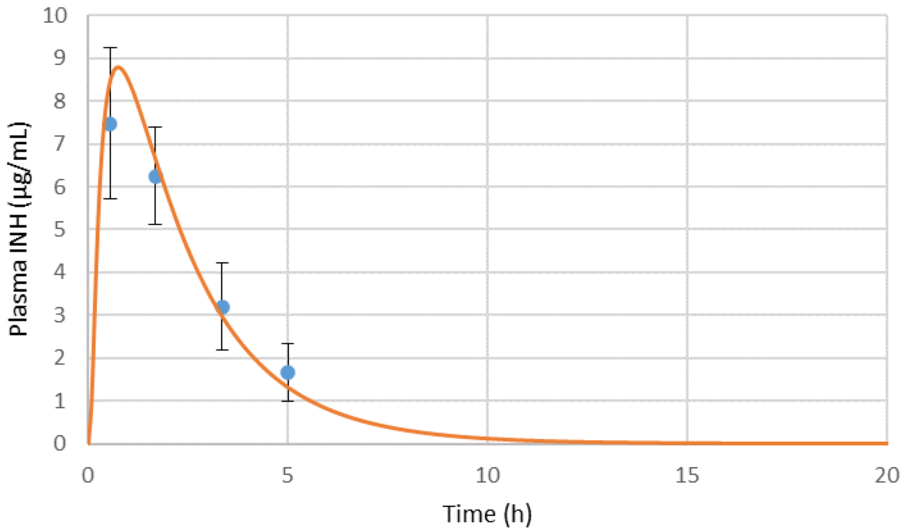

Figure S24 Prediction of average INH PK profile at 10 mg/kg in pediatric patients. Data from Thee et al. [31]

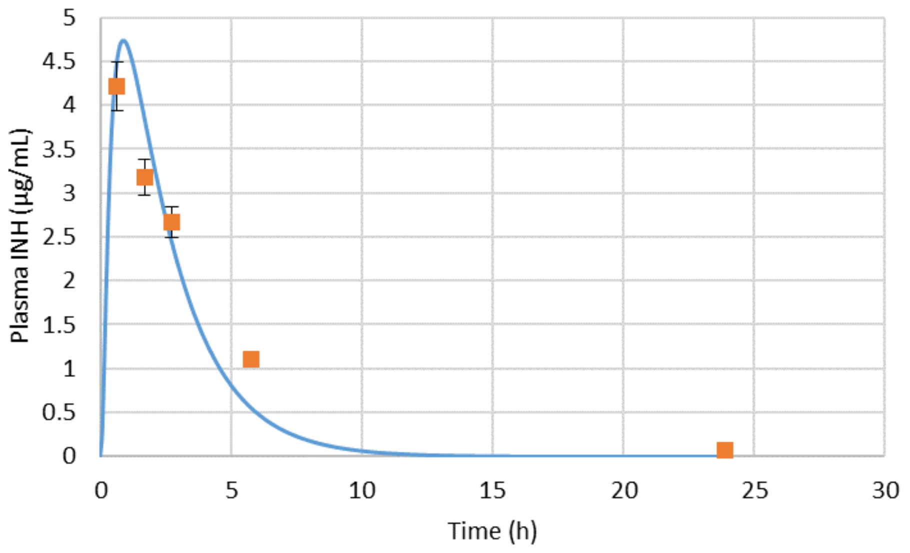

Figure S25 Prediction of average INH PK profile at 5 mg/kg in pediatric patients. Data from Roy et al. [33]

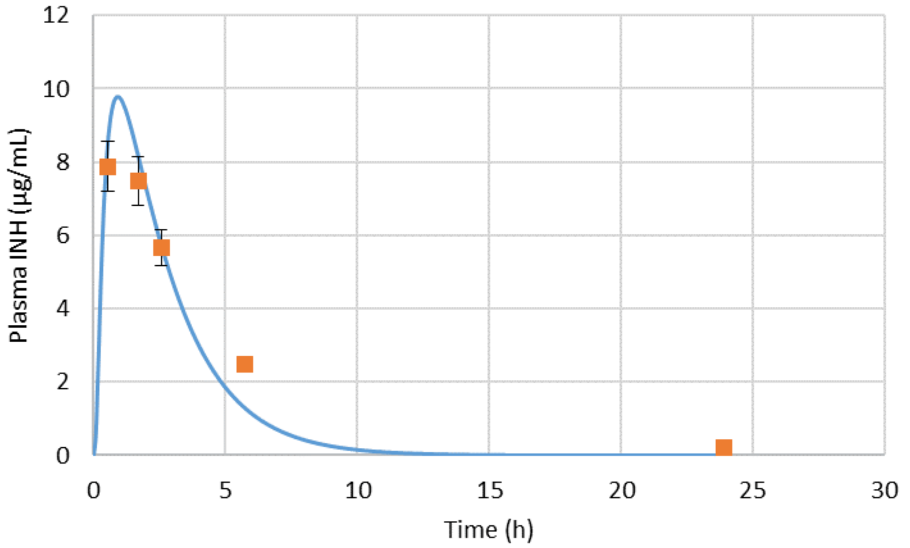

Figure S26 Prediction of average INH PK profile at 10 mg/kg in pediatric patients. Data from Roy et al. [33]

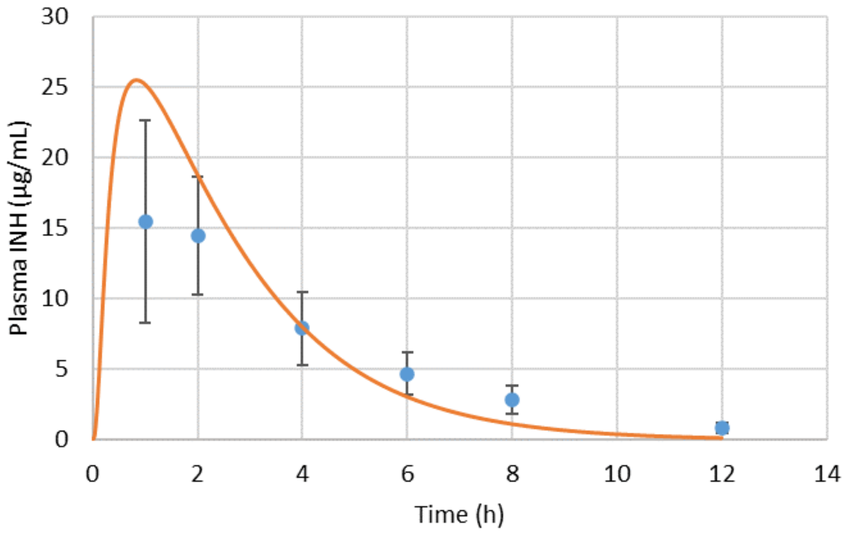

Figure S27 **Prediction of INH plasma profile at 25 mg/kg in pediatric patients. Data from Phaisal et al. [32]**

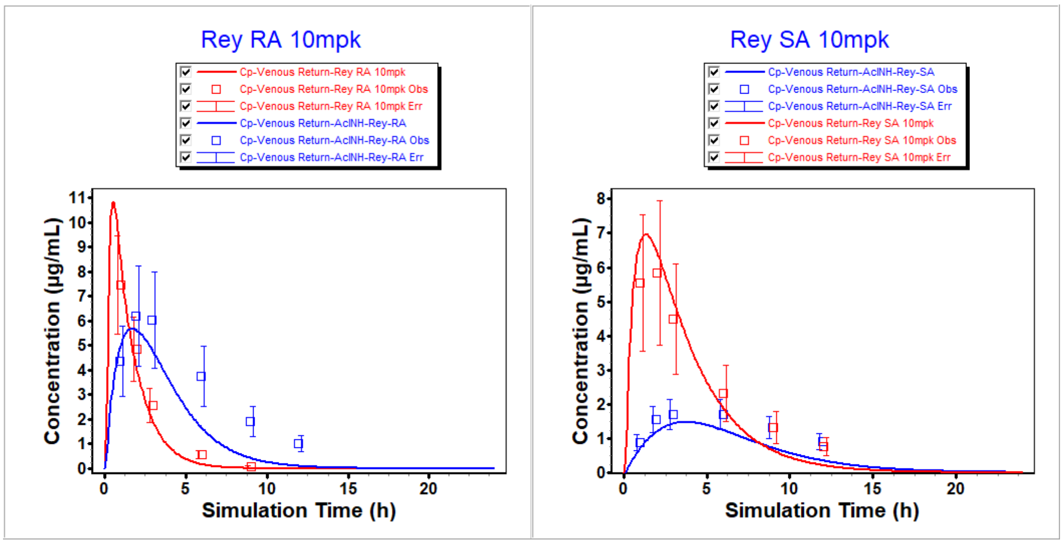

Figure S28 **Prediction of INH and Ac-INH plasma profile at 10 mg/kg in RA and SA pediatric patients. Data from Rey et al. [34]**

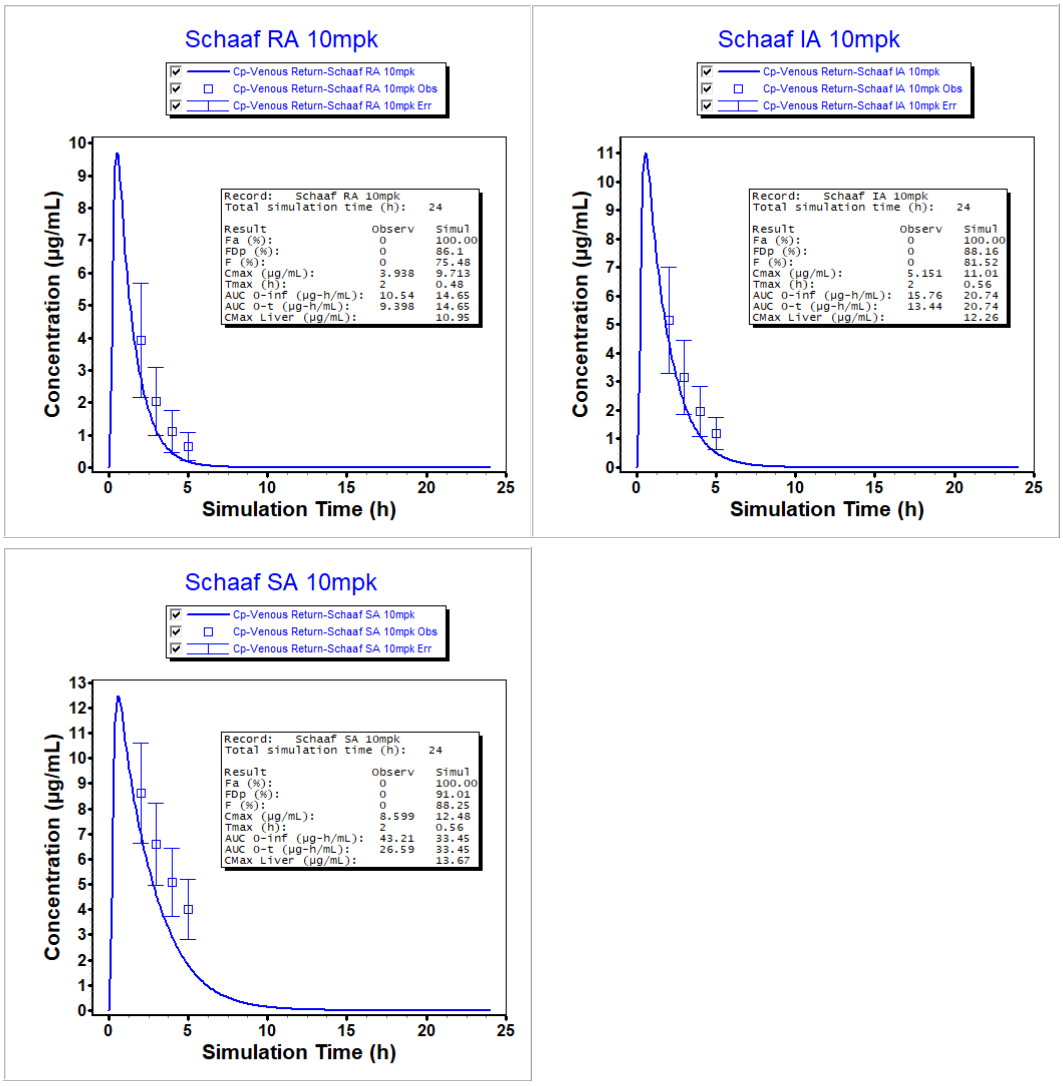

Figure S29 **Prediction of INH plasma profile at 10 mg/kg in RA, IA, and SA pediatric patients. Data from Schaaf et al. [35]**

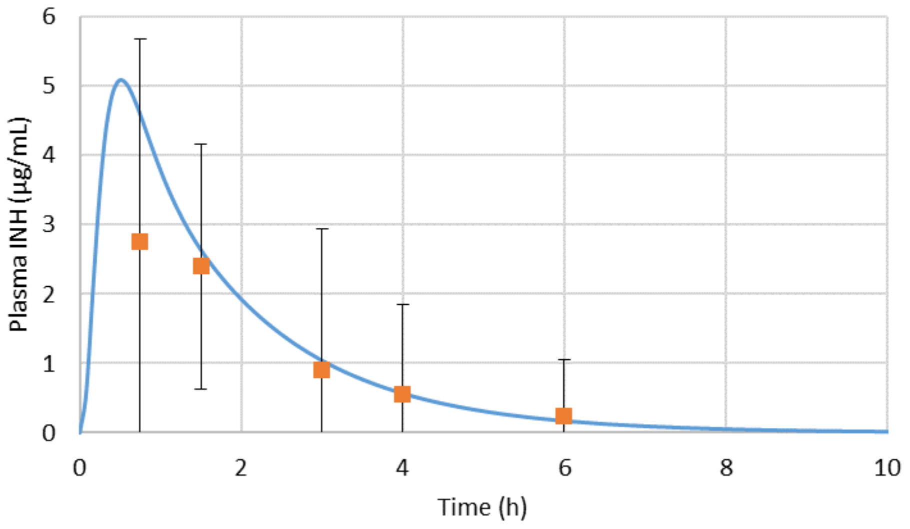

Figure S30 **Prediction of INH plasma profile at 5 mg/kg in pediatric patients. Data from McIlleron et al. [36]**

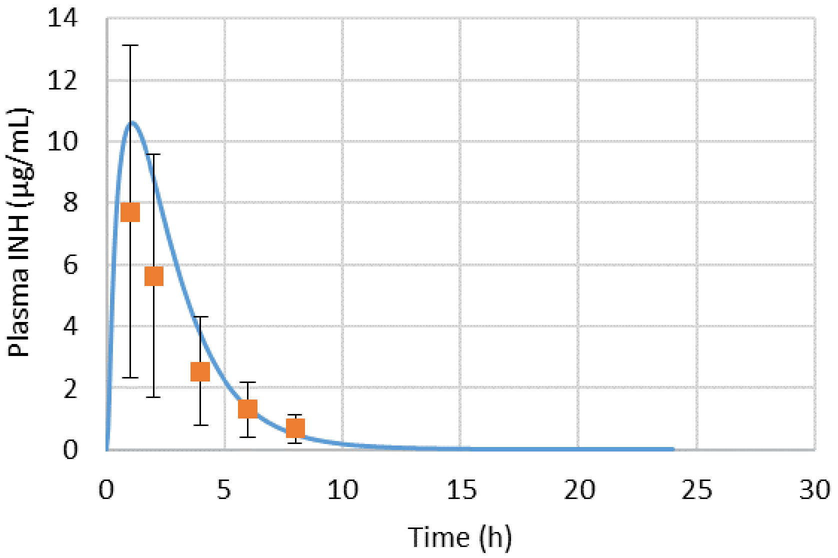

Figure S31 **Prediction of INH plasma profile at 14 mg/kg in pediatric patients. Data from Bekker et al. [37]**

ADULT PK PROFILES FOR RIF

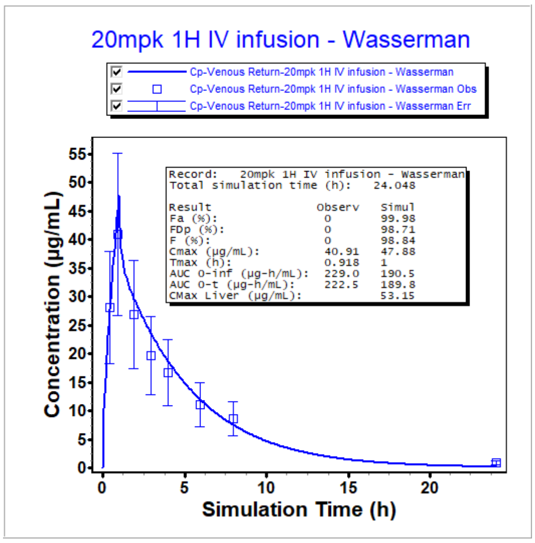

Figure S32 Prediction of RIF plasma profile at 20 mg/kg following 1 hour infusion in pulmonary TB patients. Data from Wasserman et al. [17].

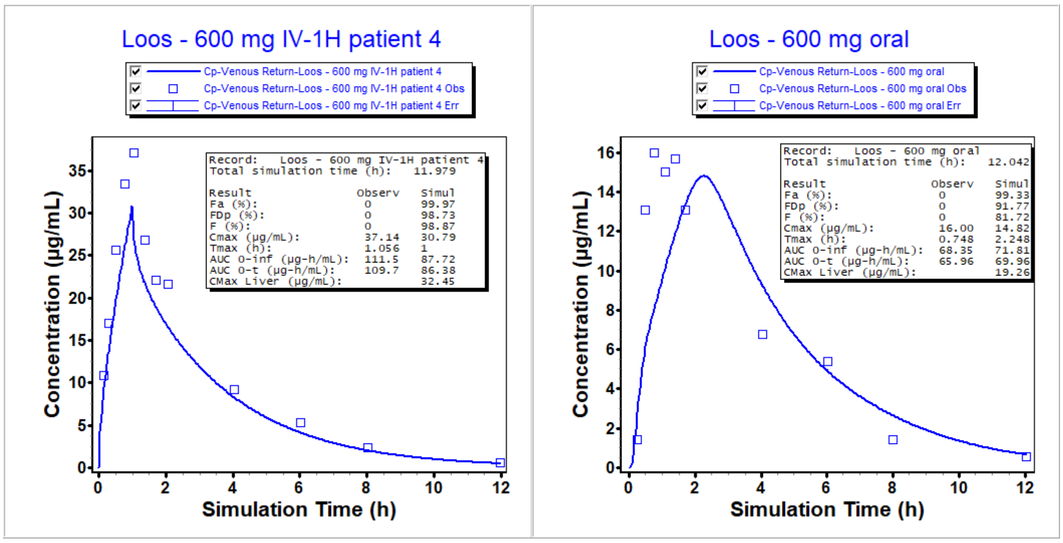

Figure S33 Prediction of RIF plasma profile at 600 mg following 1 hour infusion or oral administration. Data from Loos et al. [5].

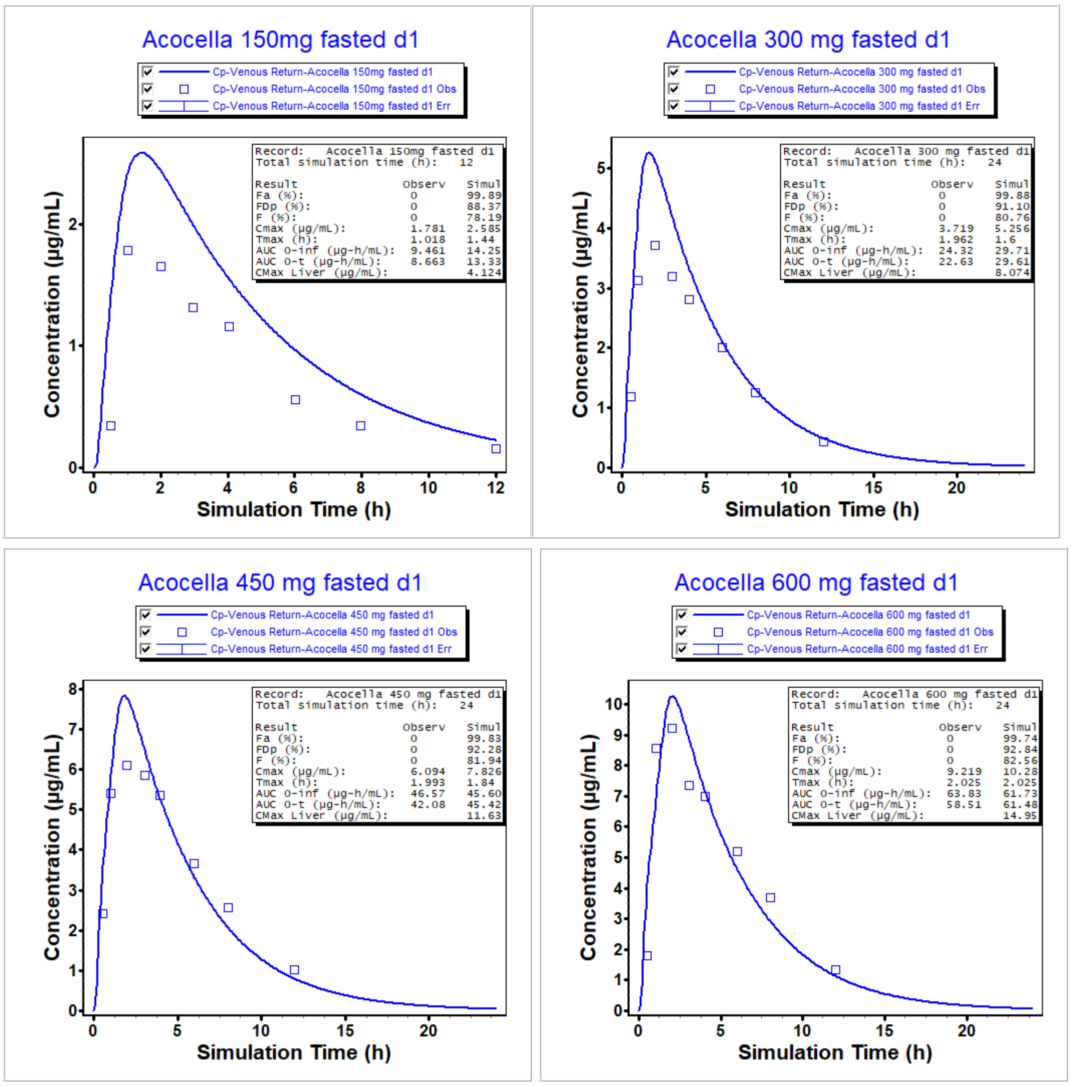

Figure S34 Prediction of RIF plasma profile after oral administration to healthy volunteers from 150 mg to 600 mg. Data from Acocella et al. [43].

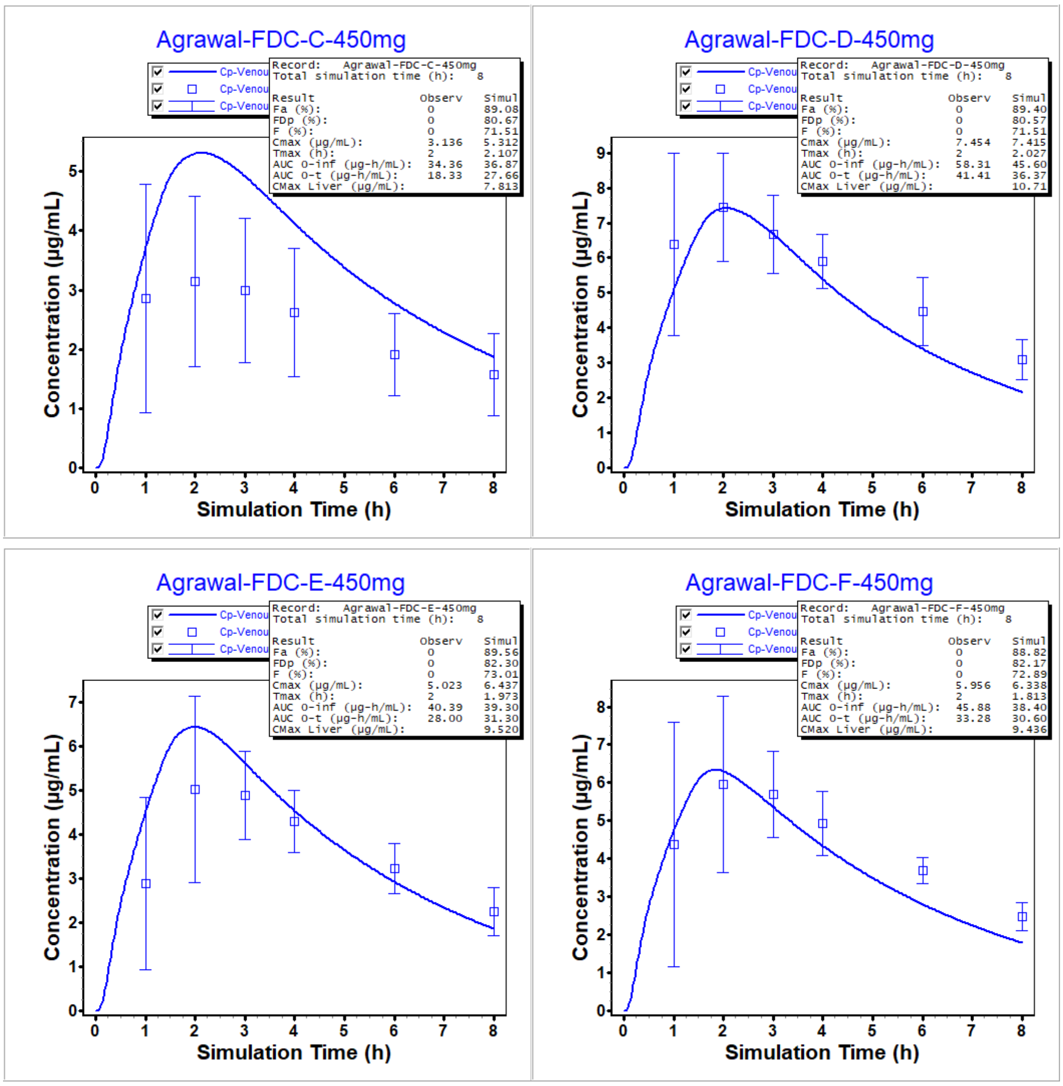

Figure S35 Prediction of RIF plasma profile after oral administration to healthy volunteers of 450 mg formulations C, D, E and F. Data from Agrawal et al. [46] [47]

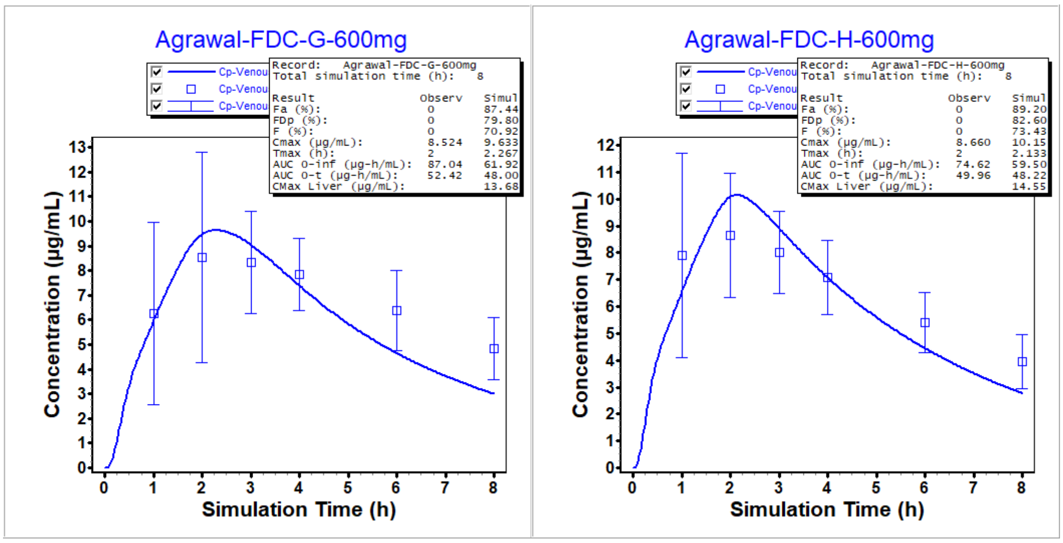

Figure S36 Prediction of RIF plasma profile after oral administration to healthy volunteers of 600 mg formulations G and H. Data from Agrawal et al. [46] [47]

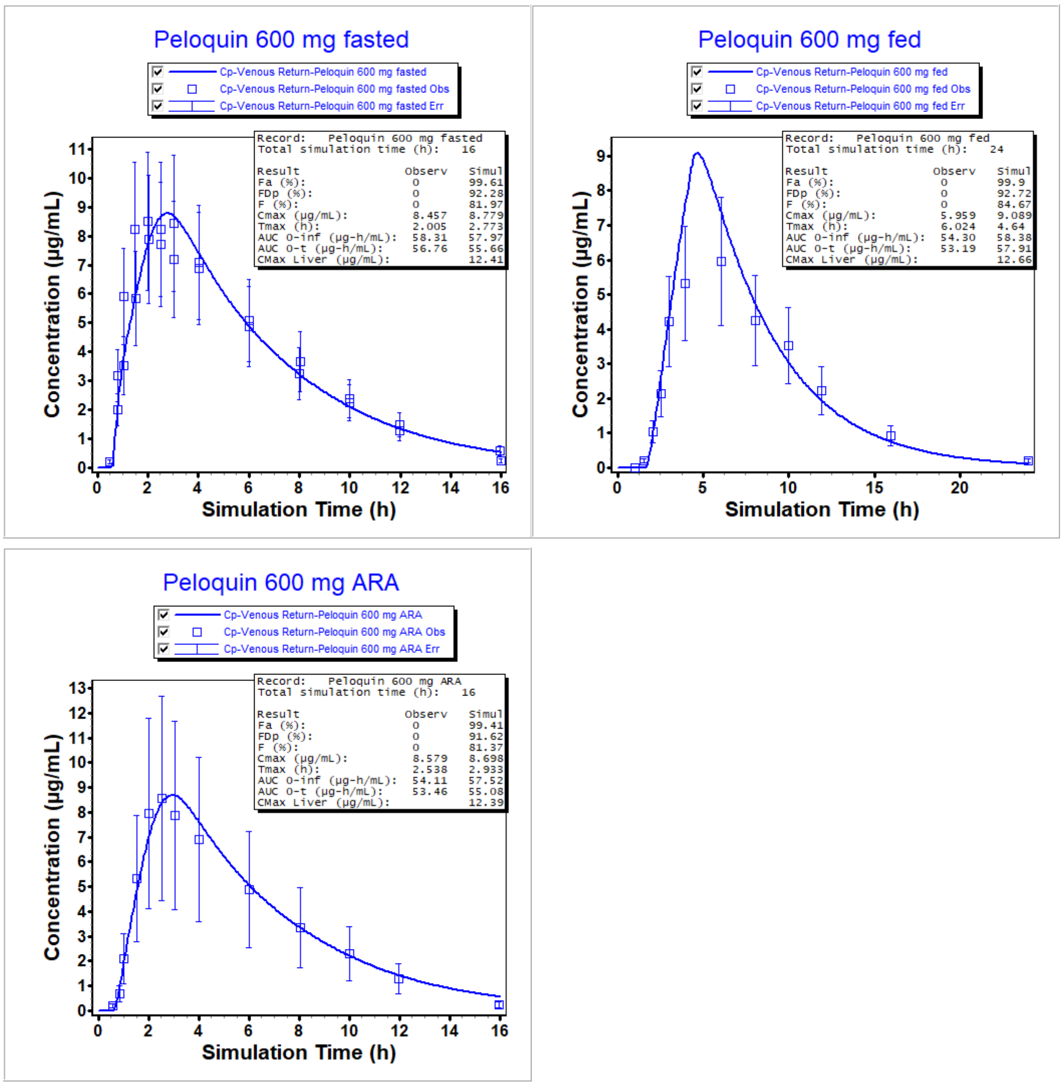

Figure S37 Prediction of RIF plasma profile after oral administration to healthy volunteers of 600 mg formulations in the fasted state, following high fat meal and acid reducing agent treatement. Data from Peloquin et al. [40]

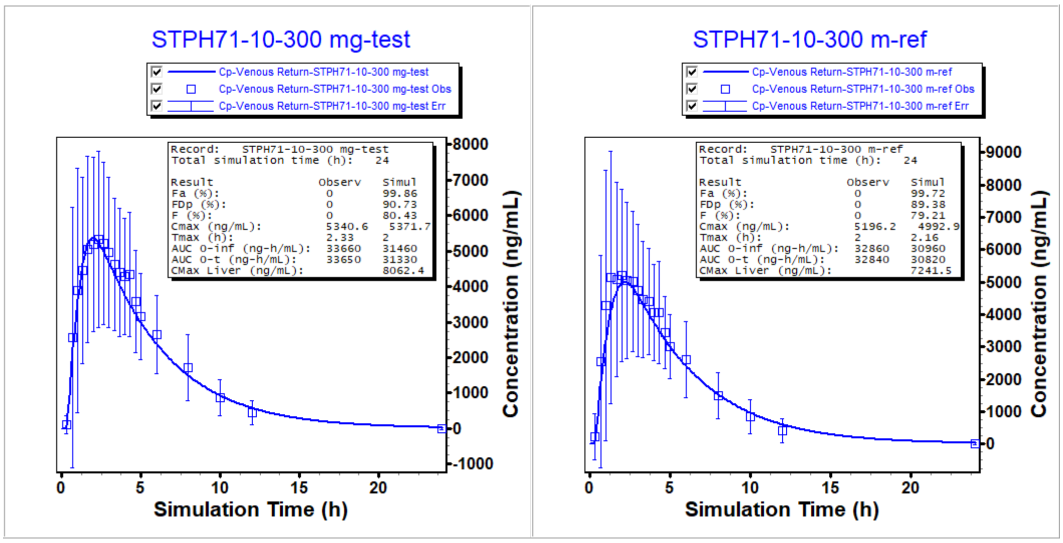

Figure S38 Prediction of RIF PK profiles following administration of 300 mg RIF during study STPH71/10. Test formulation = FIOCRUZ/RJ batch 09060664. Ref formulation= Rifinah® Sanofi Batch A9362

PEDIATRIC PK PROFILES FOR RIF

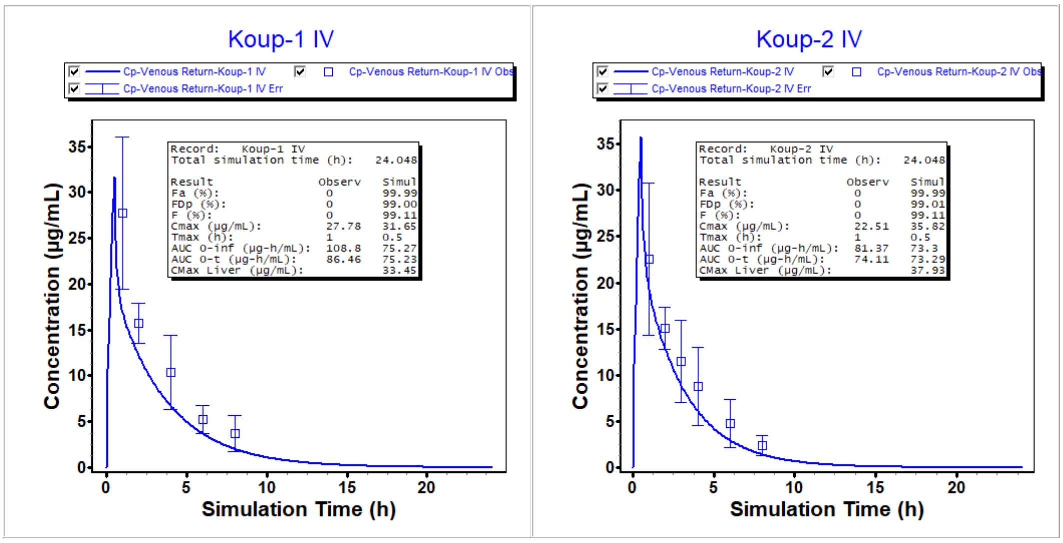

Figure S39 Prediction of RIF PK profiles following IV administration of RIF. Data from Koup et al. [38] and Koup et al. [39].

For all pediatric oral profiles below, the profile is simulated with either 20% volume in the small intestine and 2% in the colon (left panel) or 7.5% volume in the small intestine and 2% in the colon (right panel).

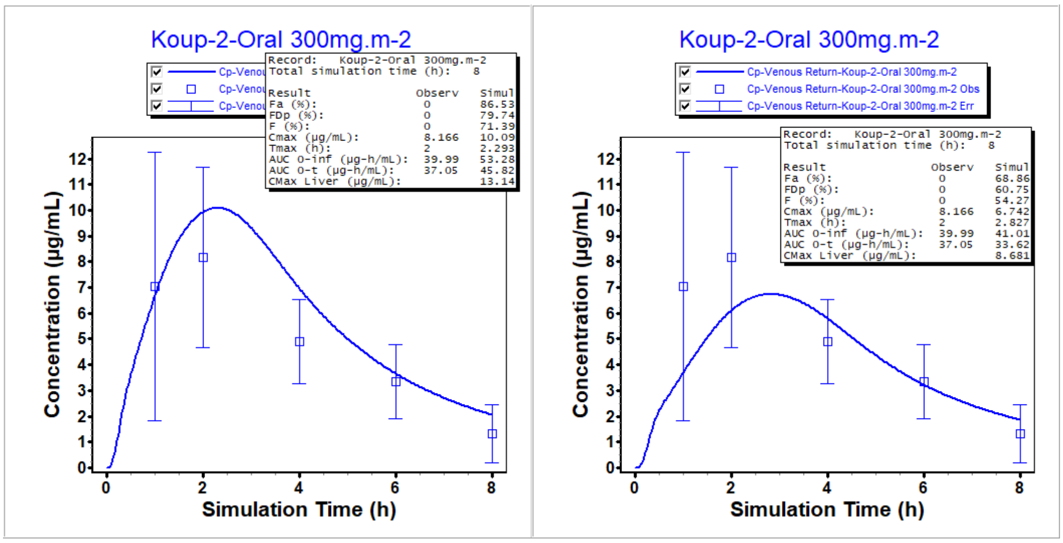

Figure S40 Prediction of RIF PK profiles oral administration of 300 mg/m2 RIF. Data from Koup et al. [39].

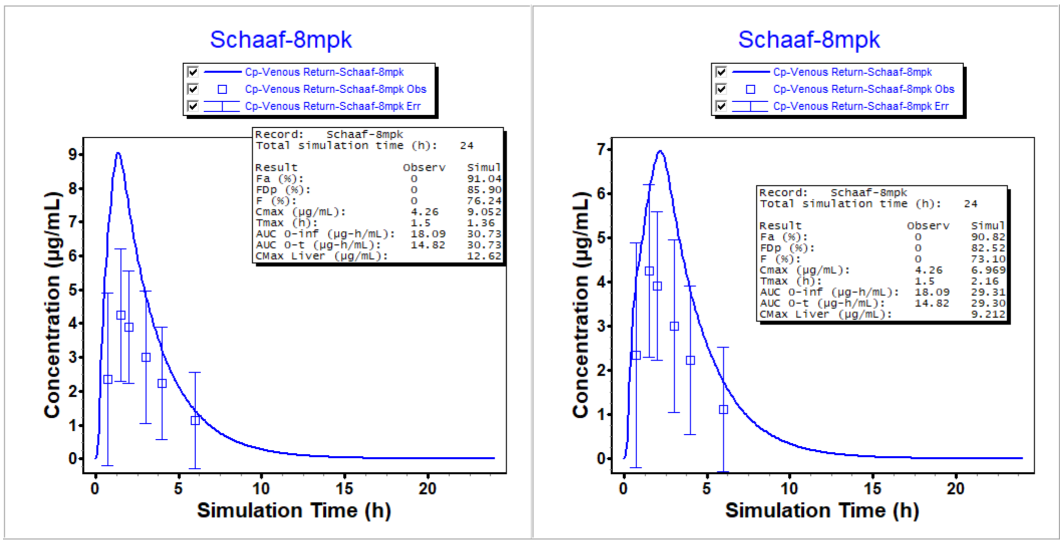

Figure S41 Prediction of RIF PK profiles oral administration of 8 mg/kg RIF. Data from Schaaf et al. [42].

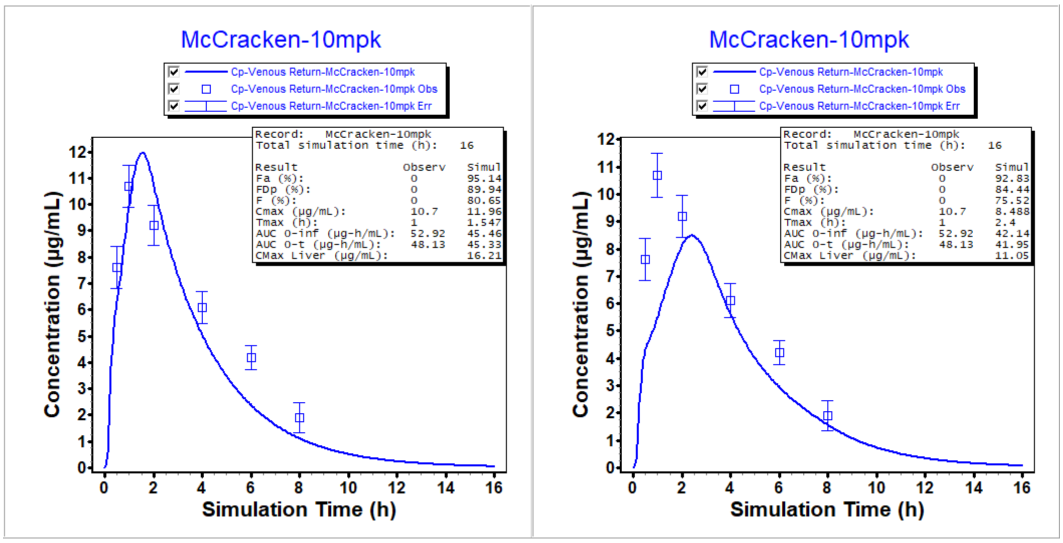

Figure S42 Prediction of RIF PK profiles oral administration of 10 mg/kg RIF. Data from McCracken et al. [41].

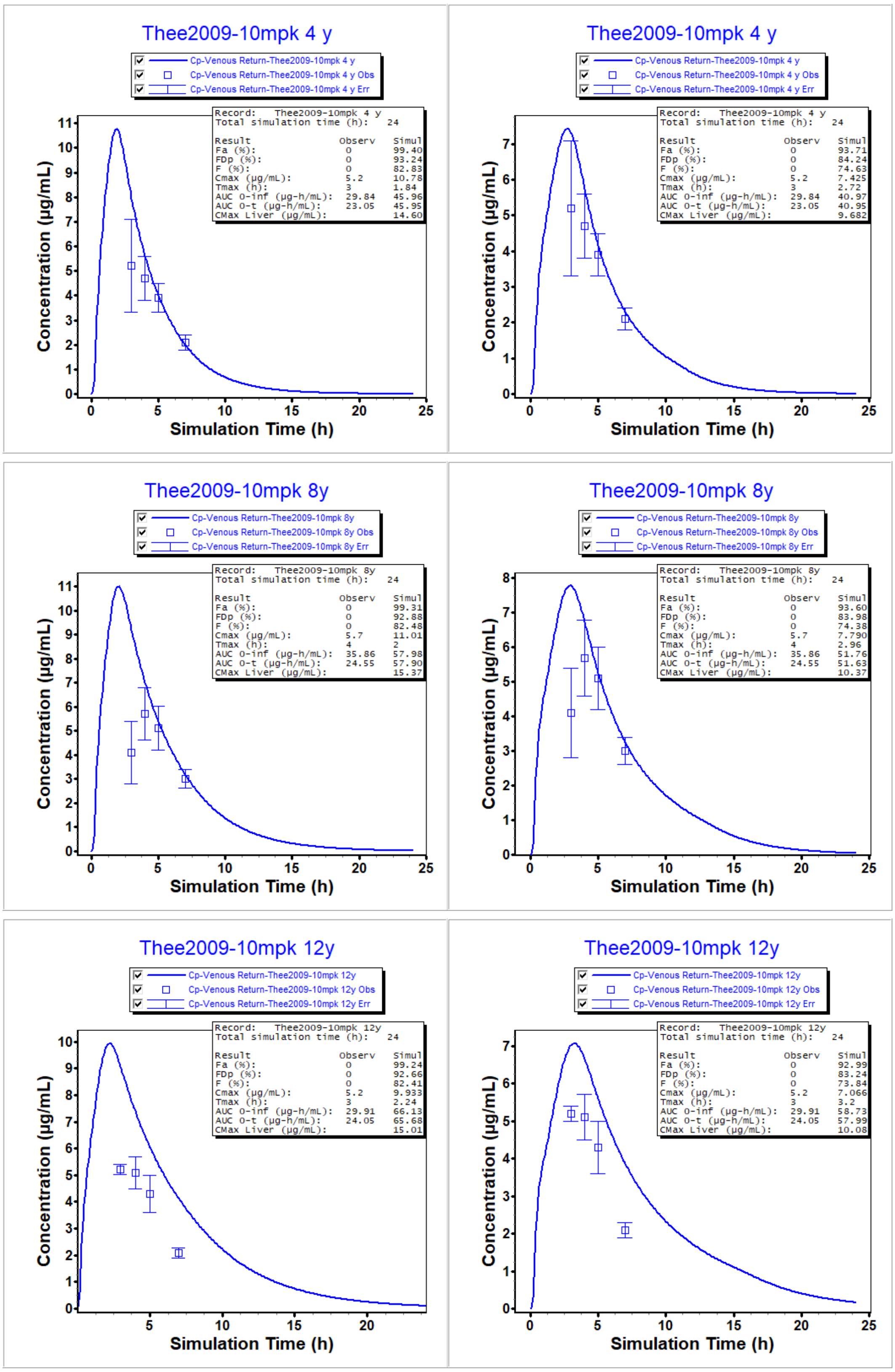

Figure S43 Prediction of RIF PK profiles oral administration of 10 mg/kg RIF in 4 years, 8 years and 12 years old populations. Data from Thee et al. [44].

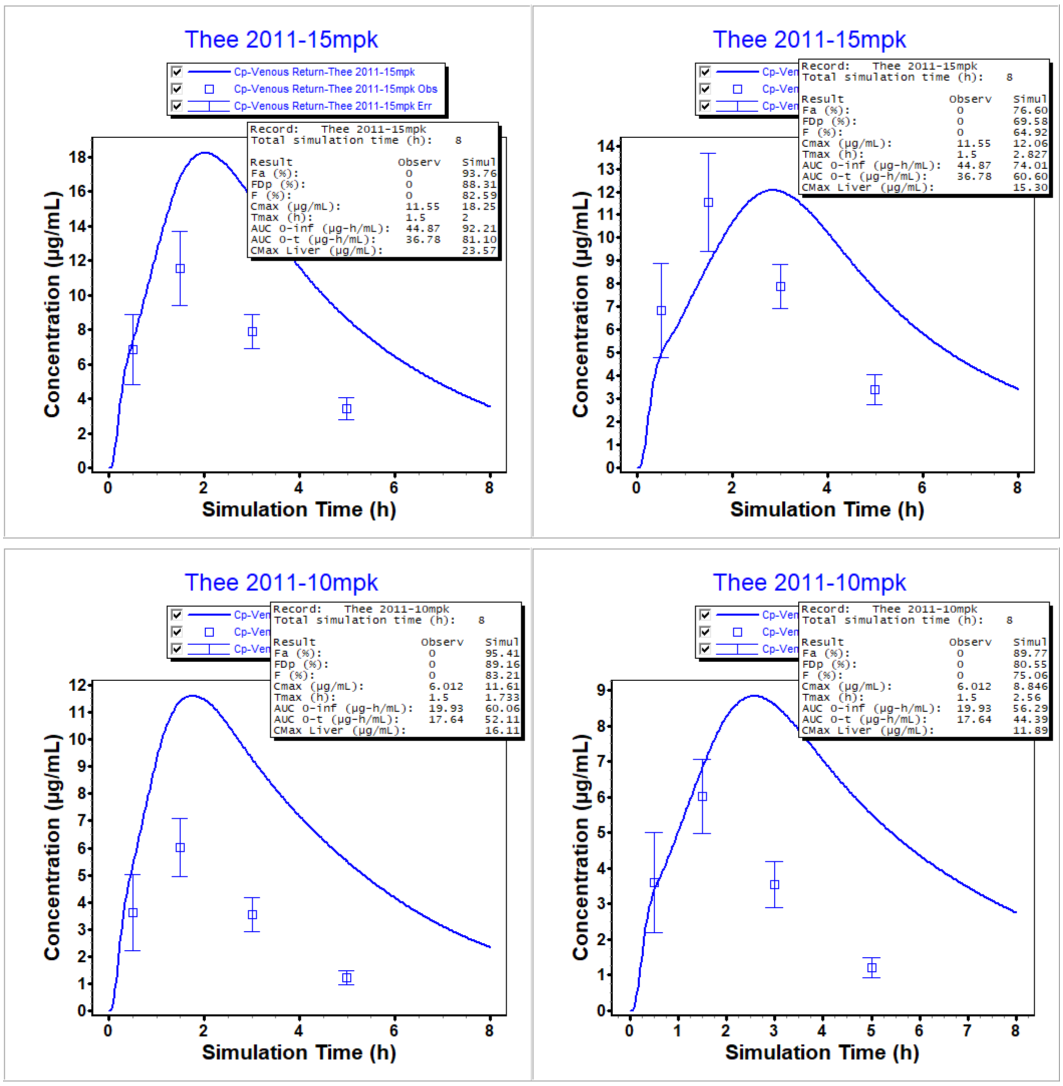

Figure S44 Prediction of RIF PK profiles oral administration of 10 mg/kg RIF in 4 years, 8 years and 12 years old populations. Data from Thee et al. [44].

It is concluded that the 20% volume in the small intestine and 2% volume in the colon are appropriate approximations in children as in adults to predict the volume of water available to handle precipitation and dissolution of RIF.

SENSITIVITY ANALYSES

ISONIAZID

The parameters selected for INH PSA are shown in Table S3. The fasted adult physiology was chosen for this simulation. The dose selected for INH was 300 mg which is the recommended

dose in adult maintenance treatment. The INH PSA results on AUC and C_{max} for the most influential 5 parameters are shown in Figure S45 and Figure S46 respectively.

Table S3 Parameters Chosen for Sensitivity Analysis for INH

Parameter	Baseline Value	Lower Bound	Upper Bound
Particle radius (μm)	25	2.5	250
Dose volume (mL)	250	25	1000
Solubility (mg/mL)	161	16.1	1610
P_{eff} (10^{-4} cm/s)	6.7	3.35	13.4
Volume of the lower intestine (%)	10	5	20
Volume of the upper intestine (%)	40	20	80
Stomach transit time (h)	0.25	0.125	0.5
Small intestinal transit time (h)	3.3	1.65	6.6
Cecum transit time (h)	4.5	2.25	9
Colon transit time (h)	13.5	6.75	27
Stomach pH	1.3	0.5	8
PBPK NAT2 V_{max} (mg/s/mg-enz)	0.00122	0.000609	0.00244
Gut NAT2 V_{max} scale factor	1	0.5	2

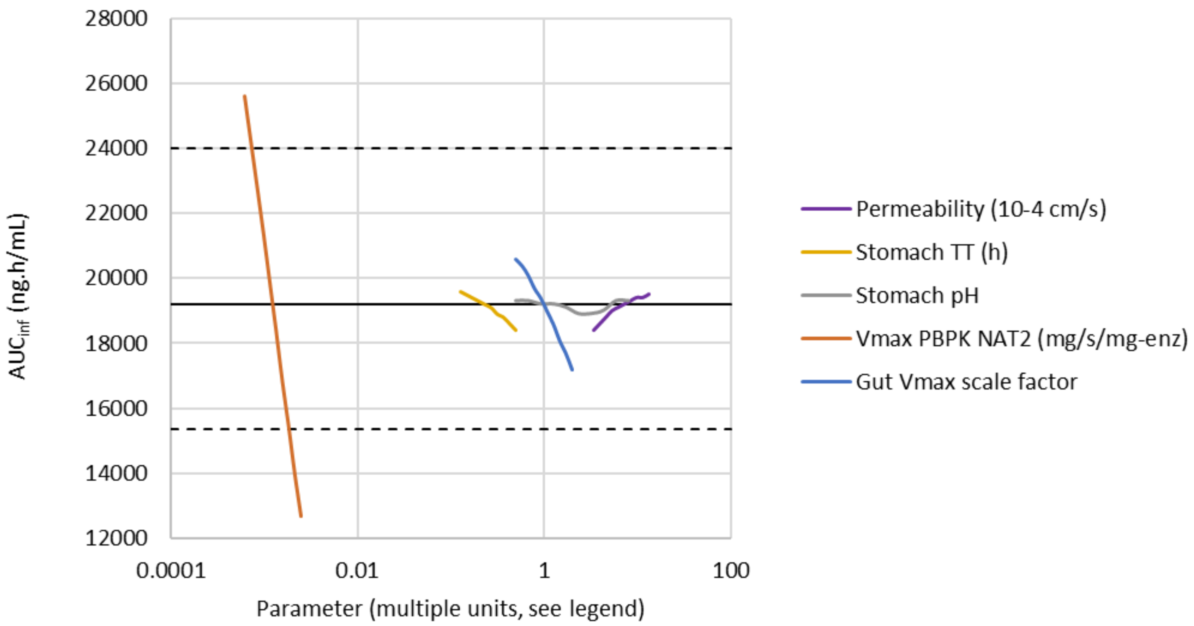

Figure S45 Effect of main 5 parameters on INH AUC. The horizontal solid line shows the baseline simulation and the horizontal dotted lines illustrate the boundaries for bioequivalence.

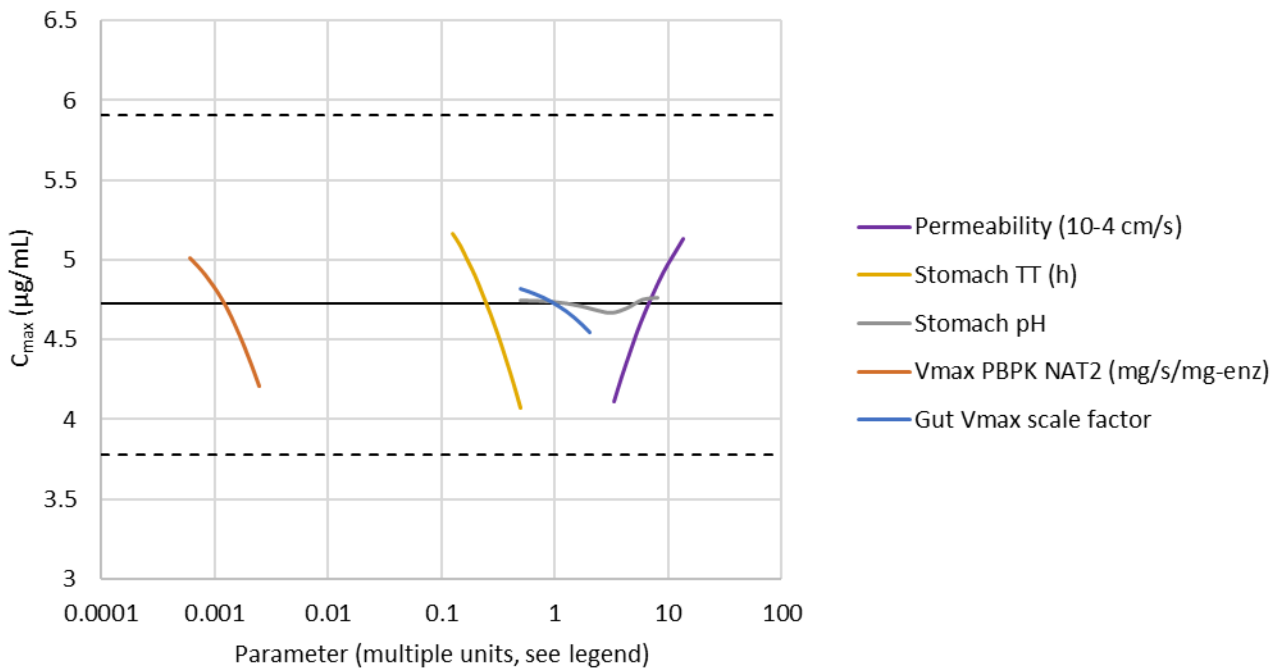

Figure S46 Effect of main 5 parameters on INH C_{max}. The horizontal solid line shows the baseline simulation and ther horizontal dotted lines illustrate the boundaries for bioequivalence.

To summarize the findings, The AUC_{inf} was most influenced by PBPK NAT2 V_{max}, whilst the C_{max} was mostly influenced by permeability, stomach transit time, and PBPK NAT2 V_{max}. None of the parameters tested within the range shown in the supplementary materials influenced the C_{max} beyond the bioequivalence limits, whilst the PBPK NAT2 V_{max} led to non-bioequivalent exposure in terms of AUC_{inf} within the range tested. Overall, the results are expected based on the BCS 1 classification for INH and a significant first-pass extraction in the gut and liver.

RIFAMPICIN

The parameters selected for the PSA are shown in **Table S4**. The physiology chosen for this simulation was an adult physiology. The dose selected RIF was 600 mg.

Table S4 Parameters Chosen for Sensitivity Analysis for RIF

Parameter	Baseline Value	Lower Bound	Upper Bound
Particle radius (µm)	25	2.5	250
Precipitation time (s)	500	50	5000
Solubility (mg/mL)	0.64	0.064	6.4
P_{eff} (10^{-4} cm/s)	2.11	1.055	4.22
Volume of the lower intestine (%)	2	1	4
Volume of the upper intestine (%)	20	10	40
Stomach transit time (h)	0.25	0.125	0.5

Small intestinal transit time (h)	3.3	1.65	6.6
Cecum transit time (h)	4.5	2.25	9
Colon transit time (h)	13.5	6.75	27
Stomach pH	2	0.5	8
PBPK CYP3A4 V_{max} (mg/s/mg-enz)	0.000406	0.000203	0.000812
PBPK CES2 V_{max} (mg/s/mg-enz)	0.000261	0.000131	0.000522
Gut CYP3A4 and CES2 V_{max} scale factor	1	0.5	2

Out of the parameters presented in **Table S4**, cecum transit time and colon transit time did not influence the PK of RIF. Results of the PSA for RIF are shown in **Figure S47**.

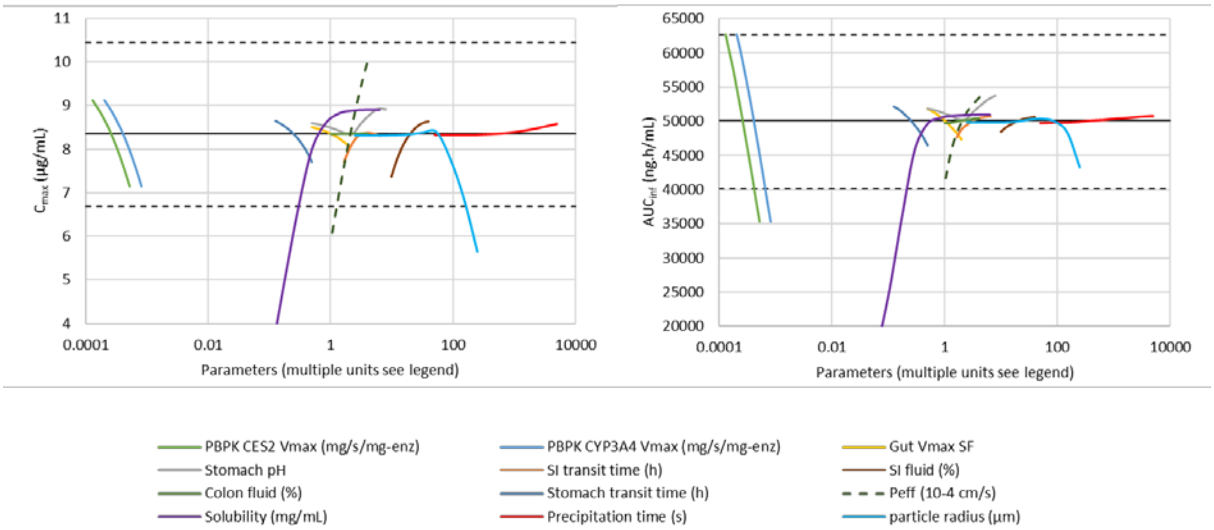

Figure S47 Effect parameters on RIF C_{max} (left panel) and AUC_{inf} (right panel). The horizontal solid line shows the baseline simulation and the horizontal dotted lines illustrate the boundaries for bioequivalence.

The results of the RIF PSA confirm that the main parameters influencing the drug PK are the solubility and drug particle size, which point to a solubility limitation of the drug and is expected owing to the BCS2 nature of the drug. In this respect, the fraction of volume in the small intestine increases the C_{max} since more drug can dissolve, and its permeation happens faster. The drug permeability increases the C_{max} and AUC by increasing the rate of absorption and reducing first-pass gut extraction. CYP3A4 and CES2 V_{max} in the liver affect the exposure to the drug and the Cmax as well. Other than this, the other parameters tested may influence the C_{max} and AUC of the drug to a lesser degree since none of the variables would lead to non-bioequivalent results compared to the reference exposure data.

MODEL APPLICATION

INDIVIDUAL PK PROFILES MODEL APPLICATION FOR INH

Individual INH profiles observed during study STPH71/10 for the test and reference products are shown in **Figure S48** and **Figure S49**. Individual RIF profiles observed during study STPH71/10 for the test and reference products are shown in **Figure S50** and **Figure S51**.

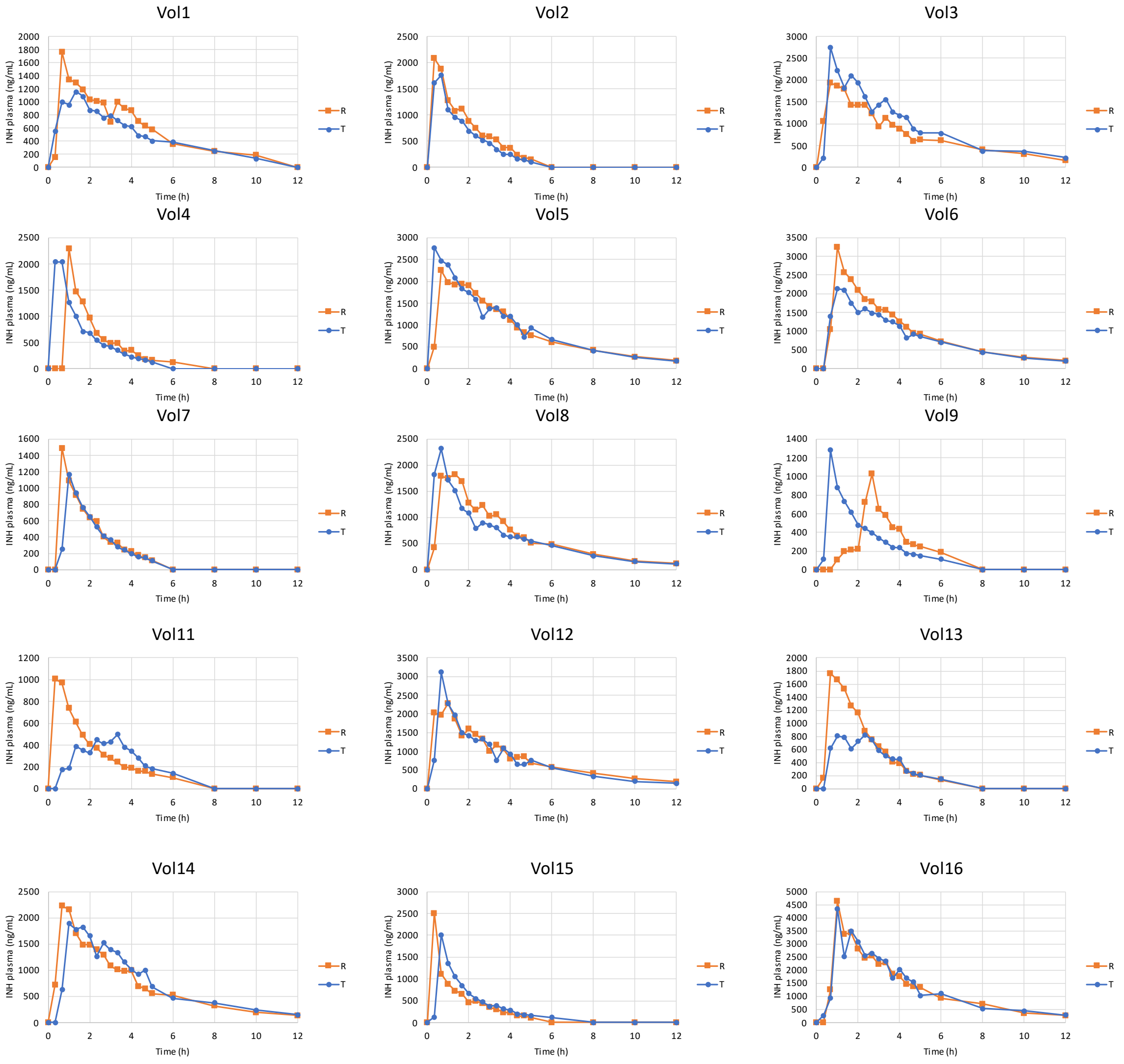

Figure S48 Individual PK Profiles for INH in STPH71/10 (Volunteer 1 to 16)

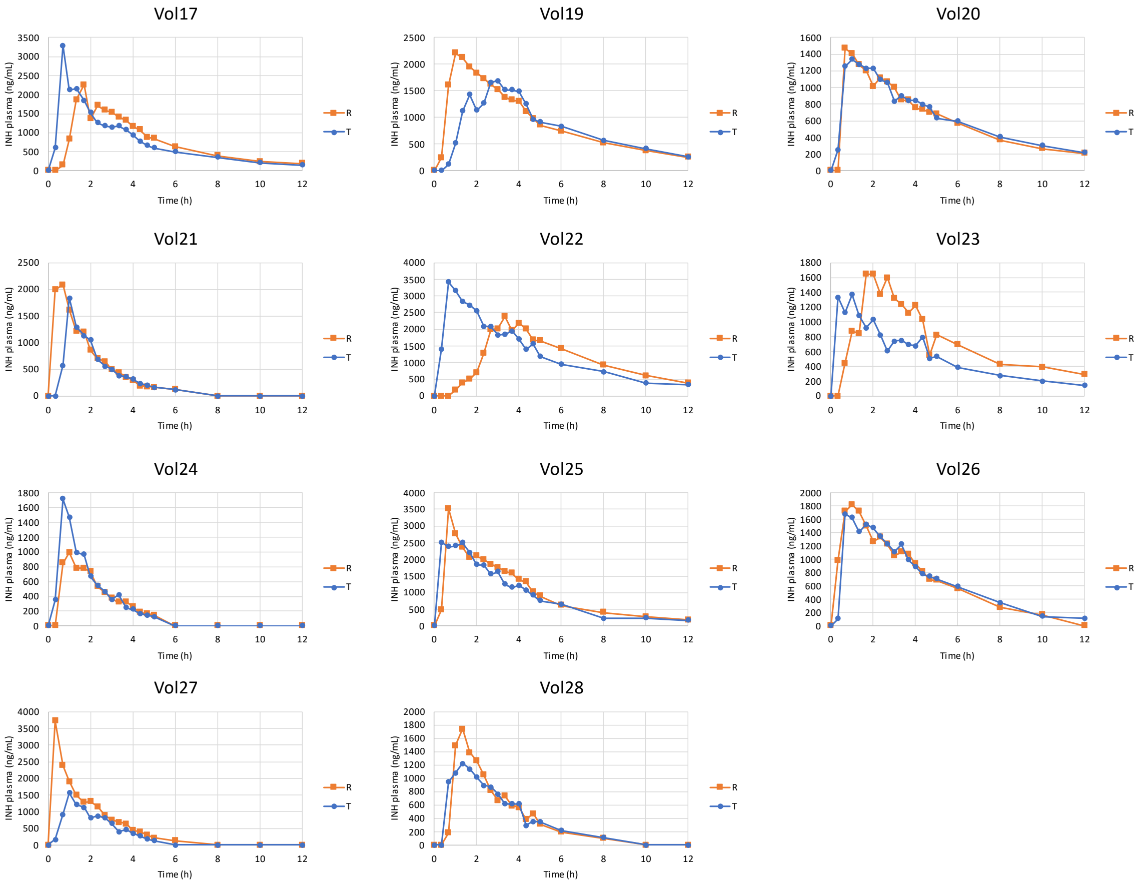

Figure S49 Individual PK Profiles for INH in STPH71/10 (Volunteer 17 to 28)

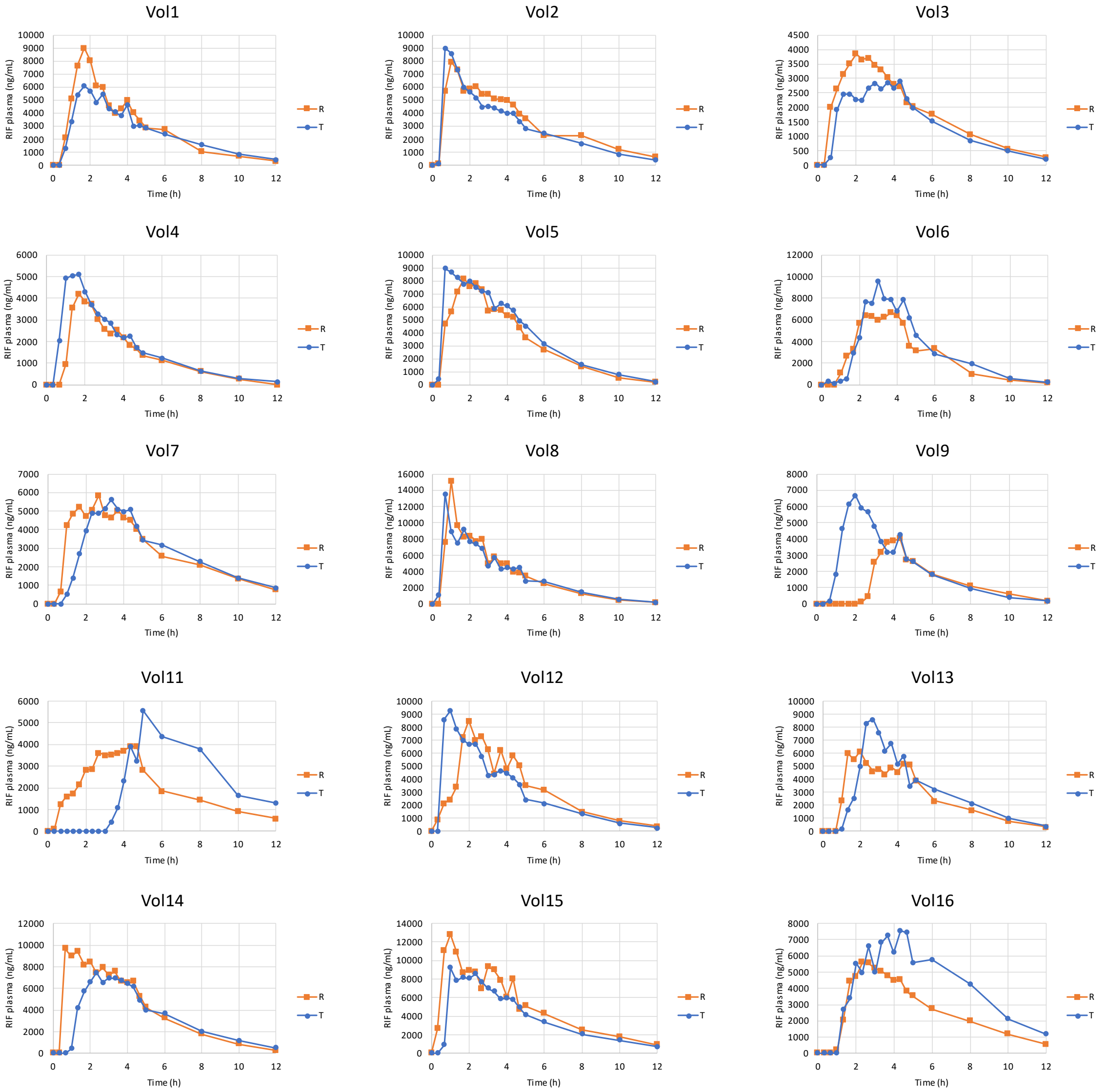

Figure S50 Individual PK Profiles for RIF in STPH71/10 (Volunteer 1 to 16)

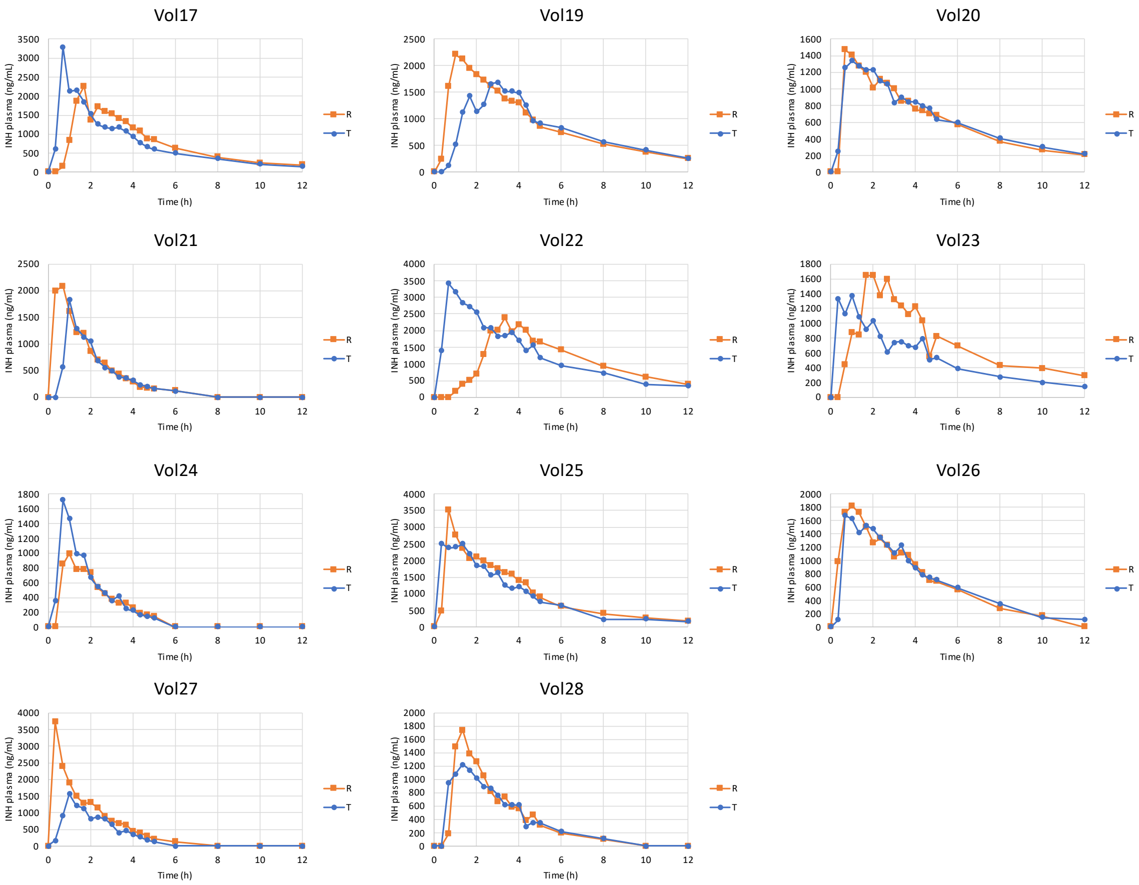

Figure S51 Individual PK Profiles for RIF in STPH71/10 (Volunteer 17 to 28)

An analysis of the occurrence of these multiple peaks did not reveal any relation to sex, body weight, acetylation status or formulation, and appears to be a random occurrence in the subjects. For INH and RIF since there is a first pass gut metabolism of these drugs, increase residence in the stomach or multi-phasic gastric release can dilute the drug in the lumen of the intestine and lead to more first pass metabolism. Consequently, test/reference C_{max} and AUC ratio for INH and RIF, appear well correlated to one another (**Figure S52**). Note that even if the BE study STPH71/10 was successful, the test/reference C_{max} ratio for INH was 90% and the 90% lower confidence interval almost missed the BE criteria with a value of 80.29%.

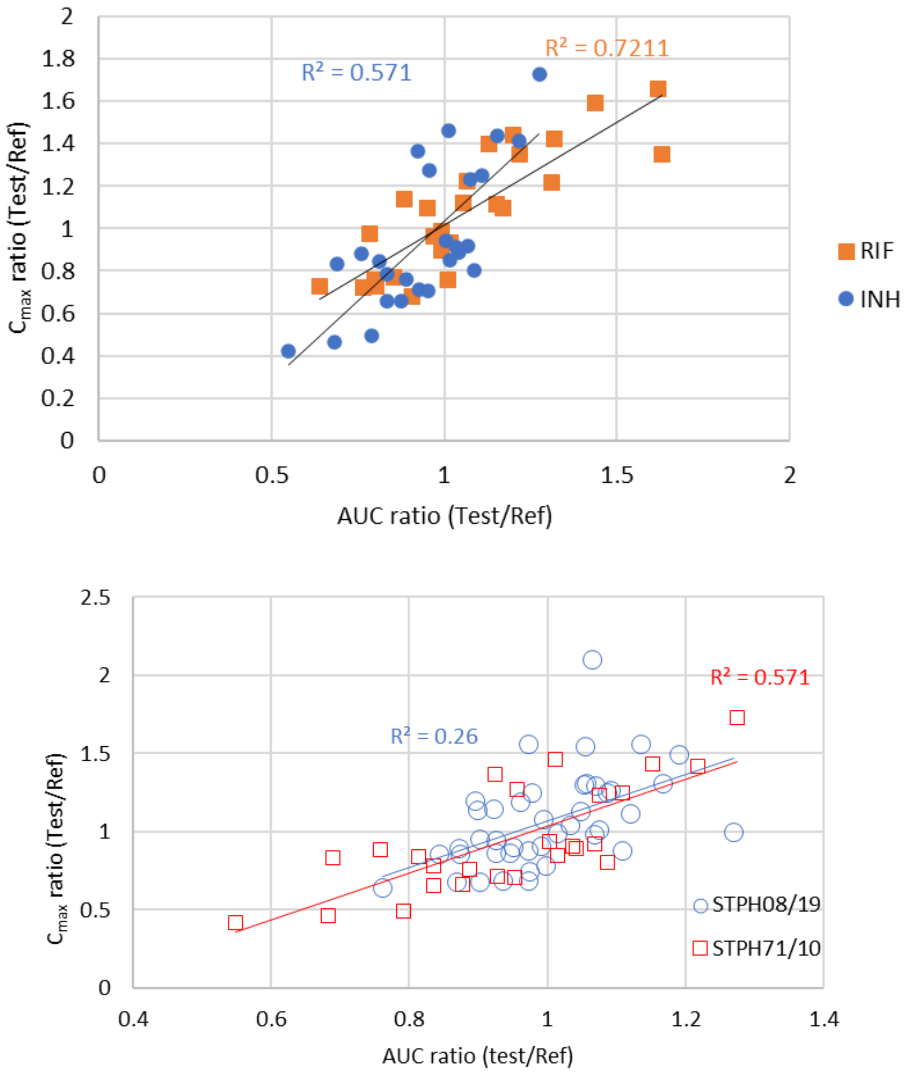

Figure S52 C_{max} ratio vs AUC ratio for INH and RIF in STPH71/10 (upper panel) and for INH in STPH71/10 and STPH08/19 (lower panel)

For comparison in study STPH08/19, where test and reference 100 mg INH tablets were administered, there were also occurrences of multi-phasic gastric emptying. There was however a lesser correlation between INH test/reference C_{max} and AUC ratio in this study (**Figure S52**). Even if the INH C_{max} ratios evolve in the same range in both studies due to the random occurrence of multi-phasic gastric emptying, the INH AUC ratios evolve in a much narrower range for STPH08/19 compared to STPH71/10. In other words, in the absence of RIF, INH shows a variable C_{max} due to gastric emptying, but the AUC ratio is less impacted. Since the pediatric formulation is a dispersible tablet to be administered with water, the difference in gastric emptying time compared to a solid oral combination product is anticipated to be large.

COMPARISON OF POPULATION SIMULATIONS FOR INH AND RIF TO MEASURED VALUES

The predicted vs measured AUC and C_{max} for INH across the dose range tested in the main article are shown respectively in **Figure S53** and **Figure S54**.

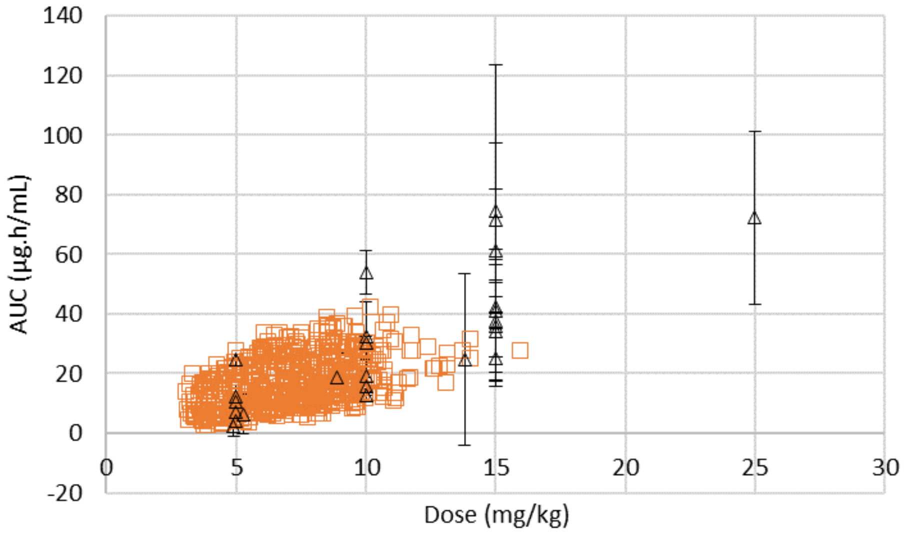

Figure S53 Predicted AUC as a function of dose in pediatric groups compared to values reported in the literature (black) [31-34, 36, 37, 48, 49].

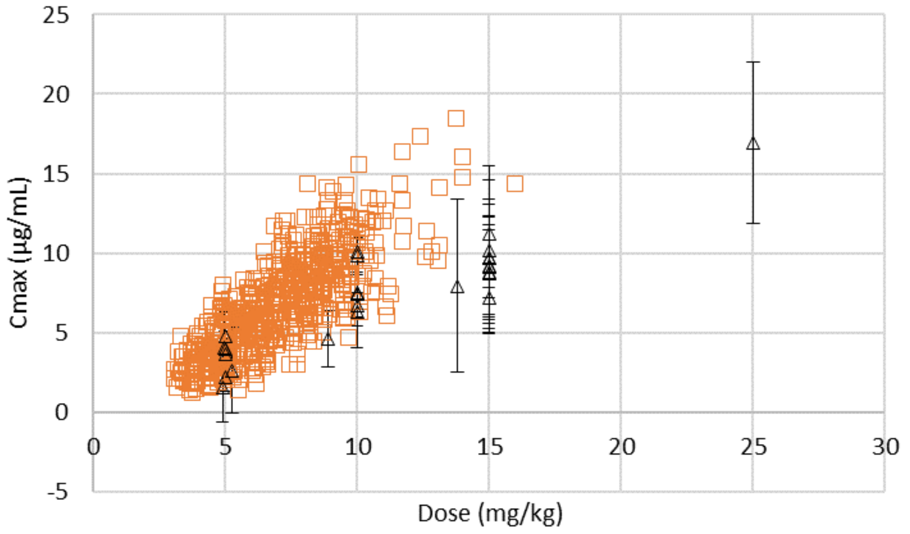

Figure S54 Predicted C_{max} as a function of dose in pediatric groups compared to values reported in the literature (black) [31-34, 36, 37, 48, 49].

The predicted RIF C_{max} compared to pediatric values reported in the literature for different doses of RIF as shown in **Figure S55**.

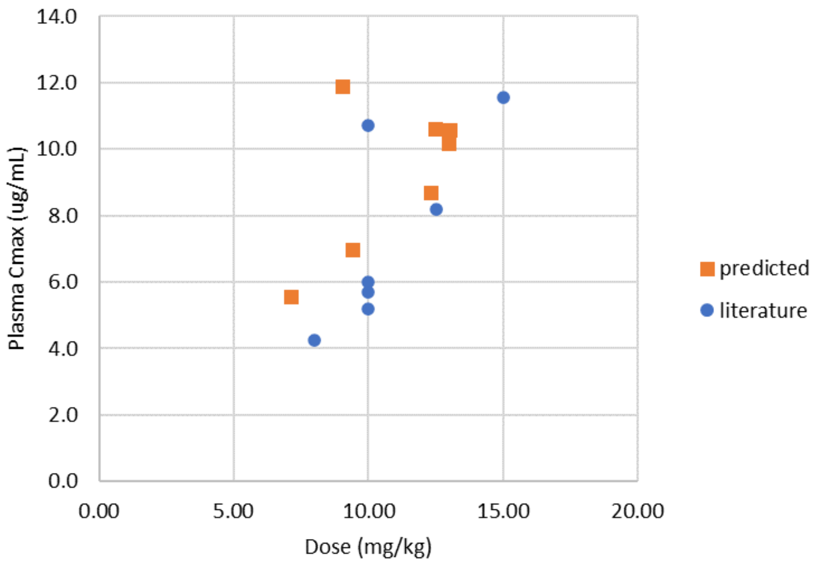

Figure S55 Predicted RIF C_{max} as a function of dose in pediatric compared to values reported in the literature [31, 39, 41, 42, 44].

The predicted AUC across the age groups for RIF using the GastroPlus model are compared to values obtained in the literature (**Figure S56**).

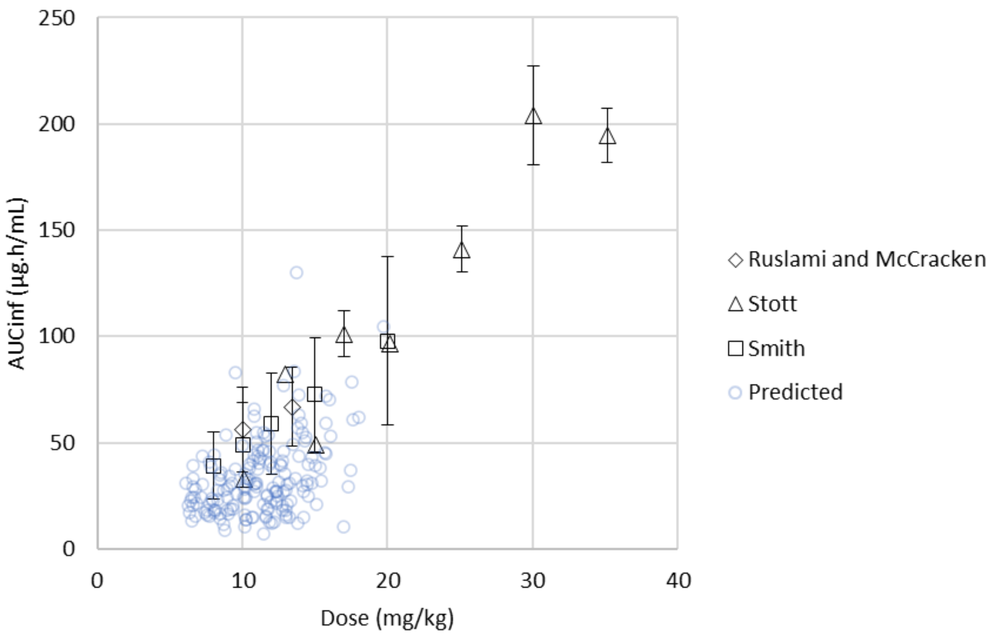

Figure S56 Predicted RIF AUC in pediatric subjects compared to average exposure in pediatric and adult subjects reported in the literature [41, 49-51].

1. PHYSIOLOGICAL PARAMETERS FOR RIF ACAT MODELS

Study #	Literature reference	Volume of fluid administered (mL)	Nature of fluid	pH in the stomach
3	Loos et al. [5]	200	Water	2*
5	Koup et al. [39]	5 (suspension)	Water	2.48**
6	Peloquin et al. [40]	250*	Water	2*(fasted), 4.9 (fed), 6 (Antacid)
7	McCracken et al. [41]	30 (suspension)	Water	2.44*
8	Schaaf et al. [42]	75	Water	2.75*
9	Acocella et al. [43]	200	Water	2*
10	Thee et al. [44]	75 (4y), 100 (8y), 150 (12y)	water	2.6* (4y), 2.27* (8y), 2.1* (12y)
11	Thee et al. [31]	3.5 (suspension)	Water	2.45**
14	Agrawal et al. [46] [47]	200	Water	2*
15	STPH71/10	200	Water	2*

*: Calculated based on volume dilution and pH of fluid using starting pH of 1.3
**: Calculated based on volume dilution and pH of fluid using starting pH of 2

Z-FACTOR FITTING FOR CLINICAL PRODUCTS AND TEST PRODUCTS

ISONIAZID

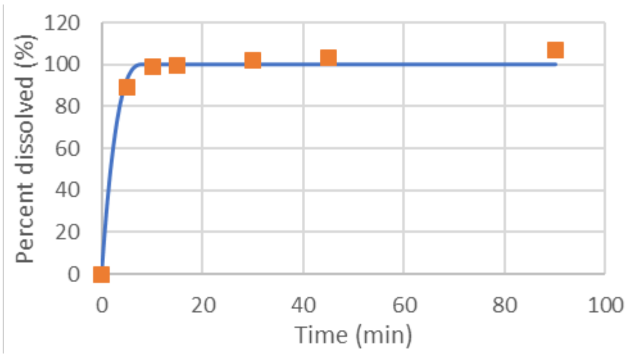

Figure S57 Dissolution profile for INH from 50+75 mg INH+RIF dispersible tablet batch 2110EX047 using 900 mL pH 6.8, USP2, 50 rpm (Solubility of 153 mg/mL).

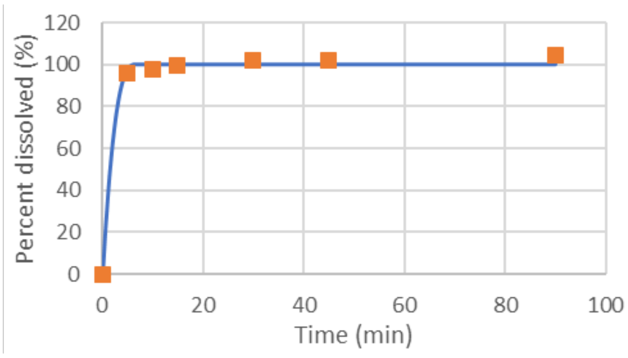

Figure S58 Dissolution profile for INH from 50+75 mg INH+RIF dispersible tablet batch 2111EX054 using 900 mL pH 6.8, USP2, 50 rpm (Solubility of 153 mg/mL).

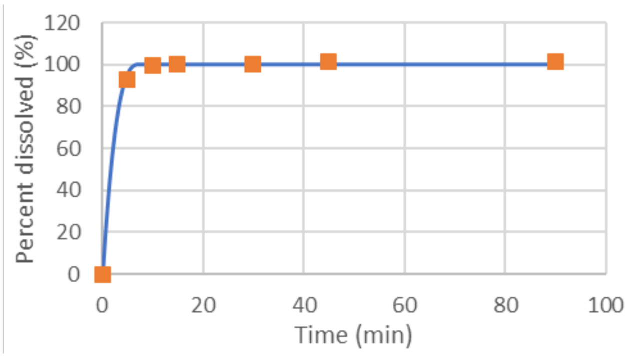

Figure S59 Dissolution profile for INH from 50+75 mg INH+RIF dispersible tablet batch 2206EX055 using 900 mL pH 6.8, USP2, 50 rpm (Solubility of 153 mg/mL).

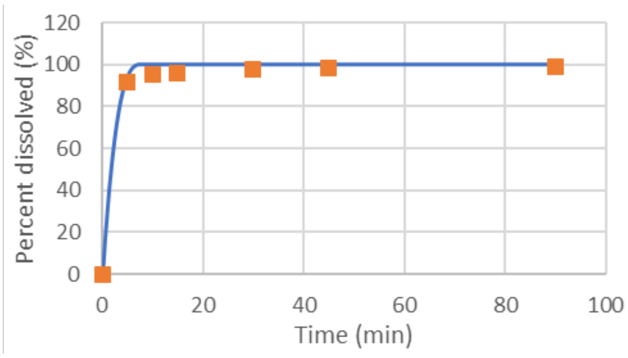

Figure S60 **Dissolution profile for INH from 50+75 mg INH+RIF dispersible tablet batch Macleods NRT2104A using 900 mL pH 6.8, USP2, 50 rpm (Solubility of 153 mg/mL).**

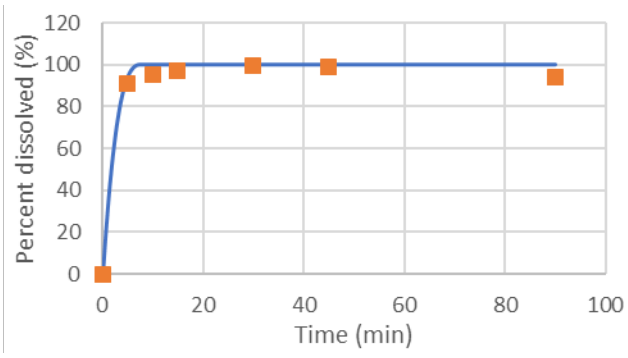

Figure S61 **Dissolution profile for INH from 50+75 mg INH+RIF dispersible tablet batch Macleods NRT9104 using 900 mL pH 6.8, USP2, 50 rpm (Solubility of 153 mg/mL).**

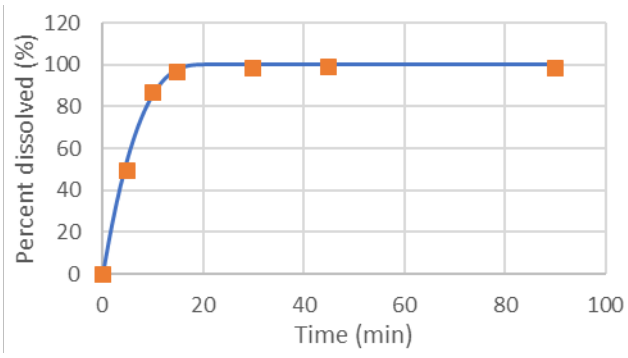

Figure S62 **Dissolution profile for INH from 150+300 mg INH+RIF coated tablet batch 22070874 using 900 mL pH 6.8, USP2, 50 rpm (Solubility of 153 mg/mL).**

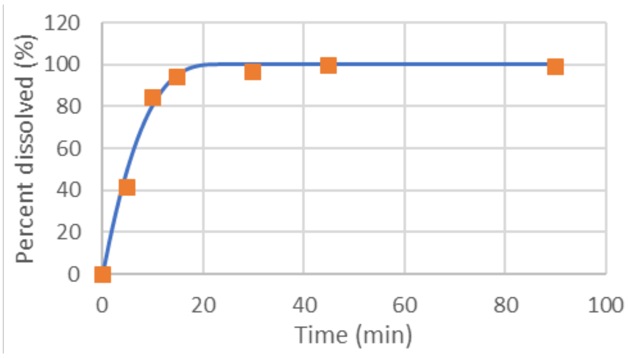

Figure S63 **Dissolution profile for INH from 150+300 mg INH+RIF coated tablet batch 22070875 using 900 mL pH 6.8, USP2, 50 rpm (Solubility of 153 mg/mL).**

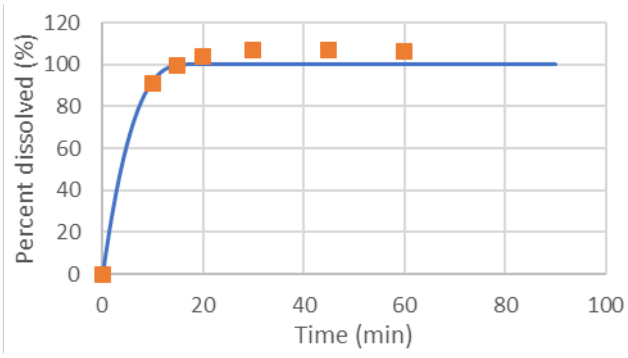

Figure S64 **Dissolution profile for INH from 150+300 mg INH+RIF coated tablet batch Rifinah® Batch A9362 using 900 mL pH 1.2, USP1, 100 rpm (Solubility of 174 mg/mL).**

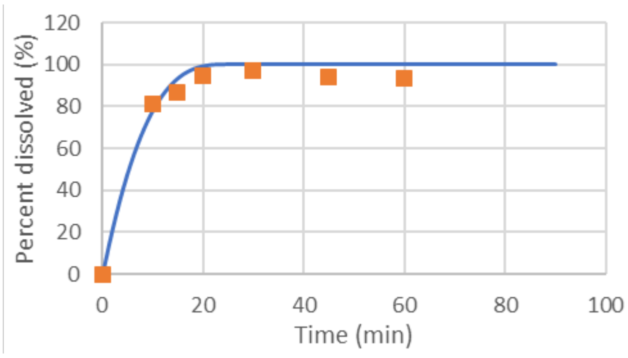

Figure S65 **Dissolution profile for INH from 150+300 mg INH+RIF coated tablet batch 09060664 using 900 mL pH 1.2, USP1, 100 rpm (Solubility of 174 mg/mL).**

Table S5 Z-factors for investigational batches to model INH dissolution

Batch number	Type of product	Dose of INH (mg)	Z-factor (mL/mg/s)	Equivalent radius (µm)*
FIOCRUZ/RJ 2110EX047	Dispersible	50	3.66E-05	2119
FIOCRUZ/RJ 2111EX054	Dispersible	50	4.50E-05	1723
FIOCRUZ/RJ 2206EX055	Dispersible	50	4.10E-05	1892
Macleods NRT2104A	Dispersible	50	3.92E-05	1978
Macleods NRT9104	Dispersible	50	3.90E-05	1989
FIOCRUZ/RJ 22070874	Coated	150	1.55E-05	5003
FIOCRUZ/RJ 22070875	Coated	150	1.37E-05	5661
Rifinah® Sanofi Batch A9362	Coated	150	2.26E-05	3432
FIOCRUZ/RJ batch 09060664	Coated	150	1.13E-05	6863

*: Calculated from Z-factor using $z = \frac{3D}{\rho h r_0}$ [52]

RIFAMPICIN

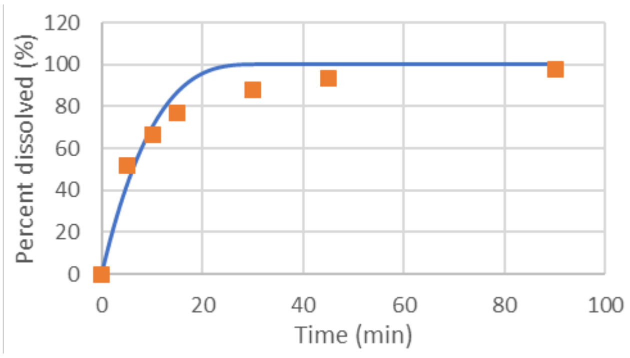

Figure S66 Dissolution profile for RIF from 50+75 mg INH+RIF dispersible tablet batch 2110EX047 using 900 mL pH 6.8, USP2, 50 rpm (Solubility of 1.2 mg/mL).

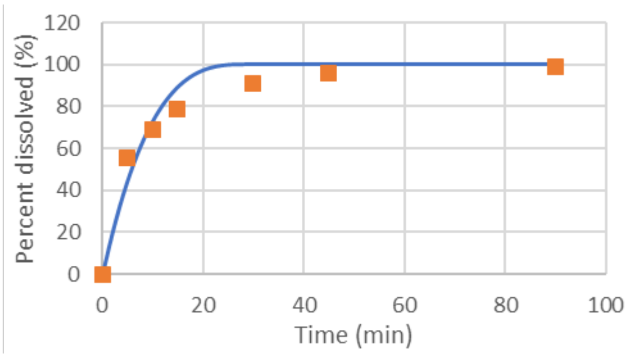

Figure S67 Dissolution profile for RIF from 50+75 mg INH+RIF dispersible tablet batch 2111EX054 using 900 mL pH 6.8, USP2, 50 rpm (Solubility of 1.2 mg/mL).

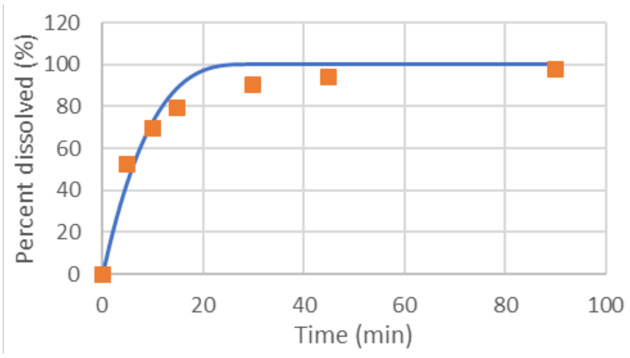

Figure S68 Dissolution profile for RIF from 50+75 mg INH+RIF dispersible tablet batch 2206EX055 using 900 mL pH 6.8, USP2, 50 rpm (Solubility of 1.2 mg/mL).

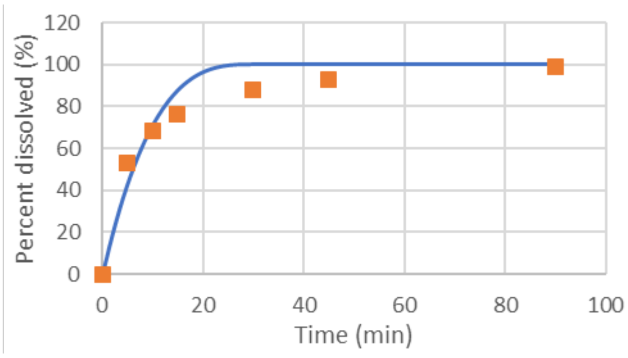

Figure S69 Dissolution profile for RIF from 50+75 mg INH+RIF dispersible tablet batch Macleods NRT2104A using 900 mL pH 6.8, USP2, 50 rpm (Solubility of 1.2 mg/mL).

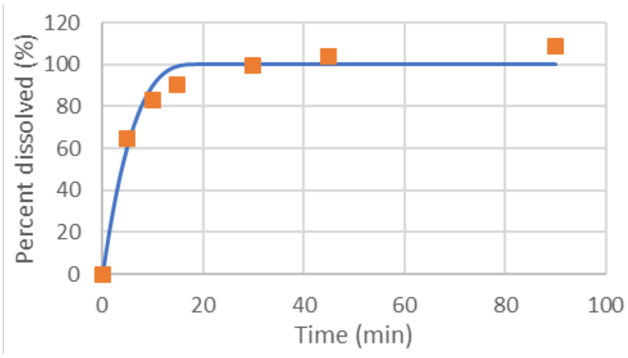

Figure S70 Dissolution profile for RIF from 50+75 mg INH+RIF dispersible tablet batch Macleods NRT9104 using 900 mL pH 6.8, USP2, 50 rpm (Solubility of 1.2 mg/mL).

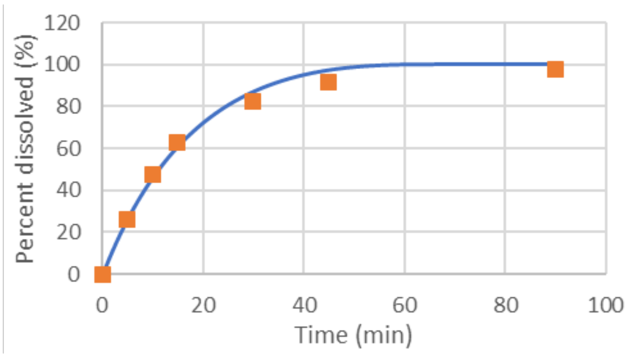

Figure S71 Dissolution profile for RIF from 150+300 mg INH+RIF coated tablet batch 22070874 using 900 mL pH 6.8, USP2, 50 rpm (Solubility of 1.2 mg/mL).

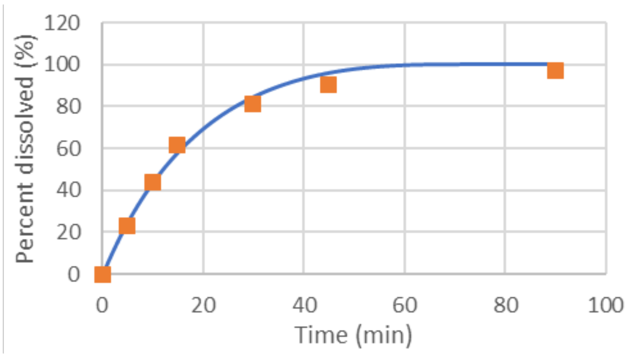

Figure S72 **Dissolution profile for RIF from 150+300 mg INH+RIF coated tablet batch 22070875 using 900 mL pH 6.8, USP2, 50 rpm (Solubility of 1.2 mg/mL).**

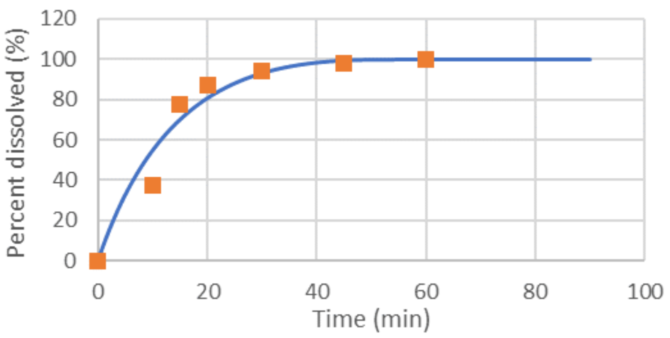

Figure S73 **Dissolution profile for RIF from 150+300 mg INH+RIF coated tablet batch Rifinah® Batch A9362 using 900 mL pH 7.4, USP1, 100 rpm (Solubility of 1.79 mg/mL) after data normalization to 100%.**

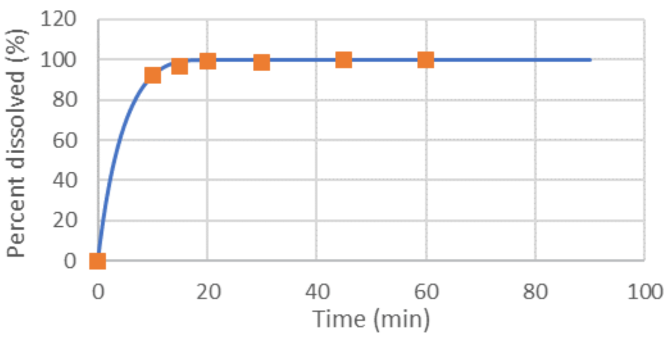

Figure S74 **Dissolution profile for RIF from 150+300 mg INH+RIF coated tablet batch 09060664 using 900 mL pH 7.4, USP1, 100 rpm (Solubility of 1.79 mg/mL) after data normalization to 100%.**

Table S6 Z-factors for investigational batches to model RIF dissolution

**Batch number**	**Type of product**	**Dose of RIF (mg)**	**Z-factor (mL/mg/s)**	**Equivalent radius (µm)***
FIOCRUZ/RJ 2110EX047	Dispersible	75	1.41E-03	30.3
FIOCRUZ/RJ 2111EX054	Dispersible	75	1.51E-03	29.1
FIOCRUZ/RJ 2206EX055	Dispersible	75	1.50E-03	29.2
Macleods NRT2104A	Dispersible	75	1.45E-03	29.7
Macleods NRT9104	Dispersible	75	2.30E-03	23.6
FIOCRUZ/RJ 22070874	Coated	300	8.24E-04	51.8
FIOCRUZ/RJ 22070875	Coated	300	7.65E-04	55.8
Rifinah® Sanofi Batch A9362	Coated	300	6.63E-04	64.4
FIOCRUZ/RJ batch 09060664	Coated	300	1.82E-03	26.5

*: Calculated from Z-factor using $z = \frac{3D}{\rho h r_0}$ [52]

CALCULATION OF MODEL PERFORMANCE INDICATORS FOR INH

ISONIAZID

Table S7 Calculation of model prediction performance for INH C_{max} in adults

						**AAPE(%)**	**AFE**
**Ref**	**Acetylator type**	**Prandial state**	**Dose (mg)**	**C_{max} measured (µg/mL)**	**C_{max} predicted (µg/mL)**	**5.1**	**1.03**
[53]	SA	Fasted	1000	20.1	20.2	0.8	0.004
[25]	RA	Fasted	300	5.2	4.5	13.6	-0.063
[25]	RA	Fasted	600	11.7	12.2	4.4	0.018
[25]	IA	Fasted	300	5.5	5.5	0.1	0.000
[25]	RA	Fasted	900	17.5	17.2	1.7	-0.007
[16]	RA	Fasted	300	3.6	4.5	25.4	0.098
[16]	SA	Fasted	300	7.2	6.7	6.2	-0.028
[16]	IA	Fasted	300	4.6	5.2	14.3	0.058
[26]	SA	Fasted	300	6.8	6.0	13.0	-0.060
[26]	SA	Milk	300	5.5	5.9	7.0	0.030
[26]	SA	High fat	300	4.9	5.9	20.9	0.083
[26]	SA	High sugar	300	4.3	4.8	10.8	0.044
[26]	RA	Fasted	300	7.2	6.6	9.0	-0.041
[26]	RA	Milk	300	6.0	5.2	14.1	-0.066
[26]	RA	High fat	300	4.3	4.7	9.2	0.038
[26]	RA	High sugar	300	3.6	3.6	0.6	-0.002
[30]	SA	Fasted	700	13.5	14.1	5.1	0.022
[30]	SA	Fasted	700	13.7	13.2	3.4	-0.015
[27]	RA	Fasted	300	4.1	5.3	29.8	0.113
[27]	SA	Fasted	300	4.9	6.3	29.3	0.112
[28]	SA	Fasted	300	4.8	4.7	0.8	-0.004
[29]	RA	Fasted	700	12.5	12.5	0.3	-0.001
[29]	RA	High sugar	700	3.8	4.2	8.7	0.036
STPH7 1/10-Ref	Mix with 0.5 SA	Fasted	150	2.0	1.8	12.1	-0.056
STPH7 1/10-Test	Mix with 0.5 SA	Fasted	150	1.8	1.7	7.8	-0.035

Table S8 Calculation of model prediction performance for INH AUC in adults

Ref	Acetylator type	Prandial state	Dose (mg)	AUC measured (μg.h/mL)	AUC predicted (μg.h/mL)	AAPE(%) 3.8	AFE 0.97
[53]	SA	Fasted	1000	89.6	84.9	5.3	-0.023
[25]	RA	Fasted	300	9.3	8.1	12.9	-0.060
[25]	RA	Fasted	600	24.2	23.3	3.7	-0.016
[25]	IA	Fasted	300	11.6	11.8	2.1	0.009
[25]	RA	Fasted	900	46.8	40.5	13.6	-0.064
[16]	RA	Fasted	300	9.4	9.0	4.2	-0.018
[16]	SA	Fasted	300	40.6	39.2	3.4	-0.015
[16]	IA	Fasted	300	15.6	13.5	13.4	-0.063
[26]	SA	Fasted	300	34.4	30.6	11.0	-0.051
[26]	SA	Milk	300	24.1	27.4	13.6	0.055
[26]	SA	High fat	300	22.9	29.8	30.4	0.115
[26]	SA	High sugar	300	19.5	22.6	16.0	0.064
[26]	RA	Fasted	300	15.8	15.8	0.0	0.000
[26]	RA	Milk	300	14.4	13.3	8.1	-0.037
[26]	RA	High fat	300	13.7	14.0	2.3	0.010
[26]	RA	High sugar	300	9.7	9.7	0.4	0.002
[30]	SA	Fasted	700	80.0	72.2	9.8	-0.045
[30]	SA	Fasted	700	87.7	71.2	18.8	-0.090
[27]	RA	Fasted	300	15.9	15.5	2.3	-0.010
[27]	SA	Fasted	300	29.9	31.3	4.5	0.019
[28]	SA	Fasted	300	18.2	18.8	2.8	0.012
[29]	RA	Fasted	700	22.0	24.0	9.1	0.038
[29]	RA	High sugar	700	12.8	9.5	25.9	-0.130
STPH7 1/10-Ref	Mix with 0.5 SA	Fasted	150	6.43	6.11	5.1	-0.023
STPH7 1/10-Test	Mix with 0.5 SA	Fasted	150	5.97	5.97	0.1	0.000

Table S9 Calculation of model prediction performance for INH C_{max} in pediatric subjects

Ref	Median age (years)	Acetylator type	Dose (mg/kg)	C_{max} measured (μg/mL)	C_{max} predicted (μg/mL)	AAPE(%) 7.03	AFE 1.08
[33]	9	NC	5	4.8	4.7	0.2	-0.001
[33]	9	NC	10	10.1	9.8	3.3	-0.014
[32]	4	Combined	25	16.9	25.2	48.8	0.173
[31]	1	RA	5	2.2	3.1	43.1	0.156
[31]	1	SA	5	4.0	4.7	18.5	0.074
[31]	1	IA	5	3.6	3.8	4.0	0.017
[31]	1	RA	10	6.7	7.6	13.1	0.053
[31]	1	SA	10	9.8	10.2	3.4	0.014
[31]	1	IA	10	7.5	8.7	16.5	0.066
[34]	1	SA	10	6.3	6.0	4.4	-0.020
[34]	3	RA	10	7.4	7.5	0.8	0.003
[36]	3	SA	4.98	4.1	3.7	8.9	-0.040
[36]	3	IA	5.26	2.6	2.4	9.0	-0.041
[36]	3	RA	4.9	1.5	1.4	11.7	-0.054
[37]	0.55	NC	13.8	7.9	10.6	33.5	0.125

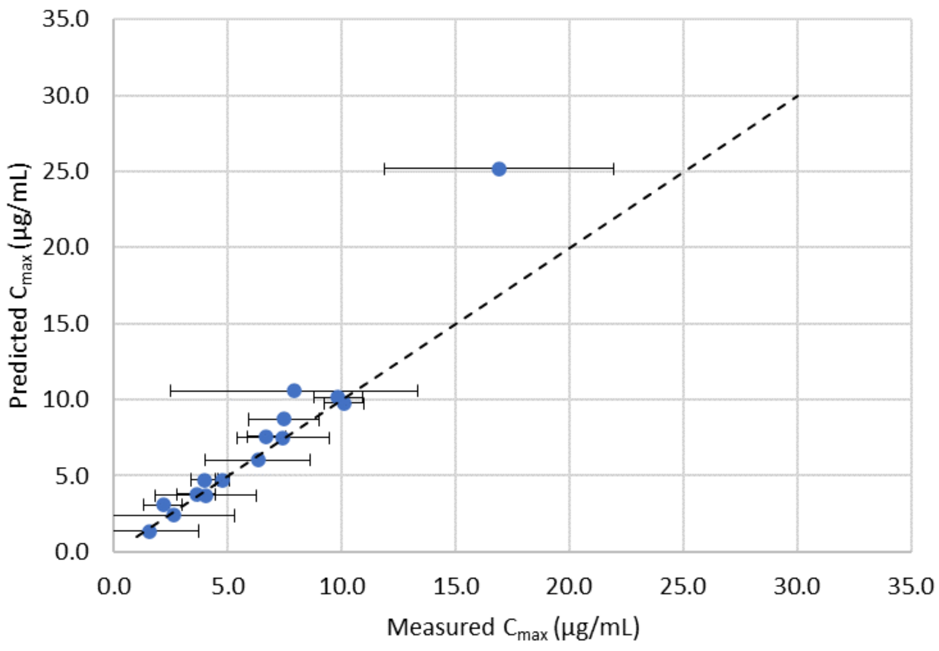

Figure S75 Predicted versus measured C_{max} in pediatric subjects. Error bars show one standard deviation

Table S10 Calculation of model prediction performance for INH AUC in pediatric subjects

Ref	Median age (years)	Acetylator type	Dose (mg/kg)	AUC measured (µg.h/mL)	AUC predicted (µg.h/mL)	AAPE(%)	AFE
						9.4	1.09
[33]	9	NC	5	24.5	18.8	23.4	-0.116
[33]	9	NC	10	54.1	40.8	24.6	-0.123
[32]	4	Combined	25	72.2	79.3	9.8	0.041
[31]	1	RA	5	4.3	5.0	16.0	0.065
[31]	1	SA	5	12.4	12.3	0.4	-0.002
[31]	1	IA	5	7.1	7.5	5.2	0.022
[31]	1	RA	10	12.9	13.8	7.2	0.030
[31]	1	SA	10	30.1	28.2	6.3	-0.028
[31]	1	IA	10	15.8	19.4	22.7	0.089
[34]	1	SA	10	32.4	31.7	2.3	-0.010
[34]	3	RA	10	19.3	19.2	0.8	-0.003
[36]	3	SA	4.98	10.6	12.5	17.5	0.070
[36]	3	IA	5.26	6.5	7.7	18.9	0.075
[36]	3	RA	4.9	2.3	4.5	95.9	0.292
[37]	0.55	NC	13.8	24.7	34.4	39.6	0.145

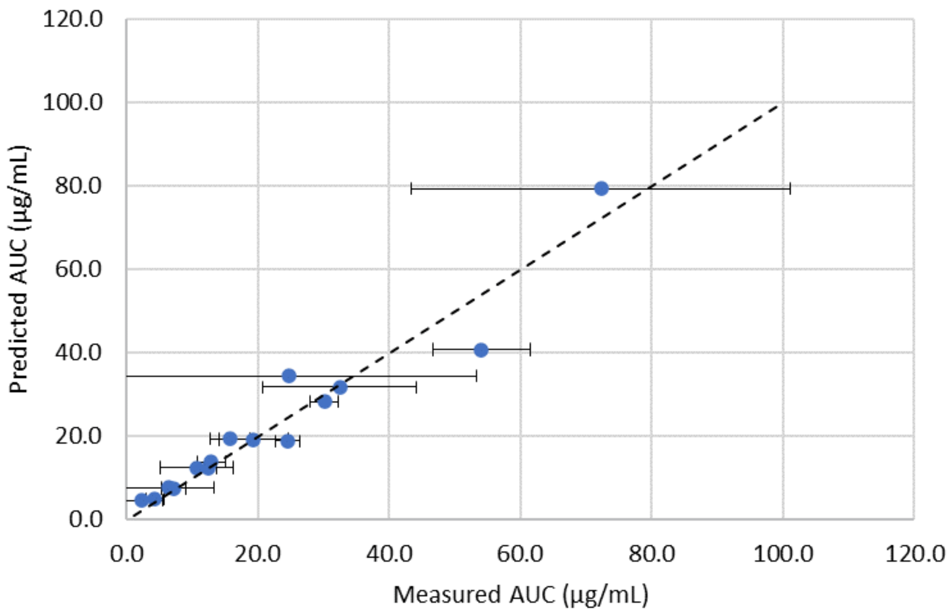

Figure S76 Predicted versus measured AUC in pediatric subjects. Error bars show one standard deviation

1.1 RIFAMPICIN

Table S11 Calculation of model prediction performance for RIF AUC in adult subjects

Ref	Reported AUC_{0-t} (µg.h/mL)	Predicted AUC_{0-t} (µg.h/mL)	AFE	AAPE(%)
			1.10	11.6
Stott	32.7	71.1	0.34	117.3
Stott	82.0	89.7	0.04	9.4
Stott	48.8	102.5	0.32	109.9
Stott	101.1	113.7	0.05	12.5
Stott	96.7	131.0	0.13	35.5
Stott	141.1	154.9	0.04	9.8
Stott	204.1	174.9	-0.07	14.3
Stott	194.8	191.7	-0.01	1.6
Wasserman 20mpk IV	222.5	189.8	-0.07	14.7
Loos 600 mg IV	109.7	86.4	-0.10	21.3
Loos 600 mg oral	66.0	70.0	0.03	6.1
Acocella 150 mg	8.7	13.3	0.19	53.9
Acocella 300 mg	22.6	29.6	0.12	30.8
Acocella 450 mg	42.1	45.4	0.03	7.9
Acocella 600 mg	58.5	61.5	0.02	5.1
Agrawal 450 mg C	18.3	27.7	0.18	50.9
Agrawal 450 mg D	41.4	36.4	-0.06	12.2
Agrawal 450 mg E	28.0	31.3	0.05	11.9
Agrawal 450 mg F	33.3	30.6	-0.04	8.1
Agrawal 600 mg G	52.4	48.0	-0.04	8.4
Agrawal 600 mg H	50.0	48.2	-0.02	3.5
Peloquin 600 mg fasted	56.8	55.7	-0.01	1.9
Peloquin 600 mg fed	53.2	57.9	0.04	8.9
Peloquin 600 mg ARA	53.5	55.1	0.01	3.0
STPH71-10 300 mg ref	32.9	31.0	-0.03	5.8
STPH71-10 300 mg test	33.7	31.5	-0.03	6.5

Table S12 Calculation of model prediction performance for RIF C_{max} in adult subjects

Ref	Reported C_{max} (µg/mL)	Predicted C_{max} (µg/mL)	AFE	AAPE(%)
			**1.15**	**10.8**
Wasserman 20mpk IV	40.9	47.9	0.068	17.0
Loos 600 mg IV	37.1	30.8	-0.081	17.1
Loos 600 mg oral	16.0	14.8	-0.033	7.4
Acocella 150 mg	1.8	2.6	0.162	45.1
Acocella 300 mg	3.7	5.3	0.150	41.3
Acocella 450 mg	6.1	7.8	0.109	28.4
Acocella 600 mg	9.2	10.3	0.047	11.5
Agrawal 450 mg C	3.1	5.3	0.229	69.4
Agrawal 450 mg D	7.5	7.4	-0.002	0.5
Agrawal 450 mg E	5.0	6.4	0.108	28.2
Agrawal 450 mg F	6.0	6.3	0.027	6.4
Agrawal 600 mg G	8.5	9.6	0.053	13.0
Agrawal 600 mg H	8.7	10.2	0.069	17.2
Peloquin 600 mg fasted	8.5	8.8	0.016	3.8
Peloquin 600 mg fed	6.0	9.1	0.183	52.5
Peloquin 600 mg ARA	8.6	8.7	0.006	1.4
STPH71-10 300 mg ref	5.2	5.0	-0.017	3.8
STPH71-10 300 mg test	5.3	5.4	0.008	1.9

Table S13 Calculation of model prediction performance for RIF plasma concentrations in pediatric subjects

**Ref**	**Route**	**Age (years)**	**BW (kg)**	**AFE**	**AAPE(%)**
				**1.14**	**28.5**
Nahata	IV	0.003-18	2.8-48	1.37	30
Koup	IV	7	26.54	0.65	33.5
Koup	IV	2	12.87	0.72	25
Koup	Oral	2	12.87	1.23	18.8
McCraken	Oral	2	12.4	0.79	20
Schaaf 8 mpk	Oral	4	13.91	1.65	60.4
Thee 2011 10mpk	Oral	1	10.23	1.85	44.6
Thee 2011 15mpk	Oral	1	10.23	1.18	45.6
Thee 2009 4y 10mpk	Oral	4	17.34	1.16	10.8
Thee 2009 8y 10mpk	Oral	8	30.31	1.26	14.3
Thee 2009 12y 10mpk	Oral	12	48.58	1.43	40.5

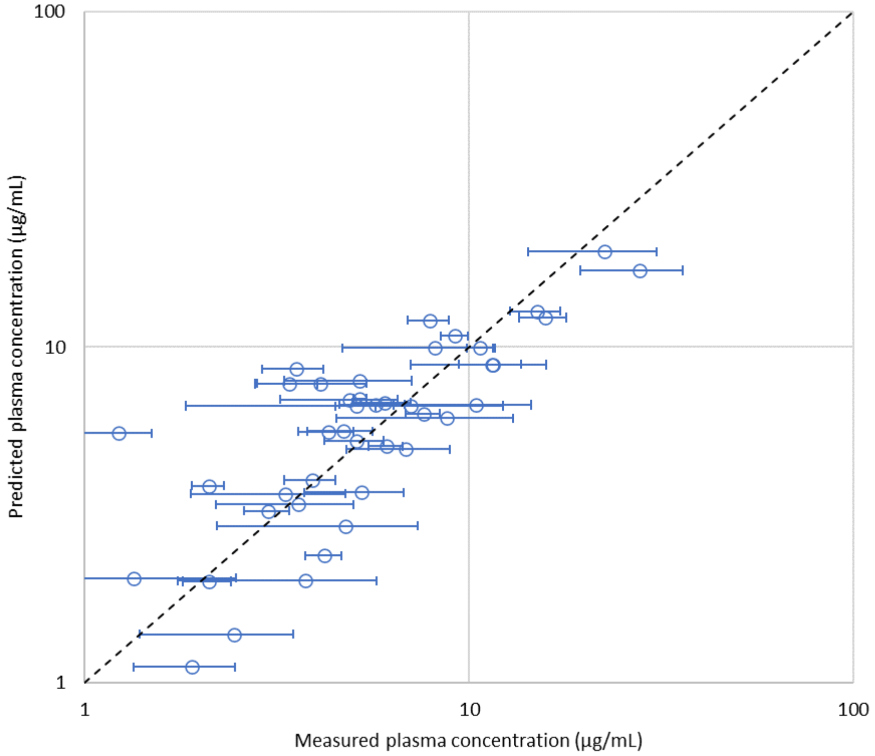

Figure S77 Predicted versus measured plasma concentrations in pediatric subjects for RIF. Error bars show one standard deviation.

2. NAT2 V_{MAX} FOR ALL REFERENCES

References	Acetylator type	Liver V_{max} mg/s/mg-enz	Gut V_{max} mg/s
Peloquin [54]	SA	0.001218	0.010602
Mannisto [26] / Pea [27] / Roy [33]	SA	0.0005	0.004352
Mannisto [26] / Pea [27] / Roy [33]	IA	0.00125	0.010881
Mannisto [26] / Pea [27] / Roy [33]	RA	0.002	0.017409
Gelber [30]	SA	0.000713	0.006206
Bing [16]	SA	0.0004176	0.003635
Boxenbaum [24] / Phaisal [32] / Thee [31] / Hutchings [55] / Schaaf [35] / Mcilleron [36] / STPH71/10	SA	0.001321	0.011499
Boxenbaum [24] /Bing [16] / Kubota [25] / Phaisal [32] / Thee [31] / Schaaf [35] / Mcilleron [36] / STPH71/10	IA	0.0031305	0.027249
Boxenbaum [24] / Bing [16] / Kubota [25] / Phaisal [32] / Thee [31] / Schaaf [35] / Mcilleron [36] / Melander [29] / Hutchings [55] / STPH71/10	RA	0.00494	0.043
Rey [34]	RA	0.00347	0.030204
Rey [34]	SA	0.0009105	0.007925

The gut V_{max} to liver V_{max} ratio is constant.

3. FITTING DRUG PRODUCT DISSOLUTION DATA OF RIF FIXED DOSE COMBINATIONS IN AGRAWAL ET AL. [46]

6 Fixed dose combination coated tablets were tested by Agrawal et al in vitro and entered in BE studies. All the formulations were tested according to pharmaceutical specifications and passed quality control tests with respect to uniformity of tablet weight, content of active drug substance and dissolution (USP, 2003). Composition of the FDC and dose of RIF tested in the study are shown in **Table S14**.

Table S14 Details of the bioequivalence trials of FDC formulations vs. separate formulations of anti-TB drugs conducted at NIPER bioavailability center. From [46]

Study code[a]	Dose[b] (mg)	FDC[c]	FDC strength[d] (mg)				Separate R formulations[e]	Formulations[f]		Number of Volunteers[g]	Sampling time[h] (h)
			R	H	Z	E		FDC	Separate		
Study C	450	Tablet/4 drug	150	75	400	275	Capsule: 450 mg	3	4	13	36
Study D	450	Tablet/4 drug	150	75	400	275	Capsule: 450 mg	3	4	14	24
Study E	450	Tablet/4 drug	150	75	400	275	Tablet: 450 mg	3	4	13	24
Study F	450	Tablet/4 drug	225	150	750	400	Capsule: 450 mg	2	5	14	24
Study G	600	Tablet/4 drug	150	75	400	275	Capsule: 300 mg	4	7	22	24
Study H	600	Tablet/3 drug	150	75	400	–	Capsule: 300 mg	4	5	19	24

Dissolution studies were done in 900 mL, 37°C, USP 2 apparatus at different agitation speeds (30, 50, 75, 100 rpm) and also at various pH (0.01N HCl, 0.1N HCl and 6.8 pH buffer) as rifampicin shows pH dependent solubility corresponding to physiologic pH of the GI tract segments. All formulations showed some sedimentation at 30 rpm. Formulations C, D, F and H displayed dissolution rates which were not sensitive to agitation from 50 rpm onwards, whilst formulations E and G showed impact of agitation until 75 rpm. In order to accommodate the potential effect of sedimentation on the drug dissolution, the P-PSD HDC was applied to the dissolution data of these formulations [56].

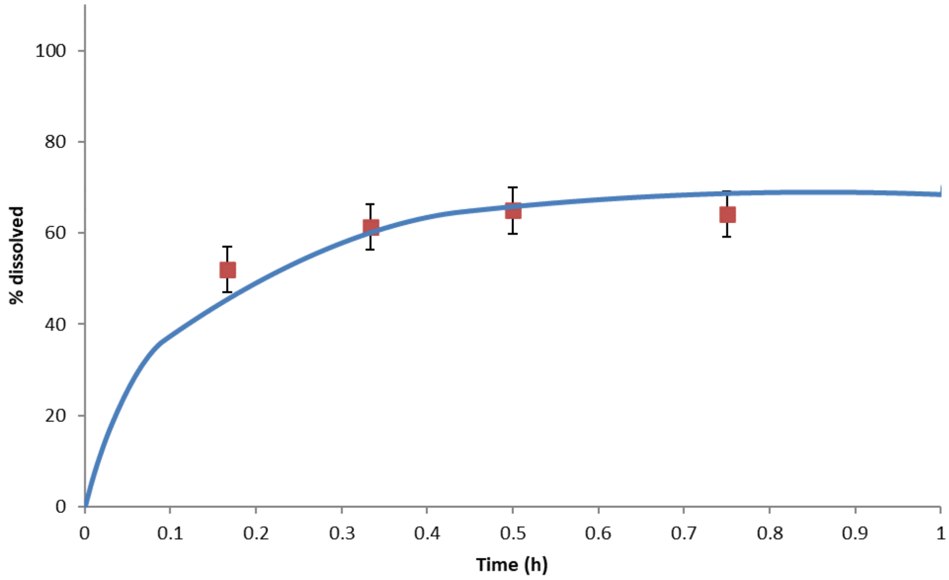

Figure S78 P-PSD Fitting for 150 mg FDC Tablet C Using pH2 Dissolution Method at 50 rpm

Table S15 P-PSD for 150 mg FDC Tablet C

Radius Microns	Percent w/w Distribution
30	20
75	27
150	53

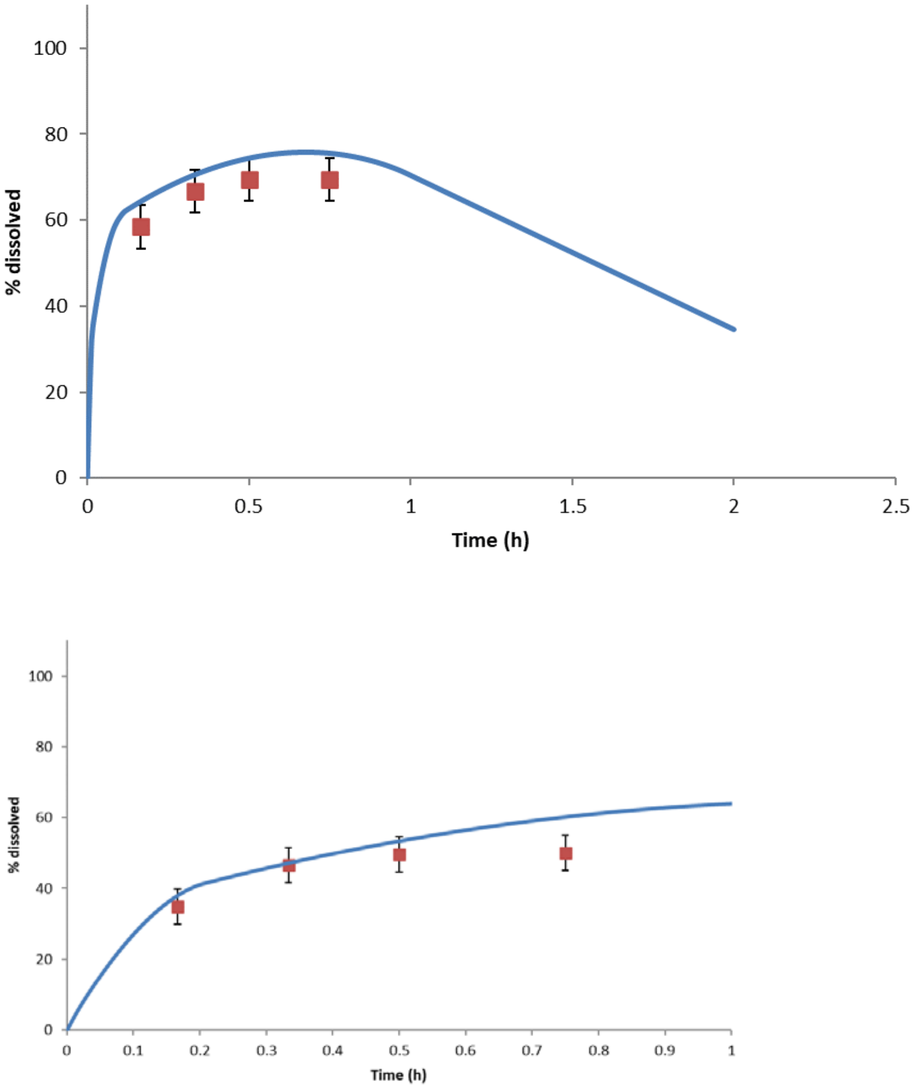

Figure S79 P-PSD Fitting for 150 mg FDC Tablet D Using pH2 Dissolution Method at 75 rpm (upper panel) and 30 rpm (lower panel)

Table S16 P-PSD for 150 mg FDC Tablet D

Radius Microns	Percent w/w Distribution
35	28
120	72

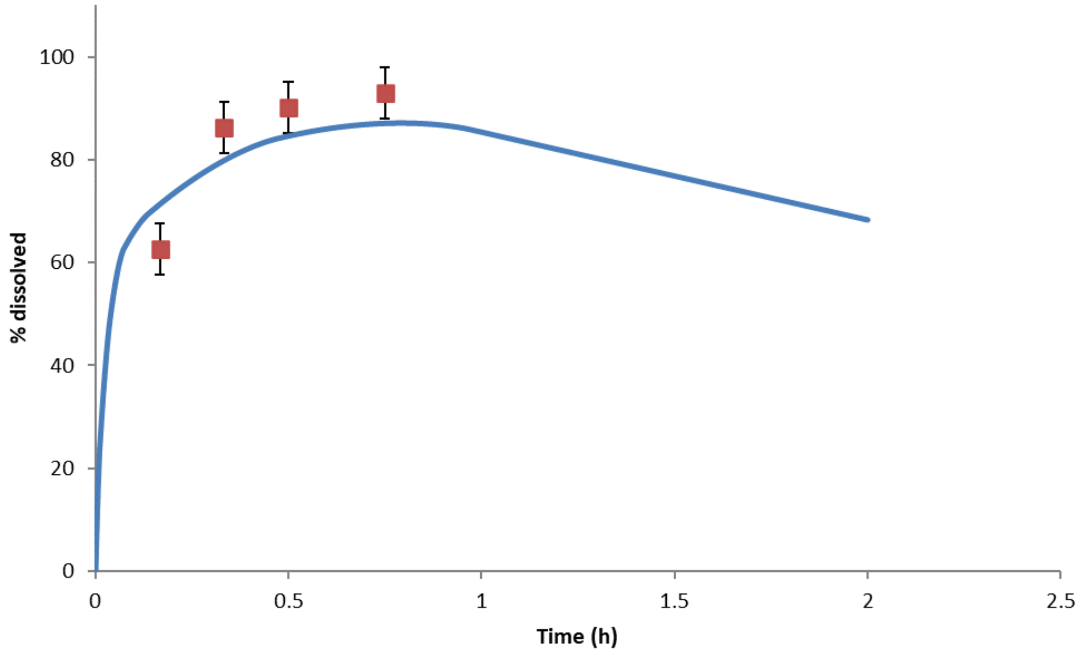

Figure S80 **P-PSD Fitting for 150 mg FDC Tablet E Using pH2 Dissolution Method at 50 rpm**

Table S17 **P-PSD for 150 mg FDC Tablet E**

Radius Microns	Percent w/w Distribution
25	18.6
50	20.6
75	60.8

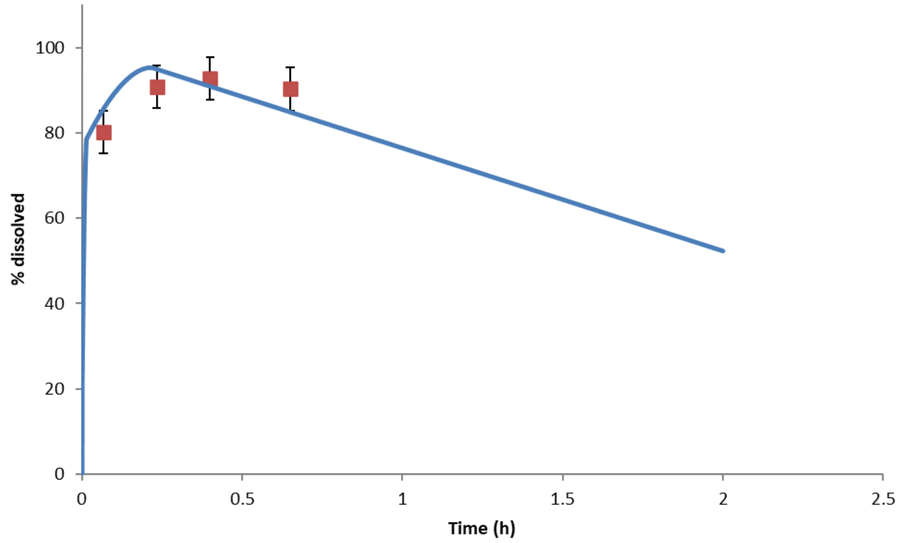

Figure S81 P-PSD Fitting for 225 mg FDC Tablet F Using pH2 Dissolution Method at 75 rpm

Table S18 P-PSD for 225 mg FDC Tablet F

Radius Microns	Percent w/w Distribution
5	25
30	75

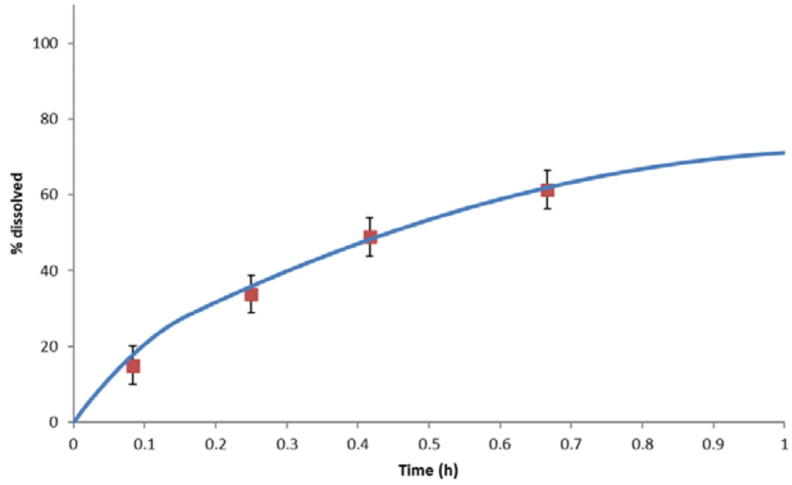

Figure S82 P-PSD Fitting for 150 mg FDC Tablet G Using pH2 Dissolution Method at 30 rpm

Table S19 P-PSD for 150 mg FDC Tablet G

Radius Microns	Percent w/w Distribution
30	10
105	90

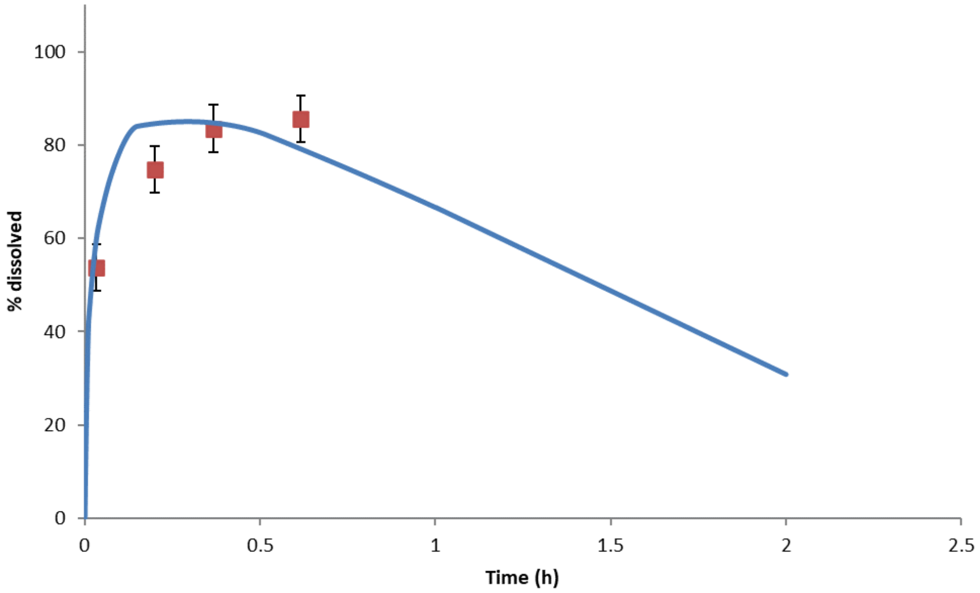

Figure S83 **P-PSD Fitting for 150 mg FDC Tablet H Using pH2 Dissolution Method at 50 rpm**

Table S20 **P-PSD for 150 mg FDC Tablet H**

Radius Microns	Percent w/w Distribution
25	60
50	30
75	10

REFERENCES

1. Becker, C., et al., *Biowaiver monographs for immediate release solid oral dosage forms: isoniazid.* J Pharm Sci, 2007. **96**(3): p. 522-31.
2. Becker, C., et al., *Biowaiver monographs for immediate release solid oral dosage forms: rifampicin.* J Pharm Sci, 2009. **98**(7): p. 2252-67.
3. Ermondi, G., et al., *Rifampicin as an example of beyond-rule-of-5 compound: Ionization beyond water and lipophilicity beyond octanol/water.* European Journal of Pharmaceutical Sciences, 2021. **161**: p. 105802.
4. Agrawal, S. and R. Panchagnula, *Implication of biopharmaceutics and pharmacokinetics of rifampicin in variable bioavailability from solid oral dosage forms.* Biopharm Drug Dispos, 2005. **26**(8): p. 321-34.
5. Loos, U., et al., *Pharmacokinetics of oral and intravenous rifampicin during chronic administration.* Klin Wochenschr, 1985. **63**(23): p. 1205-11.
6. Chen, Y.-J., *The solubility enhancement and the stability assessment of rifampicin, isoniazid and pyrazinamide in aqueous media.* 2001, Rhodes University.
7. Singh, S., et al., *Degradation of Rifampicin, Isoniazid and Pyrazinamide from Prepared Mixtures and Marketed Single and Combination Products Under Acid Conditions.* Pharmacy and Pharmacology Communications, 2010. **6**: p. 491-494.
8. Seydel, J.K., *Physico-chemical studies on rifampicin.* Antibiot Chemother, 1970. **16**: p. 380-91.
9. Prankerd, R.J., J.M. Walters, and J.H. Parnes, *Kinetics for degradation of rifampicin, an azomethine-containing drug which exhibits reversible hydrolysis in acidic solutions.* International Journal of Pharmaceutics, 1992. **78**(1-3): p. 59-67.
10. Mariappan, T.T. and S. Singh, *Regional gastrointestinal permeability of rifampicin and isoniazid (alone and their combination) in the rat.* Int J Tuberc Lung Dis, 2003. **7**(8): p. 797-803.
11. Devani, M.B., et al., *Kinetic studies of the interaction between isoniazid and reducing sugars.* J Pharm Sci, 1985. **74**(4): p. 427-32.
12. Pepin, X.J.H. and S. Suarez-Sharp, *Effect of Food Composition on the PK of Isoniazid Quantitatively Explained Using Physiologically Based Biopharmaceutics Modeling.* The AAPS Journal, 2024. **26**(3): p. 54.
13. Keller, G.A., et al., *Age-distribution and genotype-phenotype correlation for N-acetyltransferase in Argentine children under isoniazid treatment.* Int J Clin Pharmacol Ther, 2014. **52**(4): p. 292-302.
14. Zanrosso, C.W., et al., *N -Acetyltransferase 2 Polymorphisms and Susceptibility to Infant Leukemia with Maternal Exposure to Dipyrone*

during Pregnancy. Cancer Epidemiology, Biomarkers & Prevention, 2010. **19**(12): p. 3037-3043.
15. Chen, B., et al., *Population Pharmacokinetics and Pharmacodynamics of Isoniazid and its Metabolite Acetylisoniazid in Chinese Population*. Front Pharmacol, 2022. **13**: p. 932686.
16. Bing, C., C. Xiaomeia, and L. Jinhenga, *Gene dose effect of NAT2 variants on the pharmacokinetics of isoniazid and acetylisoniazid in healthy Chinese subjects*. Drug metabolism and drug interactions, 2011. **26**(3): p. 113-8.
17. Wasserman, S., et al., *Plasma pharmacokinetics of high-dose oral versus intravenous rifampicin in patients with tuberculous meningitis: a randomized controlled trial*. Antimicrobial agents and chemotherapy, 2021. **65**(8): p. e00140-21.
18. Yamashita, F., et al., *Modeling of Rifampicin-Induced CYP3A4 Activation Dynamics for the Prediction of Clinical Drug-Drug Interactions from In Vitro Data*. PLOS ONE, 2013. **8**(9): p. e70330.
19. Budha, N.R., R.E. Lee, and B. Meibohm, *Biopharmaceutics, pharmacokinetics and pharmacodynamics of antituberculosis drugs*. Curr Med Chem, 2008. **15**(8): p. 809-25.
20. Kasim, N.A., et al., *Molecular Properties of WHO Essential Drugs and Provisional Biopharmaceutical Classification*. Molecular Pharmaceutics, 2004. **1**(1): p. 85-96.
21. Ranaldi, G., K. Islam, and Y. Sambuy, *Epithelial cells in culture as a model for the intestinal transport of antimicrobial agents*. Antimicrobial agents and chemotherapy, 1992. **36**(7): p. 1374-1381.
22. Biganzoli, E., et al., *Use of a Caco-2 cell culture model for the characterization of intestinal absorption of antibiotics*. Farmaco, 1999. **54**(9): p. 594-9.
23. Andreas, C.J., et al., *Mechanistic investigation of the negative food effect of modified release zolpidem*. European journal of pharmaceutical sciences, 2017. **102**: p. 284-298.
24. Boxenbaum, H. and S. Riegelman, *Pharmacokinetics of isoniazid and some metabolites in man*. Journal of Pharmacokinetics and Biopharmaceutics, 1976. **4**(4): p. 287-325.
25. Kubota, R., et al., *Dose-escalation study of isoniazid in healthy volunteers with the rapid acetylator genotype of arylamine N-acetyltransferase 2*. European Journal of Clinical Pharmacology, 2007. **63**(10): p. 927-933.
26. Männisto, P., et al., *Influence of various diets on the bioavailability of isoniazid*. The Journal of antimicrobial chemotherapy, 1982. **10**(5): p. 427-34.
27. Pea, F., et al., *Isoniazid and its Hydrazine Metabolite in Patients with Tuberculosis*. Clinical Drug Investigation, 1999. **17**(2): p. 145-154.

28. Peloquin, C.A., et al., *Population pharmacokinetic modeling of isoniazid, rifampin, and pyrazinamide.* Antimicrob Agents Chemother, 1997. **41**(12): p. 2670-9.
29. Melander, A., et al., *Reduction of Isoniazid Bioavailability in Normal Men by Concomitant Intake of Food.* Acta Medica Scandinavica, 2009. **200**(1-6): p. 93-97.
30. Gelber, R., P. Jacobsen, and L. Levy, *A study of the availability of six commercial formulations of isoniazid.* Clinical pharmacology and therapeutics, 1969. **10**(6): p. 841-8.
31. Thee, S., et al., *Pharmacokinetics of isoniazid, rifampin, and pyrazinamide in children younger than two years of age with tuberculosis: evidence for implementation of revised World Health Organization recommendations.* Antimicrob Agents Chemother, 2011. **55**(12): p. 5560-7.
32. Phaisal, W., et al., *Pharmacokinetics of isoniazid and rifapentine in young pediatric patients with latent tuberculosis infection.* International Journal of Infectious Diseases, 2022. **122**: p. 725-732.
33. Roy, V., U. Tekur, and K. Chopra, *Pharmacokinetics of isoniazid in pulmonary tuberculosis--a comparative study at two dose levels.* Indian Pediatr, 1996. **33**(4): p. 287-91.
34. Rey, E., et al., *Isoniazid pharmacokinetics in children according to acetylator phenotype.* Fundamental & clinical pharmacology, 2001. **15**(5): p. 355-9.
35. Schaaf, H.S., et al., *Isoniazid pharmacokinetics in children treated for respiratory tuberculosis.* Arch Dis Child, 2005. **90**(6): p. 614-8.
36. McIlleron, H., et al., *Isoniazid plasma concentrations in a cohort of South African children with tuberculosis: implications for international pediatric dosing guidelines.* Clin Infect Dis, 2009. **48**(11): p. 1547-53.
37. Bekker, A., et al., *Pharmacokinetics of Rifampin, Isoniazid, Pyrazinamide, and Ethambutol in Infants Dosed According to Revised WHO-Recommended Treatment Guidelines.* Antimicrobial Agents and Chemotherapy, 2016. **60**(4): p. 2171-2179.
38. Koup, J.R., et al., *Pharmacokinetics of Rifampin in Children I. Multiple Dose Intravenous Infusion.* Therapeutic Drug Monitoring, 1986. **8**(1).
39. Koup, J.R., et al., *Pharmacokinetics of Rifampin in Children II. Oral Bioavailability.* Therapeutic Drug Monitoring, 1986. **8**(1).
40. Peloquin, C.A., et al., *Pharmacokinetics of Rifampin Under Fasting Conditions, With Food, and With Antacids.* Chest, 1999. **115**(1): p. 12-18.
41. McCracken, G.H., Jr, et al., *Pharmacokinetics of Rifampin in Infants and Children: Relevance to Prophylaxis Against Haemophilus influenzae Type b Disease.* Pediatrics, 1980. **66**(1): p. 17-21.
42. Schaaf, H.S., et al., *Rifampin pharmacokinetics in children, with and without human immunodeficiency virus infection, hospitalized for the*

management of severe forms of tuberculosis. BMC Medicine, 2009. **7**(1): p. 19.
43. Acocella, G., *Clinical Pharmacokinetics of Rifampicin.* Clinical Pharmacokinetics, 1978. **3**(2): p. 108-127.
44. Thee, S., et al., *Rifampicin serum levels in childhood tuberculosis.* Int J Tuberc Lung Dis, 2009. **13**(9): p. 1106-11.
45. Nahata, M.C., et al., *Pharmacokinetics, cerebrospinal fluid concentration, and safety of intravenous rifampin in pediatric patients undergoing shunt placements.* European Journal of Clinical Pharmacology, 1990. **38**(5): p. 515-517.
46. Agrawal, S. and R. Panchagnula, *Dissolution test as a surrogate for quality evaluation of rifampicin containing fixed dose combination formulations.* Int J Pharm, 2004. **287**(1-2): p. 97-112.
47. Agrawal, S., et al., *Bioequivalence trials of rifampicin containing formulations: extrinsic and intrinsic factors in the absorption of rifampicin.* Pharmacological Research, 2004. **50**(3): p. 317-327.
48. Kiser, J.J., et al., *Isoniazid pharmacokinetics, pharmacodynamics, and dosing in South African infants.* Ther Drug Monit, 2012. **34**(4): p. 446-51.
49. Ruslami, R., et al., *Pharmacokinetics and safety/tolerability of isoniazid, rifampicin and pyrazinamide in children and adolescents treated for tuberculous meningitis.* Archives of Disease in Childhood, 2022. **107**(1): p. 70-77.
50. Stott, K.E., et al., *Pharmacokinetics of rifampicin in adult TB patients and healthy volunteers: a systematic review and meta-analysis.* Journal of Antimicrobial Chemotherapy, 2018. **73**(9): p. 2305-2313.
51. Smith, P.B., et al., *Rifampin Pharmacokinetics and Safety in Preterm and Term Infants.* Antimicrobial Agents and Chemotherapy, 2019. **63**(6): p. e00284-19.
52. Takano, R., et al., *Oral absorption of poorly water-soluble drugs: computer simulation of fraction absorbed in humans from a miniscale dissolution test.* Pharm Res, 2006. **23**(6): p. 1144-56.
53. Boxenbaum, H.G. and S. Riegelman, *Determination of isoniazid and metabolites in biological fluids.* Journal of pharmaceutical sciences, 1974. **63**(8): p. 1191-7.
54. Peloquin, C.A., et al., *Pharmacokinetics of isoniazid under fasting conditions, with food, and with antacids.* The international journal of tuberculosis and lung disease : the official journal of the International Union against Tuberculosis and Lung Disease, 1999. **3**(8): p. 703-10.
55. Hutchings, A.D., et al., *Saliva and plasma concentrations of isoniazid and acetylisoniazid in man.* Br J Clin Pharmacol, 1988. **25**(5): p. 585-9.

56. Pepin, X., M. Goetschy, and S. Abrahmsén-Alami, *Mechanistic models for USP2 dissolution apparatus, including fluid hydrodynamics and sedimentation.* Journal of Pharmaceutical Sciences, 2021.
